# A comparative evaluation of genome assembly reconciliation tools

**DOI:** 10.1186/s13059-017-1213-3

**Published:** 2017-05-18

**Authors:** Hind Alhakami, Hamid Mirebrahim, Stefano Lonardi

**Affiliations:** Department of Computer Science & Engineering, University of California, 900 University Avenue, Riverside, 92521 CA USA

**Keywords:** De novo genome assembly, Genomics, Assembly reconciliation

## Abstract

**Background:**

The majority of eukaryotic genomes are unfinished due to the algorithmic challenges of assembling them. A variety of assembly and scaffolding tools are available, but it is not always obvious which tool or parameters to use for a specific genome size and complexity. It is, therefore, common practice to produce multiple assemblies using different assemblers and parameters, then select the best one for public release. A more compelling approach would allow one to merge multiple assemblies with the intent of producing a higher quality *consensus* assembly, which is the objective of *assembly reconciliation*.

**Results:**

Several assembly reconciliation tools have been proposed in the literature, but their strengths and weaknesses have never been compared on a common dataset. We fill this need with this work, in which we report on an extensive comparative evaluation of several tools. Specifically, we evaluate contiguity, correctness, coverage, and the duplication ratio of the merged assembly compared to the individual assemblies provided as input.

**Conclusions:**

None of the tools we tested consistently improved the quality of the input GAGE and synthetic assemblies. Our experiments show an increase in contiguity in the consensus assembly when the original assemblies already have high quality. In terms of correctness, the quality of the results depends on the specific tool, as well as on the quality and the ranking of the input assemblies. In general, the number of misassemblies ranges from being comparable to the best of the input assembly to being comparable to the worst of the input assembly.

**Electronic supplementary material:**

The online version of this article (doi:10.1186/s13059-017-1213-3) contains supplementary material, which is available to authorized users.

## Background

Despite the prodigious throughput of the sequencing instruments currently on the market, the assembly problem remains very challenging, mainly due to the repetitive content of large genomes, uneven sequencing coverage, and the presence of (non-uniform) sequencing errors and chimeric reads. The third generation of sequencing technology, e.g., Pacific Biosciences [1] and Oxford Nanopore [2], offers very long reads at a higher cost per base, but the sequencing error rate is much higher.

A significant number of de novo genome assemblers are available to the community. The choice of the most appropriate assembler depends on the size and complexity (repeat content, ploidy, etc.) of the genome to be assembled, the type of sequencing technology used to produce the input reads (e.g., Sanger, 454, Illumina, PacBio, Nanopore, etc.), and the availability of paired-end or long-insert mate-pair reads. Each assembler implements slightly different heuristics to deal with repetitions in the genome, uneven coverage, sequencing errors and chimeric reads. The final assembly is very rarely entirely finished, with one solid sequence per chromosome. Instead, the typical output is an unordered/unoriented set of contiguous regions called *contigs*. If paired-end or mate-pair reads are available, some of the contigs can be ordered and oriented by anchoring paired-end reads to contigs. In some cases, the length of the gaps between contigs can be estimated and contigs can be joined together to create *scaffolds*.

As said, selecting which assembler to use to produce the best quality assembly is not a trivial task. Assembly competitions such as the Genome Assembly Gold-Standard Evaluation (GAGE) [3] and Assemblathon [4] have been held to evaluate multiple assemblers on common datasets. Such comparative evaluations can provide general guidelines, but there is no systematic way to determine which assembler and what parameter settings to use to produce the best assembly for a specific genome and a specific dataset. As a consequence, it is common practice to generate multiple genome assemblies from a few different assemblers and/or parameters (e.g., the *k*-mer size for the de Bruijn graph), and then try to guess the best assembly based on assembly statistics, spot-checking, homology analysis, agreement with physical, genetic, or optical maps, etc.

In fact, the notion of best assembly is not well defined. Since it is unlikely that one can obtain a perfect assembly that covers the entire genome with no assembly errors, one has to decide whether it is more important to maximize contig and scaffold length (at the expense of possibly introducing more misassemblies) or minimize the number of misassemblies (at the expense of possibly generating shorter contigs and scaffolds). Typically, the quality assessment for draft assemblies is carried out via statistical measurements and alignment to a reference genome (if one is available). N50 is a metric widely used to assess the contiguity of an assembly, which is defined by the length of the shortest contig for which longer and equal length contigs cover at least 50 *%* of the assembly. NG50 resembles N50 except the metric relates to the genome size rather than the assembly size. NA50 and NGA50 are analogous to N50 and NG50 where the contigs are replaced by blocks that can be aligned to the reference. Correctness is measured by detecting misassemblies such as mismatches, indels, and misjoins. Misjoins are considered the least desirable type of misassemblies [5], where loci that are far apart in the genome are improperly joined in the assembly. Misjoins include inversions, relocations, and translocations. An *inversion* occurs when the orientation of a contig is inverted with respect to the reference. A *relocation* occurs when a contig is misplaced within the chromosome it belongs to, and a *translocation* occurs when a contig is misplaced into a different chromosome.

Assembly reconciliation algorithms attempt to take one step further toward a finished genome. Rather than arbitrarily trying to guess the best assemblies among several draft assemblies, assembly reconciliation tools offer compelling alternatives. These tools promise to produce a higher quality *consensus* assembly by merging two or more draft assemblies. The main goal of assembly reconciliation algorithms is to enhance the contiguity of the resulting assembly while at the same time avoiding the introduction of assembly errors. In this paper, we carry out the first comprehensive evaluation of assembly reconciliation tools by measuring the quality of the consensus assembly on several common input datasets with different quality attributes.

### Assembly reconciliation tools

The concept of assembly reconciliation was first introduced by Zimin et al. [6]. In that work, the authors also introduced an assembly reconciliation tool called RECONCILIATOR, which is no longer maintained (last updated in 2007). Other reconciliation tools in the literature that are no longer maintained and/or have no documentation were excluded from our evaluation. We also excluded GAM, because it was superseded by GAM_NGS. Other tools such as eRGA [7], MAIA [8], RAGOUT [9], and Minimus2 [10] were also not included in our comparative evaluation because these tools address different problems. Reference-guided assembly (eRGA, RAGOUT, and MAIA) and hybrid assembly (Minimus2) are related to the problem of assembly reconciliation, but it is not quite the same. The former uses a closely related reference to assemble the conserved regions of the genome, which reduces the complexity of de novo assembly to the non-conserved portions. Hybrid assembly allows users to incorporate reads from different sequencing technologies (e.g., short Illumina reads with long PacBio reads). MAIA has also the ability to merge de novo assemblies if several closely related reference genomes are available. QuickMerge [11] is a tool that allows users to merge an assembly obtained from Pacific Bioscience reads with another assembly based on second-generation reads. We excluded QuickMerge from our evaluations due to the lack of publicly available PacBio-based assemblies with a corresponding high-quality reference genome that would allow us to assess the results.

In this work, we benchmarked seven assembly reconciliation tools, namely CISA, GAA, GAM_NGS, GARM, Metassembler, MIX, and ZORRO, which are briefly described next. Table 1 summarizes the main goals and features of the seven assembly reconciliation tools. Several of these algorithms use the *compression–expansion* (CE) statistic to detect assembly compression (due to an incorrect deletion) or assembly expansion (due to an incorrect insertion) [6]. To obtain the CE statistic, paired-end or mate-pair reads are mapped to the assembly to be evaluated. The CE statistic is computed by comparing the distance between the mapped mates and the expected insert size.

**Table 1:** Features of the assembly reconciliation tools evaluated in this study

	CISA	GAA	GAM_NGS	GARM	Metassembler	MIX	ZORRO
Inputs							
Contigs allowed	✓	✓	✓^a^	✓	✓	✓	✓
Scaffolds allowed	✓	✓^b^	✓^a^	✓	✓		
Short reads allowed			✓^a^				
Paired-end reads allowed			✓^a^				✓
Mate-pair reads allowed			✓^a^		✓		
Alignments allowed		✓	✓				
Reads required			✓^a^		✓		✓
Reference input assembly required			✓		✓		
Input assemblies treated symmetrically	✓					✓	
Only two input assemblies		✓	✓	✓	✓^c^		✓
More than two input assemblies	✓					✓	
Can handle bacterial/small genomes	✓	✓	✓	✓	✓	✓	✓
Can handle large eukaryotic genomes		✓	✓	✓	✓		✓
Goals							
To increase assembly contiguity	✓	✓	✓	✓	✓	✓	✓
To decrease number of assembly errors	✓		✓				
Methods							
Compression-expansion statistic		✓	✓		✓		
Scaffolding information		✓		✓			
Use single reads			✓				
Use paired-end/mate-pair reads		✓	✓		✓		✓
Can split assembly misjoin	✓	✓					
Can detect/avoid repetitive regions	✓		✓				✓
Output							
Contigs				✓	✓	✓	
Scaffolds	✓	✓	✓	✓^d^	✓	✓	✓

^a^Optional, as GAM_NGS requires an alignment file

^b^Scaffolds should be broken into contigs. A gap file and contig naming convey scaffolding information

^c^Performs iterative pairwise

^d^When the input contains scaffolds

The objective of CISA is to reconcile bacterial genome assemblies [12]. Given the contigs for each of the input draft assemblies, CISA selects *representative* contigs (i.e., the longest contigs) and discards (nearly) contained contigs. CISA then tries to extend representative contigs, and detects misassembly in the representative contigs by aligning them to query contigs. Contigs that align to multiple positions are considered misassembled and another representative contig is selected. Contigs with an unaligned portion are split. Finally, the resulting contigs are iteratively merged. We should note that CISA’s objective is to merge more than two assemblies, but we have also tested it on two inputs for consistency with other tools. CISA was used in [13] and [14] to merge assemblies produced by three different assemblers. In [15], it was used to integrate multiple Newbler (454/Roche) assemblies.

Users of GAA have to specify a target and a query assembly [16] where the target assembly is expected to be of higher quality. The objective of GAA is to close gaps in the target assembly using the query assembly. Query contigs that are not anchored to at least two contigs target are disregarded. GAA was used (i) in [17] to merge a SOAPdenovo assembly with a Newbler assembly, (ii) in [18] to merge a Newbler assembly with a PCAP assembly [19], and (iii) in [20] to merge an ABySS assembly with a CLC assembly [21].

The input to GAM_NGS is one or more alignments between each library of reads and each input assembly [22]. GAM_NGS first identifies maximal portions of both input assemblies (called *blocks*) that share the same set of uniquely mapped reads. GAM_NGS then constructs a weighted undirected graph where each vertex corresponds to a contig, and an edge connects two contigs if (i) they belong to different assemblies and (ii) they share at least one block. From this graph, GAM_NGS computes a consistent ordering and orientation of blocks with respect to both input assemblies. Then, GAM_NGS builds another directed weighted graph (called the *assembly graph*) where each vertex represents a block, and each edge connects two blocks if they belong to the same contig. After resolving conflicts in the *assembly graph*, GAM_NGS computes a semi-global alignment between any two contigs that share at least one block. If two contigs have at least 95 *%* identity, GAM_NGS merges the assemblies by selecting the assembly with the best CE statistic. GAM_NGS was used in [23] to merge three assemblies created using Velvet-SC [24], SPAdes [25], and IDBA-UD [26]. In [27], it was used to merge a Newbler assembly with a SOAPdenovo assembly.

GARM [28] also manipulates assemblies asymmetrically, but users do not need to know in advance which one is the better assembly. The tool decides which is the reference assembly based on a variety of assembly statistics. GARM then (i) aligns the assemblies to each other to detect overlaps (using *nucmer* [29]), (ii) removes ambiguous overlaps and contigs that are (nearly) completely contained in each another, (iii) generates layout and consensus scores, (iv) merges contigs, and (v) orders merged contigs to match the order and the orientation of the original scaffolds (if scaffolds are available). If a contig that is a part of a scaffold is not merged, the contig is placed within the resulting scaffold in a location that corresponds to the original scaffold and the gap length is recomputed. GARM was used in [30] to merge a IDBA-UD assembly with a Newbler assembly.

CE statistics on the two input assemblies are also used in Metassembler [31]. First, Metassembler uses *nucmer* [29] to align the two input assemblies. The boundaries of these alignments are called *break points*. For each region between the break points, one of two assemblies is selected based on its CE statistic. Metassembler allows users to input more than two assemblies, but merges them in a progressive pairwise fashion. In [32], Metassembler was used to merge an ALLPATHS-LG assembly with an assembly based on Illumina Moleculo [33] synthetic long reads.

MIX [5] uses a directed weighted graph called an *extension graph*, which is annotated with a variety of weights to represent prefix–suffix overlaps between contigs in the input assemblies. MIX determines a set of non-overlapping *maximal independent longest paths* on the extension graph to merge contigs. Contigs not included in any path are examined for duplications. Contigs that are contained or nearly contained are removed, and the rest are added to the assembly. MIX does not perform error correction, but rather focuses on enhancing contiguity. MIX was used in [34] and [35] to merge two assemblies and in [36] to merge three assemblies.

ZORRO [37] starts by masking repetitive regions, which are identified using *k*-mer statistics. Once the repetitive regions are masked, the overlap between the two assemblies is detected using Minimus [10]. ZORRO then unmasks the repetitive regions and merges overlapping contigs. Lastly, ZORRO uses the tool Bambus [38] to order and orient contigs using paired-end reads. ZORRO was used in [39] and [40] to merge two assemblies.

While one would expect assembly reconciliation tools to be particularly beneficial for large complex (eukaryotic) genomes, many bacterial genome assembly projects have taken advantage of these tools. For instance, CISA was used in the assembly of *Xylella fastidiosa* [13] and *Paenibacillus polymyxa* [14]. MIX was employed for *Acidibacillus ferrooxidans* [34], *Piscirickettsia salmonis* [35], and *Cupriavidus sp.* Strain SK-3 [36]. GAM_NGS was used in the assembly of *Coxiella burnetii* [23], GARM was used for *Wolbachia* [30], and ZORRO was used for *Desulfurella amilsii* [39].

## Results

### Datasets and experimental results

Since the quality of the input assemblies is expected to directly affect the quality of the final merged assembly, we explored the performance of assembly reconciliation tools under different input quality.

To carry out a comparative evaluation of the seven assembly reconciliation tools listed above, we used publicly available assemblies created for the GAGE competition [3] and we created synthetic assemblies of *Saccharomyces cerevisiae S288c* [37] including structural variants. The motivation for this choice of the GAGE assemblies was that this dataset has been the most commonly used for assembly reconciliation tools. The authors of GAM_NGS used this dataset in their experimental results, CISA was tested on assemblies of *Staphylococcus aureus* and *Rhodobacter sphaeroides*, and MIX used GAGE_B [41], which includes the assemblies of *S. aureus* and *R. sphaeroides*. Other assembly reconciliation tools used the Assemblathon dataset [4], which was a similar assembly competition to GAGE. For instance, Metassembler used both the Assemblathon 1 and Assemblathon 2 datasets.

**Fig. 1:**
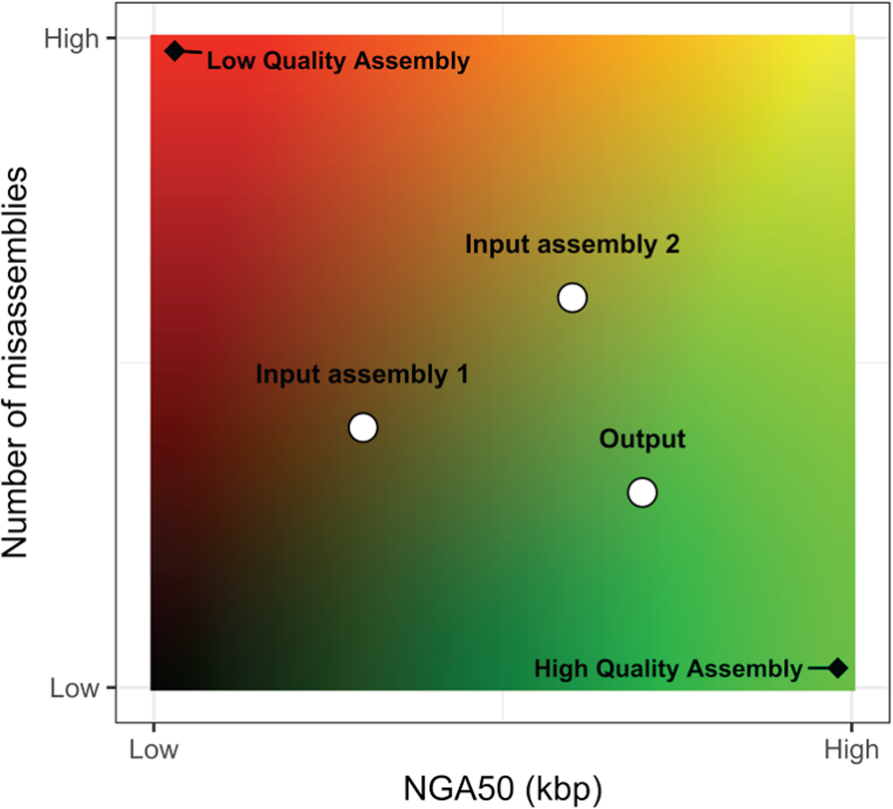
Performance of assembly reconciliation algorithms summarized as points on a 2D scatter plot. The *x*-axis represents contiguity (NGA50) and the *y*-axis is the number of misassemblies. In this example, input assembly 1 has fewer assembly errors than assembly 2, but assembly 2 is more contiguous. The output assembly is better than both inputs

**Fig. 2:**
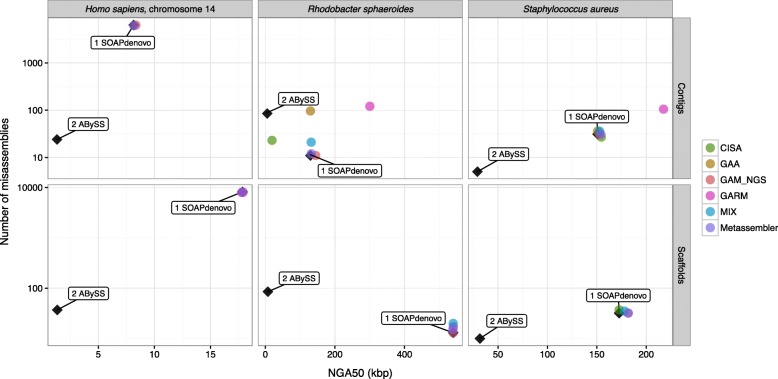
Contiguity–correctness experimental results. Inputs are contigs (*top row*) or scaffolds (*bottom row*). Assembly reconciliation tools are given two assembled genomes to merge (from *Homo sapiens*, chromosome 14, *Rhodobacter sphaeroides*, or *Staphylococcus aureus*), in which the first assembly has high contiguity, the second has high correctness. The tools were run using default parameters

**Fig. 3:**
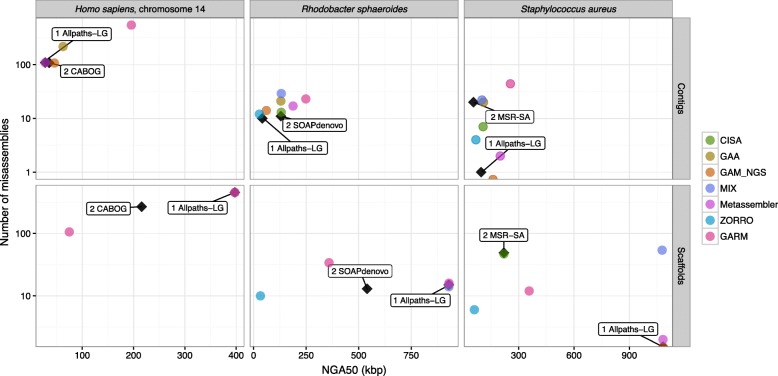
Experimental results on merging high-quality assemblies (*top row* for input contigs and *bottom row* for input scaffolds). Tools were run using default parameters

**Fig. 4:**
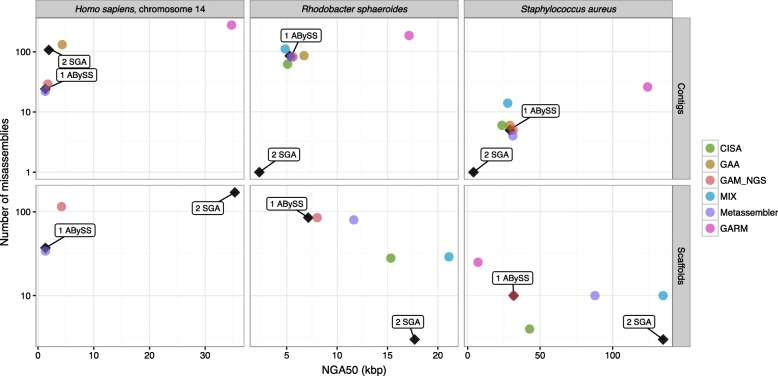
Experimental results on merging highly fragmented assemblies (*top row* for input contigs and *bottom row* for input scaffolds). Tools were run using default parameters

**Fig. 5:**
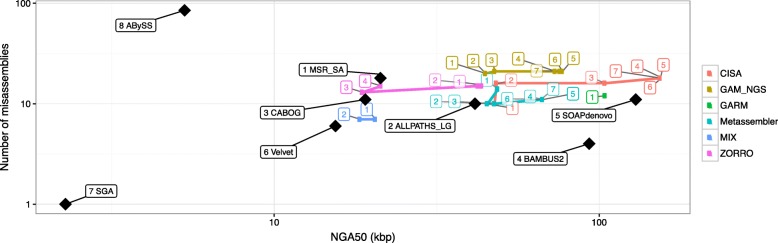
Experimental results on merging multiple assemblies of *Staphylococcus aureus* (*black diamonds*). The input order was determined using the feature response score (see text for details). Integer labels indicate successive merging steps. Tools were run using default parameters

All assembly reconciliation tools were run with default parameters, and Quast [42] was used to gather extensive assembly statistics (see Methods for details). A complete report on all these statistics is reported in Additional file 1: Note 3 and Additional file 1: Table S1–S19. Here, we only summarize the results using a graphical representation of the contiguity/correctness tradeoff. Input and output assemblies are represented as points on the scatter plot where the x-coordinate represents the contiguity (NGA50), and the y-coordinate is the number of misassemblies. Figure 1 illustrates how to interpret the plots. We expect assembly reconciliation tools to “move” the input points towards the bottom right corner of the plot, i.e., increase the contiguity and reduce the number of assembly errors (as shown in the example in Fig. 1). Experimental results are summarized in Figs. 2, 3, 4 and 5.

A detailed analysis of run time, memory consumption, CPU utilization for all the tools is available in Additional file 1: Note 4. A companion website http://reconciliation.cs.ucr.edu/ provides links to the all the datasets and the scripts used in this study.

### GAGE assemblies

The GAGE competition evaluated eight assemblers (ABySS [43], ALLPATHS-LG [44], Bambus2 [38], Celera Assembler [45, 46], MaSuRCA [47], SGA [48], SOAPdenovo [49], and Velvet [50]) on whole-genome shotgun sequence data of four genomes, namely *S. aureus* (genome size ≈2.8 Mbp), *R. sphaeroides* (≈4.6 Mbp), *Homo sapiens* chromosome 14 (≈88 Mbp), and *Bombus impatiens* (≈250 Mbp). *S. aureus* has one main chromosome and a small plasmid, while *R. sphaeroides* has two chromosomes and five plasmids. In our experiments, we mainly used the first three genomes, because at the time of writing *Bombus impatiens* did not have a high-quality reference genome. We used the assemblies for *Bombus impatiens* only to determine which tools would be able to handle large inputs (see Additional file 1: Note 5). Out of the 4×8 genome-assembler pairs, the GAGE competition included 27 assemblies (available from http://gage.cbcb.umd.edu).

Running each assembly reconciliation tool on all pairs of assemblies (out of the 27 available) would generate several hundred merged assemblies and it would be difficult to draw general conclusions. We decided instead to select input assembly pairs based on six different criteria and compare the results on the selected pairs. To streamline the presentation, we will not comment on tools that did not run successfully. Other practical limitations related to the execution of these tools are reported in Additional file 1: Note 6. Finally, some of tools can take advantage of the raw reads, in addition to the corresponding assemblies. The use of raw reads is explained in Additional file 1: Note 7.

#### High-contiguity and high-correctness inputs (GAGE)

In the first set of experiments, the objective was to explore the contiguity/correctness tradeoff. Specifically, we wanted to test the ability of reconciliation tools to take advantage of the contiguity of the first input assembly and the correctness of the second to create a merged assembly with a number of misassemblies comparable to the second assembly and a contiguity comparable to the first assembly. The two input assemblies to be merged were chosen so that one had high N50 value (but possibly a relatively high number of misassembly errors) and the other had few misassembly errors (and possibly a lower N50).

Figure 2 and Additional file 1: Table S4 report the results of merging the SOAPdenovo assembly (high N50) with the ABySS assembly (low misassembly errors) for the three chosen genomes. Since the assembly produced by ABySS on the *R. sphaeroides* genome has more misassembly errors than the assembly generated by SOAPdenovo, we reported in Additional file 1: Table S5 the results on ALLPATHS-LG and SGA’s assembly of *R. sphaeroides*. The SOAPdenovo assembly was used as the master assembly in all tools that require a ranking of the inputs.

Observe in Fig. 2 that for the *S. aureus* genome, all tools increased the contiguity marginally (by less than 3 *%*). While none of the tools was able to improve assembly errors compared to the ABySS assembly, GAA and MIX produced more errors than SOAPdenovo. CISA produced the lowest number of misassemblies (27 misassemblies compared to 31 with SOAPdenovo). Otherwise, GAM_NGS and Metassembler maintained the quality statistics close to that of SOAPdenovo.

GAA created a merged assembly in which the number of misassemblies was very close to the sum of those statistics for the input assemblies. In terms of NGA50, the contiguity was at least as good as the most contiguous input assembly.

When the input was composed of scaffolds (Fig. 2 bottom row), all tools improved contiguity marginally (by less than 5 *%*). Additional file 1: Table S4 show that GARM’s and MIX’s merged assemblies covered less than 50 *%* of the reference sequence. None of the tools was able to reduce the number of misassembly errors compared to ABySS; in fact, CISA produced more errors than SOAPdenovo.

Despite that the ABySS assembly for *R. sphaeroides* had a higher number of errors than SOAPdenovo, none of the tools improved on the number of misassemblies compared to SOAPdenovo. With the exception of GAA, the number of misassembly errors produced by all tools was closer to the master (SOAPdenovo). As expected, tools that relied on a master assembly had a lower number of misassemblies than those that did not rank the inputs. With scaffolds as inputs, changes in NGA50 were negligible for all tools except for CISA. With contigs as inputs, GAM_NGS improved the contiguity by at most 11 *%*, Metassembler and MIX increased it by 2 *%*, and CISA dropped it by 85 *%*. MIX, Metassembler, and GARM maintained the same NGA50 as SOAPdenovo.

In the majority of cases, the experimental results obtained with ALLPATHS-LG (high N50) and SGA (low misassembly errors) on the *R. sphaeroides* genome (reported in Additional file 1: Table S5) followed similar patterns to the ones in Fig. 2. CISA decreased the contiguity (although the reduction was far less this time). GAA followed the same general pattern mentioned earlier. GAM_NGS did not increase contiguity but rather maintained it close to that of the master assembly. Metassembler and MIX also did not increase contiguity. ZORRO worked for this experiment: while it decreased contiguity by 10 *%*, it produced a smaller number of misassembly errors than ALLPATHS-LG (but still higher than SGA).

With scaffolds as inputs, GAM_NGS retained the quality statistics of the master assembly. Observe in Fig. 2 that GARM retained NGA50 close to SOAPdenovo (the master assembly). Also observe in Additional file 1: Table S5 that GARM maintained ALLPATHS-LG’s contiguity statistics.

The experimental results for *Homo sapiens*, chromosome 14 (Hg_chr14) with contigs as input assemblies (Fig. 2), show that (i) GAM_NGS slightly improved contiguity, (ii) Metassembler maintained the same contiguity, (iii) GAA crashed, and in general, (iv) the number of misassemblies was closer to SOAPdenovo. With scaffolds as inputs, GAM_NGS and Metassembler produced assemblies with quality statistics close to SOAPdenovo.

#### Reordering the inputs (GAGE)

As mentioned above, some of the assembly reconciliation tools assume that the first input assembly is the master assembly, and should be trusted more (we called these tools *asymmetric*). The goal of this set of experiments was to determine how the quality of the merged assembly depended on the specific input order.

To determine how the ranking affected the results, we repeated the same experiments reported in the previous section but switched the order of the inputs. A comparative analysis of Additional file 1: Figure S11 and Additional file 1: Table S6 with the results discussed in the previous section prompts a few observations. First, we note that CISA, MIX, and GARM are *symmetric* (i.e., they do not require users to rank the inputs, see Table 1), hence they are expected to be unaffected by the reordering. Experimental results confirm that CISA and GARM are indeed unaffected. The reordering, however, affected the MIX results, albeit only slightly.

For *S. aureus*, MIX’s contiguity statistics (N50 and NGA50) were not affected by the reordering of the inputs. However, we observed a small change in the number of misassemblies, although still higher than SOAPdenovo in both cases.

For *R. sphaeroides*, all statistics remained unchanged except for the number of misassemblies, which increased after reordering. In addition, with contigs as inputs, we did not observe an increase in NGA50 after the reordering.

Despite that GAA requires input ranking, the results for *S. aureus* and *R. sphaeroides* were similar in both orderings. The output statistics of GAA followed the general pattern mentioned in the previous section. For Hg_chr14, GAA crashed in one ordering but not on the other. For all three genomes, GAM_NGS and Metassembler produced consensus assemblies with quality statistics close to the master assembly.

Note that the merged assemblies have higher contiguity in Fig. 2, in which the master has higher N50. In contrast, the number of misassemblies was lower in Additional file 1: Figure S11 for both *S. aureus* and Hg_chr14, in which the master had lower errors (with the exception of MIX). Merged assemblies for *R. sphaeroides* had higher contiguity and a lower number of misassemblies when the master had higher N50 and a lower number of misassemblies (see Fig. 2).

#### High-quality inputs (GAGE)

In the third set of experiments, we tested the ability of the reconciliation tools to merge two high-quality assemblies. We selected two highly contiguous assemblies (i.e., a small number of contigs and scaffolds, high N50 values) and a low number of misassembly errors. Figure 3 and Additional file 1: Table S7 show the result of merging assemblies produced by ALLPATHS-LG as first input and either MSR-CA, SOAPdenovo, or CABOG as the second input assembly.

Observe that for *S. aureus* with contigs as inputs, GAM_NGS produced an improved assembly that had no misassemblies, and was 66 *%* more contiguous. The next best assembly was by Metassembler with a 107 *%* increase, but it had a slight increase in the number of misassemblies compared to ALLPATHS-LG. MIX produced a high number of misassemblies (higher than MSR-CA) but managed to increase contiguity by 4 *%*. CISA improved contiguity by 11 *%*, but it produced more errors than ALLPATHS-LG. ZORRO decreased contiguity by 30 *%*.

With scaffolds as inputs, ALLPATHS-LG had no misassemblies and a higher NGA50 than MSR-CA. In general, asymmetric tools produced a lower number of misassemblies and decreased the N50. For instance, GAM_NGS maintained the quality statistics of ALLPATHS-LG. Although ZORRO is asymmetric, it decreased contiguity by more than 90 *%*. On the other hand, symmetric tools had a higher number of misassemblies. GARM achieved the highest increase of NGA50 (16 *%*).

The contiguity of the merged assemblies improved 11–108 % with the exception of ZORRO, which decreased the contiguity by 30 *%*. GARM increased contiguity the most (108 *%*) at the expense of a number of misassemblies close to MSR-CA. MIX introduced no misassemblies, but covered only 25 *%* of the genome sequence. Notably, both GAM_NGS and Metassembler improved contiguity by 66.5 *%* and introduced no misassemblies. These are two rare examples in which we observed an unquestionable improvement in the merged assembly.

For the *R. sphaeroides* genome, the two input assemblies had almost the same number of misassemblies but the assembly produced by SOAPdenovo was much less fragmented. Only Metassembler increased NGA50 significantly. All other tools decreased the contiguity. In terms of correctness, ZORRO and CISA (using scaffolds as inputs) reduced the number of misassemblies but also decreased the contiguity, by 99 *%* and 60 *%*, respectively. Other tools produced merged assemblies with the number of misassemblies no better than the inputs.

GARM improved the contiguity by 38 *%* while CISA increased it by less than 2 *%*. MIX was the only tool that reduced the number of misassemblies, but again its assembly only covered about half of the genome. None of the tools improved both contiguity and the number of misassemblies.

For Hg_chr14, GAA improved the NGA50 by 76 *%*, but it produced a number of misassemblies equal to the sum of misassemblies in the inputs. GAM_NGS improved the contiguity (28 *%* increase in NGA50) and slightly reduced the number of misassemblies. Metassembler produced quality statistics that were very close to ALLPATHS-LG.

With scaffolds as inputs, GAM_NGS and Metassembler maintained similar quality statistics to ALLPATHS-LG. GARM decreased NGA50 by 9 *%*. It also increased the number of misassemblies.

#### Highly fragmented inputs (GAGE)

The goal of this set of experiments was to evaluate the performance of assembly reconciliation tools when provided with two highly fragmented input assemblies. Input assemblies were selected to have a high percentage of contigs shorter than 200 bps, a high number of contigs and scaffolds, and low N50.

Figure 4 and Additional file 1: Table S8 show the results of merging the ABySS assembly and SGA assembly. Observe that when we used contigs as inputs, ABySS had a higher contiguity than SGA (except for Hg_chr14). The opposite, however, was observed when scaffolds were provided as input. For *S. aureus* and *R. sphaeroides* with contigs as inputs, only asymmetric tools maintained or improved NGA50 of the better input assembly (for *S. aureus*, we observed up to an 8 *%* increase, and up to 17 *%* for *R. sphaeroides*). However, for Hg_chr14 (with contigs as inputs), GAA produced a 123 *%* increase over SGA, while GAM_NGS did not improve NGA50 over SGA, but it increased it 33 *%* over ABySS.

With scaffolds as inputs, we observed a decrease in NGA50 except for MIX and GARM (when SGA inputs are scaffolds). MIX, GARM, and CISA are symmetric tools, hence they were expected to perform better than other tools when the non-master input has better quality. CISA, however, produced inferior results with scaffolds as inputs in most experiments. We discovered that CISA with default parameters breaks scaffolds into contigs when a scaffold contains more than ten consecutive N’s. MIX maintained the NGA50 of SGA, while GARM slightly decreased it compared to SGA (yet it was still higher than ABySS).

#### De Bruijn vs string graph assembly (GAGE)

Here we tested the effect of merging assemblies generated using different assembly strategies. Specifically, we merged an assembly generated by an assembler that uses de Bruijn graphs with an assembly produced by an assembler based on the string graph. Additional file 1: Figure S12 and Table S5 show the result of merging an assembly produced by ALLPATHS-LG (based on a de Bruijn graph) with an assembly produced by SGA (based on a string graph). Overall, GAM_NGS, Metassembler, and MIX maintained similar assembly statistics as ALLPATHS-LG.

Note that *S. aureus* input assemblies (as contigs) had only one misassembly. The merged assemblies also had one misassembly, with the exception of GAA (two) and ZORRO (none). ZORRO corrected the assembly error without affecting NGA50. CISA decreased NGA50 by 49 *%*. With scaffolds as inputs, ALLPATHS-LG’s assembly had no assembly errors. In fact, observe that all merged assemblies did not have any misassemblies. GARM kept NGA50 close to that of ALLPATHS-LG. CISA covered less than 40 *%* of the genome, while ZORRO decreased the contiguity by 99 *%*.

For *R. sphaeroides* with contigs as inputs, CISA and ZORRO decreased the contiguity by 34 *%* and 10 *%*, respectively. GAM_NGS and Metassembler maintained ALLPATHS-LG’s quality statistics. All tools produced a relatively high number of misassemblies (like ALLPATHS-LG). With scaffolds as inputs, CISA, ZORRO, and GARM’s assembly statistics were like the statistics of *S. aureus*. All assemblies, with the exception of CISA and ZORRO, had the number of misassemblies closer to that of ALLPATHS-LG. CISA again covered less than one-fifth of the genome and ZORRO decreased the contiguity by 99 *%*. GAM_NGS, Metassembler, and MIX produced consensus assemblies with quality statistics comparable to that of ALLPATHS-LG.

For Hg_chr14 (with contigs as inputs), GAM_NGS increased NGA50 by 2 *%*. With scaffolds as inputs, GAM_NGS and Metassembler maintained the contiguity and number of misassemblies close to ALLPATHS-LG. GARM increased the number of misassemblies to 496 (compared to 455 with ALLPATHS-LG) and decreased NGA50 by 9 *%*.

#### Multiple inputs (GAGE)

In this set of experiments, we tested the ability of the tools to merge more than two assemblies. When an assembly reconciliation tool allowed no more than two assemblies as input (see Table 1 for a list), we merged them in an iterative fashion. For instance, to merge three assemblies, we first merged two assemblies, then merged the result to the third assembly. Metassembler uses a similar strategy: when the user provides multiple assemblies, the tool iteratively performs pairwise reconciliation, where the output of one iteration is the input of the next. We ordered the input assemblies based on the *feature response* curve (FR curve), which is an assembly quality metric proposed in [51]. The FR curve represents the dependency between contigs that contain no more than *τ* features and the corresponding genome coverage. The *x*-axis represents *τ* and the *y*-axis represent genome coverage: the steeper the curve, the better the assembly. We used the FR curves in [22] to determine the merging order of the GAGE assemblies, starting with the assemblies with highest quality. The results for an alternative ordering are discussed in Additional file 1: Note 3.7. and the corresponding Additional file 1: Tables S15–S18. For tools that allowed us to merge more than two assemblies (e.g., CISA and MIX), the merging was done in one step from the original assemblies. Here we were interested in measuring the contiguity and correctness of the resulting assemblies as the number of input assemblies increased.

Figure 5, Additional file 1: Figure S1, and Figure S2 show experimental results for *S. aureus*, *R. sphaeroides*, and Hg_chr14, respectively, when the inputs are contigs. First observe that in several cases, the process of iterative merging did not complete.

For *S. aureus* and *R. sphaeroides*, CISA generally improved the contiguity as the number of merged assemblies increased. The number of errors fluctuated over the iterations. GAA did not produce assembly files for the first iteration. Although GAA did not work for this particular ordering, it produced results for the alternative ordering reported in Additional file 1: Note 3.7.

For *S. aureus* and *R. sphaeroides*, GAM_NGS’s contiguity improved over successive iterations, but the number of misassemblies errors did not decrease (it remained close to the first master input in all iterations). For Hg_chr14, the number of misassemblies was also relatively high. GAM_NGS increased NGA50 by at least 70 *%* compared to CABOG.

For *S. aureus*, Metassembler’s contiguity improved over successive iterations, but the number of misassemblies also increased. For *R. sphaeroides*, Metassembler’s assembly did not improve after the fourth iteration. Note that NGA50 was lower for Bambus2 and SOAPdenovo. The number of misassemblies for Metassembler was about the average of the inputs. For Hg_chr14, the number of misassembly errors was low and decreased over successive iterations. The contiguity was high, but slightly decreased over successive iterations.

MIX maintained a low number of misassemblies in most iterations but suffered from relatively poor NGA50. Since the genome coverage in most iterations was less than 50 *%*, no NGA50 was reported for those iterations. For the *S. aureus* genome, the coverage was less than 50 *%* in all iterations but it steadily improved with increasing number of inputs. For *R. sphaeroides*, the genome coverage was below 50 *%* with four or more inputs.

ZORRO frequently failed to produce results. When it worked, the contiguity usually started high, then fluctuated over successive iterations. ZORRO produced a relatively high number of misassemblies, between the number of misassemblies of the inputs.

We repeated the same experiment but with scaffolds as inputs. The results are reported in Additional file 1: Tables S12, S13, and S14 and Additional file 1: Figures S3, S4, and S5. CISA’s results show that after a certain number of input assemblies, increasing the number of inputs did not affect the results significantly. From that point forward, it generally improved the contiguity and reduced the number of contigs as the number of merged assemblies increased. The number of misassemblies was within the range of input assemblies. CISA reached stability with four inputs for *S. aureus* and three inputs for *R. sphaeroides*.

For *S. aureus*, MIX produced a high number of misassemblies, which generally increased as the number of inputs increased. It maintained high genome coverage. It also maintained high contiguity except for the last iteration. For *R. sphaeroides*, the number of misassemblies was also relatively high but it fluctuated as the number of inputs increased. It also maintained high contiguity, achieving the best NGA50 for less than five inputs. ZORRO produced a low number of misassemblies for *S. aureus* and *R. sphaeroides*. Contiguity was poor and generally decreased over successive iterations.

GAM_NGS maintained results very close to the first input throughout all iterations for *S. aureus*, *R. sphaeroides*, and Hg_chr14. In the latter genome, GAM_NGS’s contiguity generally improved in successive iterations but so did the number of misassemblies.

Metassembler maintained similar quality statistics to CABOG for Hg_chr14. For *R. sphaeroides*, Metassembler also maintained CABOG’s quality statistics with a slight decrease in the number of misassemblies and contiguity as the number of iterations increased. For *S. aureus*, Metassembler also maintained quality statistics close but not identical to those of MSR-CA. In general, as the number of inputs increased, the number of misassemblies slightly decreased and the contiguity slightly improved.

### Synthetic assemblies

In this set of experiments we tested assembly reconciliation tools on synthetic assemblies of *Saccharomyces cerevisiae* embedded with specific structural variations (see Methods for details). Decipher [54] was used to generate synteny plots displayed as gradients. When reference and query disagree, the gradients are interrupted. Gray regions indicate blocks that do not match the reference.

In each experiment we merged two inputs, namely (1) chromosomes 4 and 15 of the yeast genome and (2) a flawed version of (1) produced by RSVSim containing one structural variation, i.e., either a deletion, an inversion (reversal), or a translocation. RSVSim does not allow de novo insertions. For asymmetric tools, the flawed assemblies were used as the master assemblies to model the worst case. We introduced deletions and inversions of various sizes (50, 100, 200, and 500 kbp) into chromosome 4, and generated translocations of various sizes (again, 50, 100, 200, and 500 kbp) from chromosome 4 into chromosome 15.

**Fig. 6:**
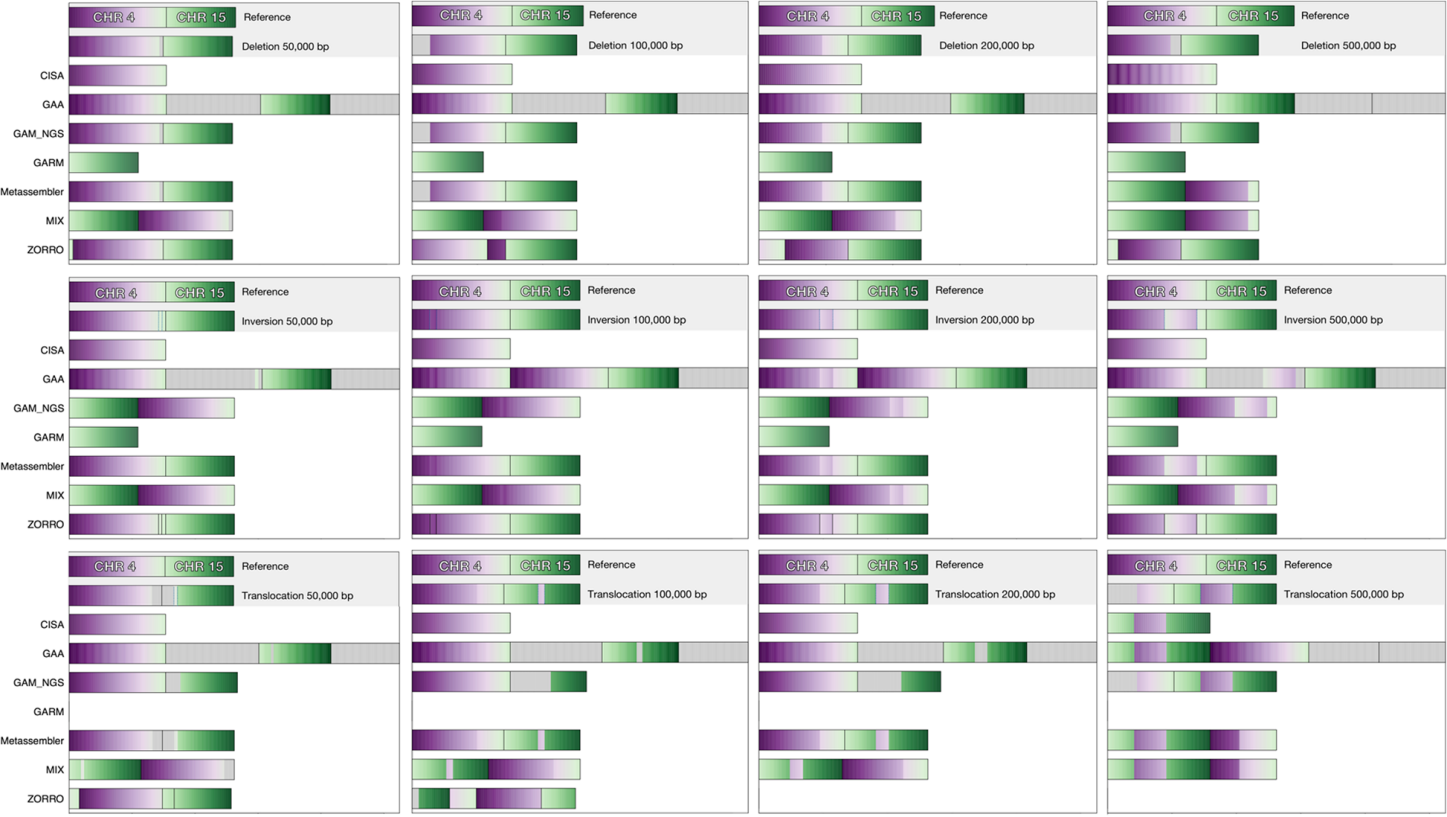
Results for the eight assembly reconciliation tools. They were given as input (1) chromosomes 4 and 15 of the yeast genome and (2) a flawed version of (1) produced by RSVSim containing a deletion in chromosome 4 (*top row*), an inversion in chromosome 4 (*middle row*), or a translocation from chromosome 4 to chromosome 15 (*bottom row*). (1) and (2) are the first two rows in each plot. Decipher was used to detect synteny blocks between the reference and the outputs and to generate synteny plots displayed as gradients. When the reference and output disagree, the gradients are interrupted. *Gray regions* indicate blocks that do not match the reference

Figure 6 (top row) show that CISA resolved the deletion but did not output chromosome 15. It produced two extra sequences that did not align to the reference. GARM did not output chromosome 4. GAM_NGS, Metassembler, and MIX produced assemblies like the flawed input assembly. ZORRO broke the assembly at the position of the deletion, produced three individual contigs, and omitted the deleted sequence.

Figure 6 (middle row) shows that only CISA resolved the inversion but did not output chromosome 15. GAA did not correct the inversion, and generated a merged assembly that was like the flawed input assembly with two additional sequences that did not align to the reference. Again, GAM_NGS, Metassembler, and MIX produced assemblies like the flawed assembly. ZORRO broke the inversion by producing three contigs for chromosome 4, and an additional contig representing chromosome 15.

For translocations, the behavior of the reconciliation tools depended on the size of the translocation, as shown in Fig. 6 (bottom row). For translocations of 50, 100, and 200 kbp, CISA, GAA, and GAM_NGS produced the correct version of chromosome 4. CISA did not produce chromosome 15 and GAA and GAM_NGS produced chromosome 15 with an unaligned sequence at the location of the insertion. As before, GAA produced two additional sequences. GARM did produce any merged assembly. Metassembler’s and MIX’s outputs were like the flawed input assembly. ZORRO split the assembly over structural variation breaking points. For 200 and 500 kbp, ZORRO was stopped after allocating more than 350 GB of RAM. None of the tools managed to correct the 500 kbp translocations. CISA and GAA produced the flawed version of chromosome 15. Again, GAA produced the correct version of chromosome 4, but two extraneous sequences. GAM_NGS’s output was very like the input flawed assembly. Metassembler and MIX produced chromosome 4 without the deleted fragment and a flawed version of chromosome 15.

To test whether read coverage had any impact on the quality of merged assemblies for assembly reconciliation tools that require reads as input, we ran several experiments on the same synthetic assemblies with increasing read fold coverage (15 × to 75 ×). Additional file 1: Figure S13 shows that read coverage did not affect the quality of the results.

## Discussion and conclusions

Given the practical challenges of de novo assembly, the idea of assembly reconciliation is very appealing. One could generate multiple assemblies on the same dataset using various assembly tools and/or parameters, then use an assembly reconciliation tool to merge all the assemblies to obtain a high-quality consensus assembly. Regarding the outcome, the expectation is that the quality of the merged assembly should be at least as good as the best assembly in the input. In fact, if both input assemblies have some good quality assembly statistics (e.g., one is more contiguous while the other has fewer misassemblies), one should expect the consensus assembly to inherit the good qualities from both inputs. The reality is that it seems very hard to produce a merged assembly that is consistently better than (or at least as good as) both input assemblies. The extensive set of experiments reported in this manuscript showed that none of the tools we evaluated was able to consistently achieve this goal. There were a few cases in which the consensus assembly was better than both inputs, but for the vast majority of the inputs, the merged assembly was not.

Despite the inability of these assembly tools to solve the general assembly reconciliation problem, each tool demonstrated some strengths that could lead to algorithmic advances for this problem. For instance, CISA generally was able to correct most structural variations and to ignore duplications in the input assemblies (however, its duplication rate increased as the number of merged assemblies increased). GAA and GARM often improved the contiguity (but often introduced more misassembly errors and increased the duplication ratio). GAM_NGS typically produced consensus assemblies very close to the quality of the reference (but not much better), and it was able to resolve translocations. MIX generally improved the contiguity modestly (but its number of misassemblies was usually close to or higher than the most erroneous input, and its genome coverage dropped in some cases). Metassembler often produced a consensus assembly with a very low number of misassembly errors, sometimes even lower than both input assemblies (however, it did not consistently increase N50). Finally, ZORRO generally maintained a high genome coverage (but it did not significantly increase contiguity).

## Methods

All experiments were performed on a Linux Ubuntu 12.10 server with a 20 cores Intel Xeon CPU 3GHz and 512GB of RAM. Multithreading was used when available.

Assembly reconciliation tools were ran with default parameters, unless otherwise noted. In Additional file 1: Note 1 and corresponding Tables in Additional file 1, we explored how other parameter settings affected the experimental results. Since some assembly reconciliation tools can take advantage of scaffold information, we carried out experiments on both contig-based assemblies and scaffold-based assemblies.

Outputs of assembly reconciliation tools were processed by our scripts, then fed into Quast [42] (GAGE option activated) to obtain assembly statistics. We collected assembly statistics related to contiguity, namely N50, number of contigs, longest contig, and total assembly size. By comparing the assemblies to the reference genome we also collected NGA50, number of misassemblies, the total length of contigs affected by misassemblies, the number of mismatches and indels between the assembly and the reference, the percentage of the reference genome covered by the assembly, and the duplication ratio. Quality scores were computed using Quast on the input assemblies. In addition to genome-wide analyses, we also studied the ability of assembly reconciliation tools to assemble the primary sequence of annotated genes. Specifically, we computed the fraction of each gene sequence covered by contigs, for both input and merged assemblies. Details about the procedure used to compute gene coverage can be found in Additional file 1: Note 2.

Synthetic assemblies were generated using RSVSim [52]. We used RSVSim to introduce specific structural variations into the reference genome of *Saccharomyces cerevisiae* [37]. For tools that required reads, we generated synthetic reads using ART [53]. The output of the seven assembly reconciliation tools was fed into Decipher [54]. Decipher detects synteny blocks between a reference and a query sequence, and generates synteny plots displayed as gradients.

## Additional file

Additional file 1 Contains Supplementary Notes 1–7, Supplementary Tables 1–19 and Supplementary Figures 1–13. (PDF 1250 kb)

## Abbreviations

CE statistic: Compression– expansion statistic
CISA: Contig integrator for sequence assembly
FR curve: Feature response curve
GAA: Graph accordance of assembly
GAGE: Genome Assembly Gold-Standard Evaluation
GAM_NGS: Genomic assemblies merger for next generation sequencing
GARM, Genome assembly: reconciliation and merging
Hg_chr14: *Homo sapiens*, chromosome 14
N50: length of the shortest contig for which longer and equal length contigs cover at least 50 *%* of the assembly
NG50: length of the shortest contig for which longer and equal length contigs cover at least 50 *%* of the genome
NA50 and NGA50: analogous to N50 and NG50 where the contigs are replaced by blocks that can be aligned to the reference
Quast: Quality assessment tool for genome assemblies
SGA: String graphs assembler
