# Supplementary material for: A comparative evaluation of genome assembly reconciliation tools
Source: Genome Biol. 2017 May 18;18:93. doi: 10.1186/s13059-017-1213-3 (PMC5436433; doi:10.1186/s13059-017-1213-3)
Supplement: Additional file 1 — Contains Supplementary Notes 1–7, Supplementary Tables 1–19 and Supplementary Figures 1–13. (PDF 1250 kb) [file 13059_2017_1213_MOESM1_ESM.pdf]

A Comparative Evaluation of Assembly Reconciliation Tools: Supplementary Material

Hind Alhakami, Hamid Mirebrahim and Stefano Lonardi

Supplementary Note 1: Parameter tuning

For the results in the Main Text and in Supplementary Note 3, all assembly reconciliation tools were ran with default parameters. In this Supplementary Note we explored how other parameter settings affected the experimental results. Each tool has its own set of parameters, as briefly described next.

- CISA has three main parameters namely the minimum contig cutoff, the maximum number of consecutive N's, and the maximum unaligned gap (default values 100 bp, 10 bp, 0.95 quintile, respectively); we changed the minimum contig cutoff to 200 bp and 500 bp and the maximum gap size to 100 and 200; we also tried scaffolds as inputs.
- For GAA we focused on two parameters, namely the minimum contig cutoff and the maximum tip size (default values of 100 bp and 90 bp, respectively); we changed the contig cutoff size to 200 bp and 500 bp and the maximum tip size allowed to 15 bp and 50 bp.
- GAM_NGS has three main parameters, namely the minimum number of reads to build a block, the block coverage filtering, and the minimum block length; for these parameters the authors suggest using 10 bp, 0.75, 200 bp, respectively for bacterial genomes, and 50 bp, 0.75, 500 bp for *Hg_chr14*; since there was no option to change the minimum block size, we explored the other two parameters; we used the default values of at least 50 reads per block and 0.75 block coverage filter; we also tried setting the read support to 10 and 30 with 0.75 block coverage filter, as well as read support of 10 and 50 with 0.95 block coverage filter.
- GARM: we explored changing the minimum contig cutoff (default 200 bp), minimum overlap (default 200 bp) and maximum tip thresholds (default 50 bp); in addition to the default values, we tried the following combinations (i) 500 bp contig cutoff, 200 bp min overlap, and 50 bp max tip, (ii) 200 bp contig cutoff, 100 bp min overlap, and 50 bp max tip (iii) 200 bp contig cutoff, 200 bp min overlap, and 100 bp max tip, and (iv) 100 bp contig cutoff, 50 bp min overlap, and 15 bp max tip.
- MIX's main parameters are the minimum length of alignment (default 0 bp) and minimum contig cutoff (default 500 bp); according to the documentation, if these two parameters are not specified MIX is supposed to check thresholds from 0 to 2000 with step of 50; we tried this option, but only got results with default settings; the author of MIX recommend a minimum alignment of 500 bp and a minimum contig cutoff of 0 bp for bacterial genomes (which is what we used); in addition we tried (i) minAlign=50 and minctg=100, (ii) minAlign=50 and minctg=200, and (iii) minAlign=100 and minctg=500.

- Metassembler has several parameters. We explored the parameters controlling the assembly merge phase, namely minimum read coverage (default 15), minimum overlap (default 60) and identity (default 60); Metassembler accepts identity in base pairs; we tested (i) 60 bp min overlap, 51 bp 85% identity, and 15x coverage (ii) 100 bp min overlap, 95% identity, and 15x coverage (iii) 100 bp min overlap, 85% identity, and 30x coverage and (iv) 200 bp min overlap, 170 bp 85% identity, and 30x coverage.
- ZORRO's parameters include the minimum overlap (default 40 bp), the maximum tip (default 15), and identity threshold (default 94%); in addition to the default values we tested (i) 40 bp min overlap, 100 bp max tip, and 94% identity, (ii) 100 bp min overlap, 15 bp max tip, and 94% identity, (iii) 100 bp min overlap, 15 bp max tip, and 85% identity, (iv) 100 bp min overlap, 50 bp max tip, and 85% identity.

Experimental results for all these parameter sets are reported in Supplemental Table S1, S2 and S3 for *S. aureus*, *R. sphaeroides* and *Hg_chr14*, respectively. For most experiments, the variations due to changing parameters were small. Few observations are in order.

Observe that for *S. aureus*, Metassembler and GAM_NGS maintained the same statistics for all parameters configurations, with the exception of a slight variation in the size of the assembly. CISA produced changes only when the minimum contig cutoff increased to 500 bp, with contigs as input. In this case, both genome and gene coverage improved but the contiguity decreased with respect to other configurations. With scaffolds as inputs, the contiguity increased but the genome fraction was lower than 50% in most cases. In GARM we observed a small variation in the number of mismatches and indels and an insignificant change in the genome coverage.

Supplementary Note 2: Gene coverage analysis

We used the following reference genomes and their corresponding gene annotations

- *S. aureus* subsp. aureus USA300_TCH1516 found at http://bacteria.ensembl.org/staphylococcus_aureus_subsp_aureus_usa300_tch1516/Info/Index (2,844 Genes)
- *R. sphaeroides* KD131 http://bacteria.ensembl.org/rhodobacter_sphaeroides_kd131/Info/Index (4,474 Genes)
- *Homo sapiens, chromosome 14* GRCh38.p2 http://uswest.ensembl.org/Homo_sapiens/Info/Index (ftp release 80) (2,289 Genes)

First, we created a BLAST database for each of the GAGE reference genome assemblies and each of the merged output assemblies. Then, we used BLASTn to align the primary sequence of each gene against each database (using default parameters). For each hit reported in BLASTn output, we chose the best ranked alignment with 75% minimum identity. The total gene coverage reported is the cumulative sum of the coverage of each hit minus any overlaps between the hits.

Supplementary Note 3: Experimental results on GAGE assemblies (observations on Supplemental Table S4 - S18)

3.1. High contiguity, high correctness inputs (GAGE)

In the first set of experiments, the objective was to explore the contiguity/correctness tradeoff. Specifically, we wanted to test the ability of reconciliation tools to take advantage of the contiguity of the first input assembly and the correctness of the second in order to create a merged assembly with a number of misassemblies comparable to the second assembly and a contiguity comparable to the first assembly. The two input assemblies to be merged were chosen so that one has high N50 value (but possibly a relatively high number of misassembly errors) and the other has few misassembly errors (and possibly a lower N50).

Supplemental Table S4 reports the results of merging the SOAPdenovo assembly (high N50) with the ABySS assembly (low misassembly errors) for the three chosen genomes. Since the assembly produced by ABySS on the *R. sphaeroides* genome has more misassembly errors than the assembly generated by SOAPdenovo we also considered the results on *R. sphaeroides* reported in Supplemental Table S5 where the input assemblies were produced by ALLPATHS-LG and SGA. The SOAPdenovo assembly was used as the "master" assembly in all tools that distinguish the assembly inputs.

Observe in Supplemental Table S4 that on the *S. aureus* genome, all tools increase the contiguity by less than 3%, although the number of contigs decreased by $7-30\%$ (except for GAA). While none of the tools was able to improve assembly errors compared to the ABySS assembly, GAA and MIX produced more errors than SOAPdenovo. CISA produced the lowest number of misassemblies (13% less than SOAPdenovo) at the cost of a 4% decrease in genome and gene coverage. Otherwise, GAM_NGS and Metassembler maintained quality statistics close to that of SOAPdenovo.

In this and the rest of the experiments below, GAA consistently produced assemblies with predictable statistics. In the vast majority of the cases, GAA created a merged assembly in which the number of contigs, the size of the resulting assembly, and the number of misassemblies were very close to the sum of those statistics for the input assemblies. GAA's gene coverage was typically low in *S. aureus* and *R. sphaeroides* (not as much on *Hg_chr14*, where the gene coverage was generally high in comparison to other merged assemblies), while the percentage of covered genome was relatively high. While GAA's N50 was low, in terms of NGA50 the contiguity was at least as good as the most contiguous input assembly. In fact for *Hg_chr14*, GAA increased NGA50 by $19-123\%$ except for one case in which the increase was negligible.

When the input was composed of scaffolds, all tools improved contiguity by less than 5%, and reduced the number of scaffolds by $12-92\%$, with GARM reporting the highest decrease. GARM was the only tool that significantly increased N50 and produced the lowest number of misassemblies; however, GARM's merged assembly covered less than 40% of the reference sequence and less than one third of the genes. In contrast, MIX's merged assembly covered 94% of the genes despite (i) including only about 44% of the reference genome and (ii) decreasing the contiguity by 48%.

If we exclude the number of contigs and NGA50, all the other assembly statistics for GAM_NGS and Metassembler are very similar to SOAPdenovo. None of the tools was able to reduce the number of misassembly errors compared to ABySS; in fact, CISA and MIX produced more errors than SOAPdenovo.

Despite the fact that ABySS's assembly for *R. sphaeroides* had a higher number of misassembly errors than SOAPdenovo, none of the merged assemblies improved on the number of misassemblies compared to SOAPdenovo. Except for GAA, the number of misassembly errors produced by all tools were closer to the master (SOAPdenovo). As expected, tools that rely on the master assembly had a lower number of misassemblies than those that did not rank the inputs. With scaffolds as inputs, changes in NGA50 were negligible for all tools except for CISA. With contigs as inputs, GAM_NGS improved the contiguity by at most 11%, Metassembler and MIX increased it by 2%, and CISA dropped it by 85%. CISA also increased the number of contigs by 18%, and decreased genome and gene coverage by about 45%. GAM_NGS's assembly covered less than one quarter of the genome and about one fifth of the genes sequences, but its output had quality statistics similar to SOAPdenovo (with a 5% decrease in scaffolds). MIX and Metassembler decreased the number of scaffolds by 30% and 39%, respectively; otherwise, they maintained contiguity and coverage statistics within 1% of SOAPdenovo. GARM significantly improved the contiguity in terms of N50 but maintained the same NGA50 as SOAPdenovo. GARM decreased genome and gene coverage by 11%.

With contigs as inputs, GAM_NGS maintained the same genome and gene coverage as SOAPdenovo. MIX and Metassembler produced comparable results, namely (i) they both reduced the number of contigs by nearly one quarter, (ii) increased N50 by 10%, (iii) maintained the same genome coverage, and (iv) decreased gene coverage by less than 2%.

In the majority of the cases, experimental results obtained with ALLPATHS-LG (high N50) and SGA (low misassembly errors) on the *R. sphaeroides* genome (reported in Supplemental Table S5) followed similar patterns to the ones we observed in Supplemental Table S4. CISA increased the number of contigs, but decreased the contiguity, genome and gene coverage (although the reduction was far less this time). GAA followed the same general pattern mentioned earlier. GAM_NGS did not increase contiguity but rather maintained it close to that of the master assembly. Metassembler and MIX also did not increase contiguity, but they reduced the number of contigs, as well as genome and gene coverage. ZORRO worked for this experiment: it increased the number of contigs, decreased contiguity by 10%, but retained genome and gene coverage of ALLPATHS-LG. ZORRO's merged assembly is the only one that achieved a smaller number of misassembly errors than ALLPATHS-LG (but still higher that SGA).

With scaffolds as input assemblies, CISA again reduced the number of contigs and produced an assembly with low genome and gene coverage. GAM_NGS reduced the number of contigs slightly but retained the quality statistics of the master assembly. Observe in Supplemental Table S4 that GARM improved N50 by 57% although it retained NGA50 close to SOAPdenovo (the master assembly). Observe in Supplemental Table S5 that GARM maintained ALLPATHS-LG's contiguity statistics. In both experiments GARM decreased genome and gene coverage; on the

positive side, the consensus assembly had about 85% less scaffolds compared to the master.

Experimental results on the *Hg_chr14* with contigs as input assemblies (Supplemental Table S4), show that (i) GAM_NGS slightly improved contiguity, (ii) Metassembler maintained contiguity with fewer contigs, (iii) GAA crashed, (iv) number of misassemblies were closer to SOAPdenovo. With scaffolds as inputs, GARM drastically reduced the number of contigs, but also decreased the genome coverage by 7%. GAM_NGS and Metassembler produced assemblies with quality statistics close to SOAPdenovo except for a 26% decrease in the number of contigs for Metassembler.

3.2. **Reordering the inputs (GAGE)**

As mentioned above, some of the assembly reconciliation tools assume that the first input assembly is the master assembly, and should be "trusted" more (we call these tools *asymmetric*). The goal of this set of experiments is determine how the quality of the merged assembly depends on the specific order of the inputs.

To determine how the ranking affected the results, we repeated the same experiments reported in the previous section but switched the order of the inputs. A comparative analysis of the results in Supplemental Table S4 and Supplemental Table S6 prompts a few observations. First, we note that CISA, MIX, and GARM are *symmetric* (i.e., they do not require users to rank the inputs, see Main Text Table 1), hence they are expected to be unaffected by the reordering. Experimental results confirm that CISA and GARM are indeed unaffected. The reordering however affected MIX results, albeit only slightly.

For *S. aureus*, MIX's contiguity statistics (N50 and NGA50) and genome coverage were not affected by the reordering of the inputs. However, we observed (i) a 2% decrease in gene coverage, (ii) a small difference in the number of contigs (±1), and (iii) a small change in the number of misassemblies, although still higher than SOAPdenovo in both cases.

On the *R. sphaeroides* genome, all statistics remained unchanged except for the number of misassemblies that increased after reordering. In addition, with contigs as inputs we did not observe an increase in NGA50 after the reordering.

Despite the fact that GAA requires input ranking, the results for *S. aureus* and *R. sphaeroides* were similar. The output statistics of GAA followed the general pattern mentioned in the previous section. For *Hg_chr14*, GAA crashed in one ordering but not on the other. For all three genome, GAM_NGS and Metassembler produced consensus assemblies with quality statistics close to the master assembly.

Note that the merged assemblies have higher contiguity in Supplemental Table S4, in which the master has higher N50. In contrast, the number of misassemblies were lower in Supplemental Table S6 for both *S. aureus* and *Hg_chr14* in which the master had lower errors (with the exception of MIX). Merged assemblies for *R. sphaeroides* had higher contiguity and lower number of misassemblies, in which the master had higher N50 and lower number of misassemblies (see Supplemental Table S4).

3.3. **High-quality inputs (GAGE)**

In the third set of experiments we tested the ability of the reconciliation tools to merge two high quality assemblies. We selected two highly contiguous assemblies

(i.e., small number of contigs and scaffolds, high N50 values) and low number of misassembly errors. Supplemental Table S7 show the result of merging assemblies produced by ALLPATHS-LG as first input and either MSR-CA, SOAPdenovo, or CABOG as the second assembly.

Observe that for *S. aureus* with contigs as inputs, GAM_NGS produced an improved assembly that (i) had no misassemblies, (ii) was 66% more contiguous, and (iii) covered the same portions of the genome and the genes. The next best assembly was by Metassembler with a 107% increase in contiguity and a 51% decrease in the number of contigs, but it had a slight increase in the number of misassemblies compared to ALLPATHS-LG. MIX also improved the contiguity by 107% (N50), but due to the high number of misassemblies (higher than MSR-CA) the increase in contiguity dropped to 4% when aligned to the reference. MIX's gene coverage also dropped by 37%. CISA improved contiguity by 11%, and reduced the number of contigs by nearly a half, but it produced a number of errors higher than ALLPATHS-LG. CISA also decreased genome and gene coverage. ZORRO decreased contiguity by 30% and increased the number of contigs by 22%, although it maintained genome and gene coverage.

With scaffolds as inputs, ALLPATHS-LG has no misassemblies, a lower N50 than MSR-CA but higher NGA50. In general, asymmetric tools produced a lower number of misassemblies and decreased the N50. For instance, GAM_NGS maintained quality statistics of ALLPATHS-LG. Although ZORRO is asymmetric it decreased contiguity by more than 90%. On the other hand, symmetric tools had a higher number of misassemblies. GARM achieved the highest increase of NGA50 (16%).

The contiguity of the merged assemblies improved $11\% - 108\%$ with the exception of ZORRO, which decreased the contiguity by 30%. GARM increased contiguity the most (108%) at the expense of (i) an additional 12% duplication rate, (ii) a number of misassemblies close to MSR-CA, and (iii) a 10% decrease in gene coverage. MIX introduced no misassemblies, but covered only 25% of the genome and gene sequences. Notably, both GAM_NGS and Metassembler (i) improved contiguity by 66.5%, (ii) reduced the number of contigs, (iii) introduced no misassemblies, (iv) and maintained gene coverage. These are two rare examples in which we observed an unquestionable improvement in the merged assembly.

On the *R. sphaeroides* genome, the two input assemblies had almost the same number of misassemblies but the assembly produced by SOAPdenovo was much less fragmented. Only MIX, Metassembler and GARM increased N50 by 37%, 43%, and 69%, respectively (with only Metassembler increasing NGA50 significantly). All other tools decreased the contiguity. In terms of correctness, ZORRO and CISA (using scaffolds as inputs) reduced the number of misassemblies but also decreased the contiguity by 99% and 60%, respectively. Other tools produced merged assemblies with a number of misassemblies not better than the inputs.

GARM improved the contiguity by 38% while CISA increased it by less than 2%. GARM, CISA, and MIX reduced the number of contigs by $48\%, 51\%$, and 60%, respectively, but also decreased genome and gene coverage. MIX is the only tool that reduced the number of misassemblies, but again its assembly only covered about half of the genome. None of the tools improved both contiguity and the number of misassemblies.

In *Hg_chr14*, GAA decreased the contiguity by 8%, but it improved the NGA50 by 76%, and increased the gene coverage by 13%. Nevertheless, it had a 198% inflation rate and produced a number of misassemblies equal to the sum of the number of misassemblies in the two inputs. GAM_NGS reduced the number of contigs by 10%, improved the contiguity (39% increase in N50, 28% increase in NGA50), slightly reduced the number of misassemblies, but decreased the gene coverage by 11%. Metassembler produced quality statistics that are very close to ALLPATHS-LG.

With scaffolds as inputs, GAM_NGS and Metassembler maintained similar quality statistics to ALLPATHS-LG, with the exception of the number of contigs (Metassembler decreased it by 33%) and gene coverage (GAM_NGS and Metassembler decreased by 18% and 51%, respectively). GARM improved N50 but decreased NGA50 by 9%. It also increased the number of misassemblies and decreased genome and gene coverage.

GARM improved the contiguity by 128% and reduced the number of contigs in half at the cost of 14% inflation and about 41% increase in the number of misassemblies. GAA and GAM_NGS improved the contiguity by 76% and 28%, but only GAA increased the gene coverage.

3.4. Highly-fragmented inputs (GAGE)

The goal of this set of experiments was to evaluate the performance of assembly reconciliation tools when provided with two highly fragmented input assemblies. Input assemblies were selected to have a high percentage of contigs shorter than 200 bps, a high number of contigs and scaffolds, and low N50.

Supplemental Table S8 shows the results of merging ABySS assembly and SGA assembly. Observe that when we used contigs as inputs, ABySS had a higher contiguity than SGA (except in *Hg_chr14*). The opposite, however, was observed when scaffolds were provided in input. In *S. aureus* and *R. sphaeroides* with contigs as inputs, all tools increased N50 except for GAA. In terms of NGA50, only asymmetric tools maintained or improved over NGA50 of the better input assembly (in *S. aureus* we observed up to 8% increase, and up to 17% in *R. sphaeroides*). However, in *Hg_chr14*(with contigs as inputs) only GAM_NGS improved the N50. In terms of NGA50, GAA produced a 123% increase over SGA, while GAM_NGS did not improve it over SGA, but it increased it 33% over ABySS.

With scaffolds as inputs, we observed a decrease in N50 except for MIX and GARM (when SGA inputs are scaffolds). MIX, GARM, and CISA are symmetric tools, hence they are expected to perform better than other tools when the non-master input has better quality. CISA, however, produced inferior results with scaffolds as inputs in most experiments. It turns out that CISA with default parameters break scaffolds into contigs when a scaffold contains more than ten consecutive occurrences of Ns. MIX and GARM enhanced or maintained N50 of SGA. In terms of NGA50, MIX maintained it, while GARM slightly decreased it compared to SGA (yet still higher than ABySS). The number of contigs decreased although it remained relatively high in the majority of the cases. CISA had more than 80% decrease in the number of contigs with scaffolds as inputs, but the genome coverage was poor. GARM reduced the number of contigs by $74 - 91\%$, regardless of the genome coverage.

3.5. De Bruijn vs. string graph assembly (GAGE)

Here we tested the effect of merging assemblies generated using different assembly strategies. Specifically, we merged an assembly generated by an assembler that uses a de Bruijn graphs with an assembly produced by an assembler based on the string graph. Supplemental Table S5 shows the result of merging an assembly produced by ALLPATHS-LG (based on the de Bruijn graph) with an assembly produced by SGA (based on the string graph). Overall, GAM_NGS, Metassembler, and MIX maintained similar assembly statistics as ALLPATHS-LG.

Note that *S. aureus* input assemblies (as contigs) had only one misassembly. The merged assemblies also have one misassembly, with the exception of GAA (two) and ZORRO (none). ZORRO corrected the assembly error without affecting N50 but at the price of a 17% increase in the number of contigs. CISA also increased the number of contigs, decreased NGA50 by 49%, and decreased the gene coverage by 15%. With scaffolds as inputs, ALLPATHS-LG's assembly has no assembly errors. In fact, observe that all merged assemblies did not have any misassemblies. GARM produced only 3 scaffolds and increased N50 by 31% but kept NGA50 close to ALLPATHS-LG, while decreasing less than 6% of genome and gene coverage. CISA covered less than 40% of the genome, while ZORRO decreased the contiguity by 99%.

On *R. sphaeroides* with contigs as inputs, CISA and ZORRO decreased the contiguity by 34% and 10%, respectively. CISA decreased genome and gene coverage by 8%, while ZORRO maintained ALLPATHS-LG's coverage. GAM_NGS and Metassembler slightly reduced the number of contigs; otherwise they maintained ALLPATHS-LG's quality statistics. All tools produced a relatively high number of misassemblies (similar to ALLPATHS-LG). With scaffolds as inputs, CISA, ZORRO, and GARM's assembly statistics followed the same of statistics of *S. aureus*. All assemblies, with the exception of CISA and ZORRO, had a number of misassemblies closer to ALLPATHS-LG. CISA again covered less than one fifth of the genome and ZORRO decreased the contiguity by 99%. GARM produced only four contigs but decreased the genome coverage by less than 5%. GAM_NGS, Metassembler, and MIX produced consensus assemblies with quality statistics comparable to ALLPATHS-LG.

In *Hg_chr14* (with contigs as inputs) GAM_NGS and Metassembler reduced the number of contigs by 4% and 2%, respectively. GAM_NGS increased NGA50 by 2%. With scaffolds as inputs, GAM_NGS and Metassembler maintained assembly statistics close to ALLPATHS-LG except for the fact that Metassembler reduced the number of contigs by 21%. GARM reduced the number of contigs by 83%, maintained genome and gene coverage but increased the number of misassemblies by 9% (compared to ALLPATHS-LG) and decreased NGA50 by 9%.

GARM increased contiguity by 58%, while other tools improved it by less than 3%. GAM_NGS and Metassembler produced about the same number of misassembly errors as the higher of the two inputs. GARM improved NGA50 the most, but also increased the number of misassemblies by 42% and had 31% inflation rate.

3.6. Multiple inputs (GAGE)

In this set of experiments we tested the ability of the tools to merge more than two assemblies. When an assembly reconciliation tool allowed no more than two assem-

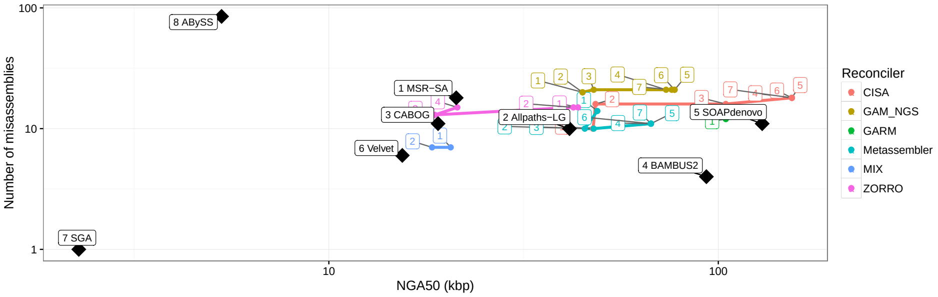

Supplemental Figure S1: Experimental results on merging more than two assemblies (as contigs) ordered by the FRCurve score (*R. sphaeroides*, genome size 4,603,060 bp). The Figure reports on quality of merged assembly compared to the input assemblies. Tools were ran using default parameters, unless otherwise noted

blies in input (see Main Text Table 1 for a list), we merged them in an iterative fashion. For instance, to merge three assemblies, we first merged two assemblies, then merged the result to the third assemblies. Metassembler uses a similar strategy: when the user provides multiple assemblies the tool iteratively performs pairwise reconciliation, where the output of one iteration is the input of the next. The ordering of the input assemblies was chosen based on *feature response* curve (FR curve), which is an assembly quality metric proposed in [1]. The FR curve represents the dependency between contigs that contains no more than τ features and the corresponding genome coverage. The x-axis represents τ and the y-axis represent genome coverage: the "steeper" is the curve, the better is the assembly. We used the FR curves in [2] to determine the merging order of the GAGE assemblies, starting with the assemblies with highest quality. Results for an alternative ordering is discussed in the next section. For tools that allowed to merge more than two assemblies (e.g., CISA and MIX), the merging was done in one step from the original assemblies. Here we were interested in measuring the contiguity and correctness of the resulting assemblies as the number of input assemblies increases.

Supplemental Tables S9, S10, S11 and Supplemental Figures S1 and S2, show the experimental results for *S. aureus*, *R. sphaeroides* and *Hg_chr14*, respectively, when inputs are contigs. First observe that in several cases, the process of iterative merging did not complete.

On *S. aureus* and *R. sphaeroides*, CISA generally improved the contiguity and decreased the number of contigs as the number of merged assemblies increased. The number of errors and the percentage of genome covered fluctuated over the iterations. As the number of merged assemblies increased, CISA increased the duplication rate and decreased the percentage of covered genes. GAA did not produce assembly files for the first iteration. Although GAA did not work for this particular ordering it did produce results for the alternative ordering reported in the next section.

In *S. aureus* and *R. sphaeroides*, GAM_NGS's contiguity improved over successive iterations, but the number of misassemblies errors did not decrease (it stayed close to the first master input in all iterations). On the positive side, (i) the number of contigs was relatively small and (ii) the percentage of genome covered was relatively high,

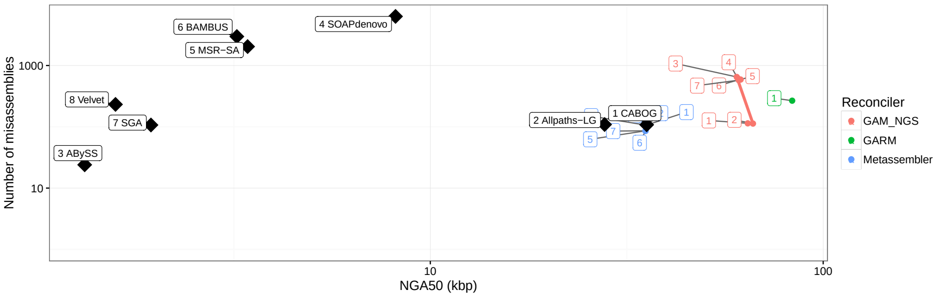

Supplemental Figure S2: Experimental results on merging more than two assemblies (as contigs) ordered by the FRCurve score (*Hg_chr14*, genome size 107,349,540 bp). The Figure reports on quality of merged assembly compared to the input assemblies. Tools were ran using default parameters, unless otherwise noted

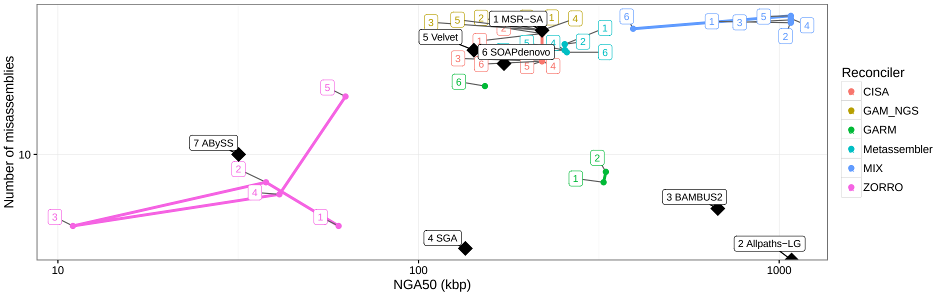

Supplemental Figure S3: Experimental results on merging more than two assemblies (as scaffolds) ordered by the FRCurve score (*S. aureus*, genome size 2,903,081 bp). The Figure reports on quality of merged assembly compared to the input assemblies. Tools were ran using default parameters, unless otherwise noted

and (iii) gene coverage was relatively high, although slightly lower than the best gene coverage in the input assemblies. In contrast, the percentage of gene coverage decreased for *Hg_chr14*. Although the genome coverage and contiguity were high, the number of misassemblies was also relatively high. GAM_NGS increased NGA50 by at least 70% compared to CABOG.

In *S. aureus*, Metassembler's contiguity improved and the number of contigs decreased over successive iterations, but the number of misassemblies also increased. Metassembler maintained high genome and gene coverage, although slightly lower than the best gene coverage in the input assemblies. In *R. sphaeroides*, Metassembler's assembly did not improve after the forth iteration. Note that NGA50 was lower than BAMBUS2 and SOAPdenovo. Metassembler's assembly had low genome and gene coverage and number of misassemblies was about the average of the inputs. In *Hg_chr14*, the number of contigs and misassembly errors were low and decreased over successive iterations. Contiguity, genome and gene coverage were high, but slightly decreased over successive iterations.

MIX maintained a low number of misassemblies in most iterations but suffered from low genome and gene coverage. Also, NGA50 was relatively poor. Since the genome coverage in most iterations was less than 50% of the reference, no NGA50 was reported for those iteration. On the *S. aureus* genome, the coverage was less

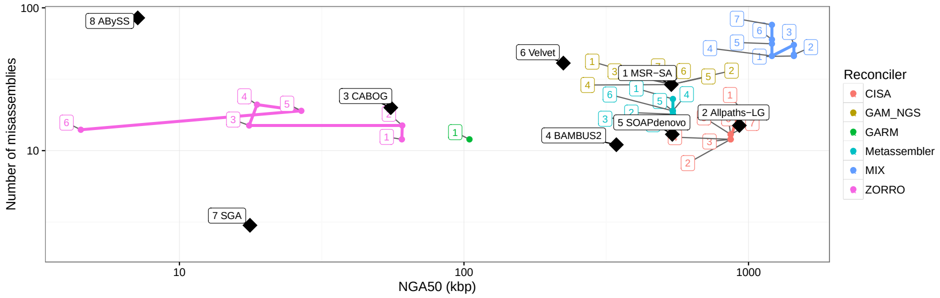

Supplemental Figure S4: Experimental results on merging more than two assemblies (as scaffolds) ordered by the FRCurve score (*R. sphaeroides*, genome size 4,603,060 bp). The Figure reports on quality of merged assembly compared to the input assemblies. Tools were ran using default parameters, unless otherwise noted

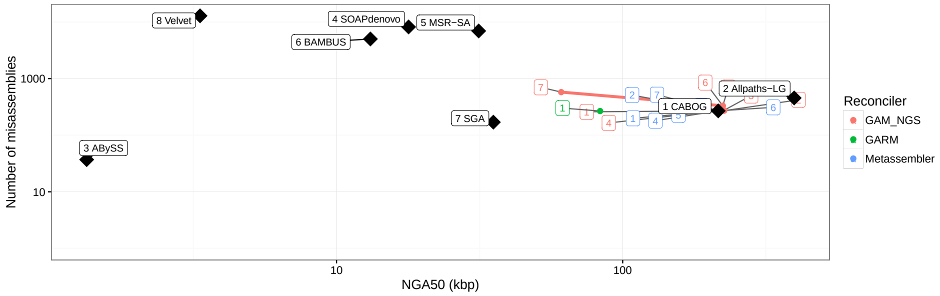

Supplemental Figure S5: Experimental results on merging more than two assemblies (as scaffolds) ordered by the FRCurve score (*Hg_chr14*, genome size: 107,349,540). The Figure reports on quality of merged assembly compared to the input assemblies. Tools were ran using default parameters, unless otherwise noted

than 50% in all iterations but it steadily improved with increasing number of inputs. On *R. sphaeroides*, the genome coverage was below 50% with four or more inputs.

ZORRO frequently failed to produce results. When it worked, it increased genome and gene coverage. Contiguity usually started high, then fluctuated over iterations. ZORRO produced relatively high number of contigs and misassemblies (somewhat in between the values of the inputs).

We repeated the same experiment but with scaffolds as inputs. Results are reported in Supplemental Tables S12, S13, and S14 and Supplemental Figures S3, S4, and S5. CISA's results show that after a certain number of input assemblies, increasing the number of inputs did not affect the results significantly. From that point forward, it generally improved the contiguity and reduced the number of contigs as the number of merged assemblies increased, at the cost of decreased genome and gene coverage and about 25% inflation rate. The number of misassemblies were with the range of input assemblies. CISA reached stability with four inputs on *S. aureus* and three inputs on *R. sphaeroides*).

MIX maintained a low number of contigs albeit this number fluctuated in *R. sphaeroides* with increasing number of inputs. MIX also produced a high duplication ratio. On *S. aureus*, MIX produced a high number of misassemblies which generally increased as the number of inputs increased. It maintained high genome

coverage but gene coverage was poor in comparison to the inputs. It also maintained high contiguity except for the last iteration. On *R. sphaeroides*, the number of misassemblies were also relatively high but it fluctuated as the number of inputs increased. Genome coverage increased steadily but gene coverage decreased. It also maintained high contiguity, achieving the best NGA50 for less than five inputs.

ZORRO produced a high number of contigs and a low number of misassemblies on *S. aureus* and *R. sphaeroides*. It maintained a high genome coverage but it slightly decreased gene coverage. Contiguity was poor and generally decreased over successive iterations.

GAM_NGS maintained results very close to the first input throughout all iterations on *S. aureus*, *R. sphaeroides*, and *Hg_chr14*. In the latter genome, GAM_NGS contiguity generally improved in successive iterations but so did the number of misassemblies.

Metassembler maintained similar quality statistics to CABOG on *Hg_chr14*, although the number of contigs slightly decreased over successive iteration. On *R. sphaeroides*, Metassembler also maintained CABOG's quality statistics with a slight decrease of (i) number of contigs, (ii) number of misassemblies, (iii) genome and gene coverage, and (iv) contiguity, as the number of iteration increased. On *S. aureus*, Metassembler also maintained quality statistics close but not identical to MSR-CA. In general, Metassembler produced a small number of contigs. Also, as the number of inputs increased, the number of misassemblies slightly decreased and the contiguity slightly improved.

3.7. **Multiple inputs (alternative ordering)**

In this set of experiments we tested the ability of the tools to merge more than two assemblies on an alternative ordering to the FR curves used in the main Text. Recall that when an assembly reconciliation tool allowed no more than two assemblies in input (see Table 1 in the main text for a list), we merged them in an iterative fashion starting from the most contiguous assemblies (see main Text for more details)

Supplemental Tables S15, S16, S17, and S18 show the experimental results for *S. aureus*, *R. sphaeroides* (two tables) and *Hg_chr14*, respectively on this alternative ordering. Supplemental Figures S6 – S8 summarize the results with respect to contiguity and correctness. First observe that similar to what we observed for the ordering based on FR curves, in many instances the process of iterative merging did not complete.

On *S. aureus* and *R. sphaeroides*, CISA generally increased the contiguity and decreased the number of contigs as the number of merged assemblies increased. The number of errors and the percentage of genome covered fluctuated over the iterations. While the percentage of covered genes peaked with three input assemblies, CISA increased the duplication rate as the number of merged assemblies increased. GAA instead increased contiguity, number of errors, and duplication rate and the percentage of covered genome fraction, as the number of merged assemblies increased.

In *S. aureus*, *R. sphaeroides*, and *Hg_chr14*, GAA produced a monotonic increase in duplication rate at successive iterations, while misassemblies seemed to be the union of those present in the input assemblies. GAA's contiguity did not increase

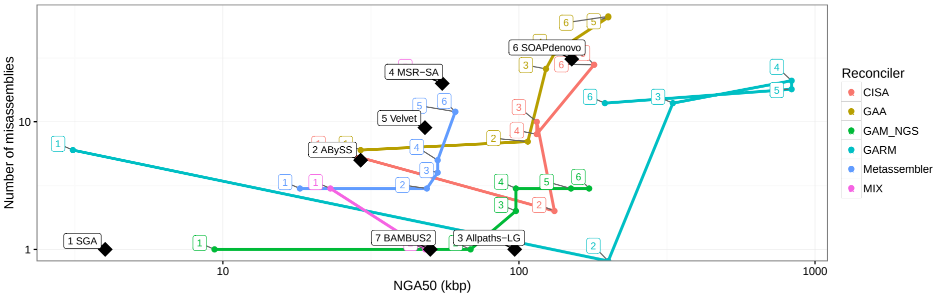

Supplemental Figure S6: Experimental results on merging more than two assemblies (as contigs) – alternative ordering (*S. aureus*, genome size 2,903,081 bp). The Figure reports on quality of merged assembly compared to the input assemblies. Tools were ran using default parameters, unless otherwise noted

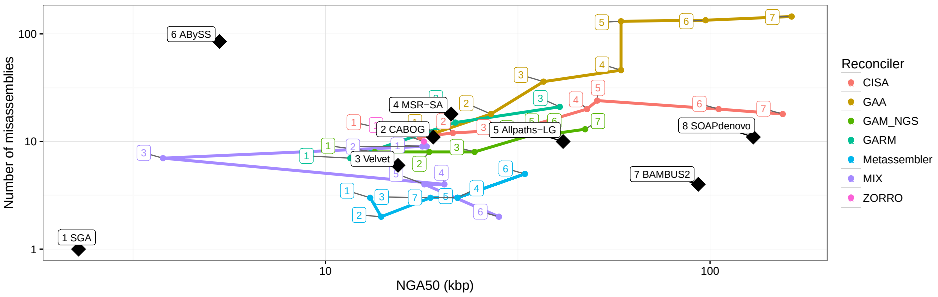

Supplemental Figure S7: Experimental results on merging more than two assemblies (as contigs) – alternative ordering (*R. sphaeroides*, genome size 4,603,060 bp). The Figure reports on quality of merged assembly compared to the input assemblies. Tools were ran using default parameters, unless otherwise noted

over successive iterations, but the genome coverage was relatively high, while gene coverage which was very low in both *S. aureus* and *R. sphaeroides*.

GAM_NGS's contiguity increased over successive iterations, but the number of misassemblies did not decrease. On the positive side, the number of misassemblies was small and the percentage of genome covered was high. In *S. aureus* and *R. sphaeroides*, gene coverage was high, although slightly lower than the best gene coverage in the input assemblies. In contrast, the percentage of gene coverage decreased for *Hg_chr14*.

GARM increased the contiguity over successive iterations but also inflated the resulting assembly. The number of misassemblies and the genome/gene coverage fluctuated. The percentage of gene coverage decreased in *Hg_chr14*. In *R. sphaeroides*, GARM crashed after the third iteration. Note that in the second iteration of *S. aureus* only 26 contigs covered nearly 93% of the genome with 91% gene coverage, no misassemblies, and no inflation. In *S. aureus*, Metassembler maintained a low error rate and NGA50 (with the exception of *Hg_chr14*) over successive iterations (although NGA50 was consistently low). In *Hg_chr14*, NGA50 was low and also decreasing over iterations. In *R. sphaeroides*, genome and gene coverage for Metassembler was low with respect to input assemblies.

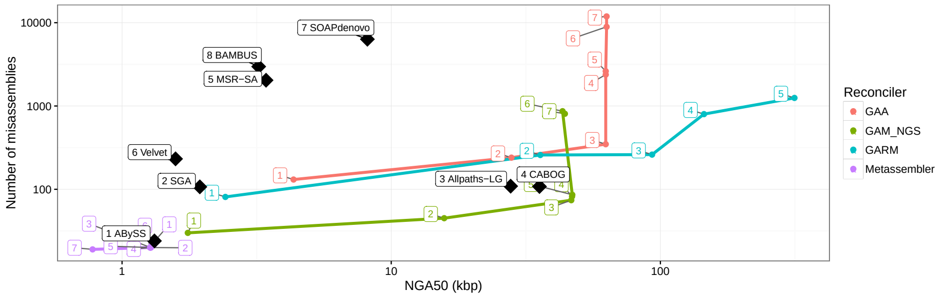

Supplemental Figure S8: Experimental results on merging more than two assemblies (as contigs) – alternative ordering (*Hg_chr14*, genome size 107,349,540 bp). The Figure reports on quality of merged assembly compared to the input assemblies. Tools were ran using default parameters, unless otherwise noted

MIX maintained a low number of misassemblies in most iterations but suffered from low genome and gene coverage. Its NGA50 fluctuated over successive iteration, but it was relatively poor. Since the genome coverage in some iterations is less than 50% of the reference, no NGA50 was reported for those iteration.

ZORRO frequently failed to produce results. When it worked, it increased the percentage of genome coverage and gene coverage and it did not increased duplication.

Supplementary Note 4: Time and Space Analysis

As said, all experiments were performed on a Linux Ubuntu 12.10 server with a 20 cores Intel Xeon CPU E5-2690v2 3GHz and 512GB of RAM. Multithreading was used when available.

First, we measured the usage of computational resources to merge two input assemblies. Graphs in Supplemental Figure S9 illustrate the average (wall clock) run time, the average percentage of processor utilization (where 100% indicates full utilization of one core), and the average memory usage required by each tool to perform each experiments on the four genomes. The average are over all the tested pairs of GAGE assemblies for that genome. Error bars indicate the minimum and maximum.

Second, we measured the usage of computational resources as a function of the number of input assemblies using CISA and MIX, which are the only tools that can merge more than two input assemblies. Graphs in Supplemental Figure S10 shows the (wall clock) run time, processor utilization (where 100% indicates full utilization of one core), and memory usage as the number of input assemblies increases.

Supplementary Note 5: Large genomes (GAGE)

To test the ability of these tools to scale to large eukaryotic genomes, we used GAGE's assemblies for *Bombus impatiens*. We selected the two input assemblies where most of the tools were able to complete. A high quality reference genome is unavailable for *Bombus impatiens*, so the statistics we reported were produced by the GAGE script. In addition to the usual assembly statistics, GAGE computes the *e-size*, which is the expected size of a contig (or scaffold). The e-size if computed

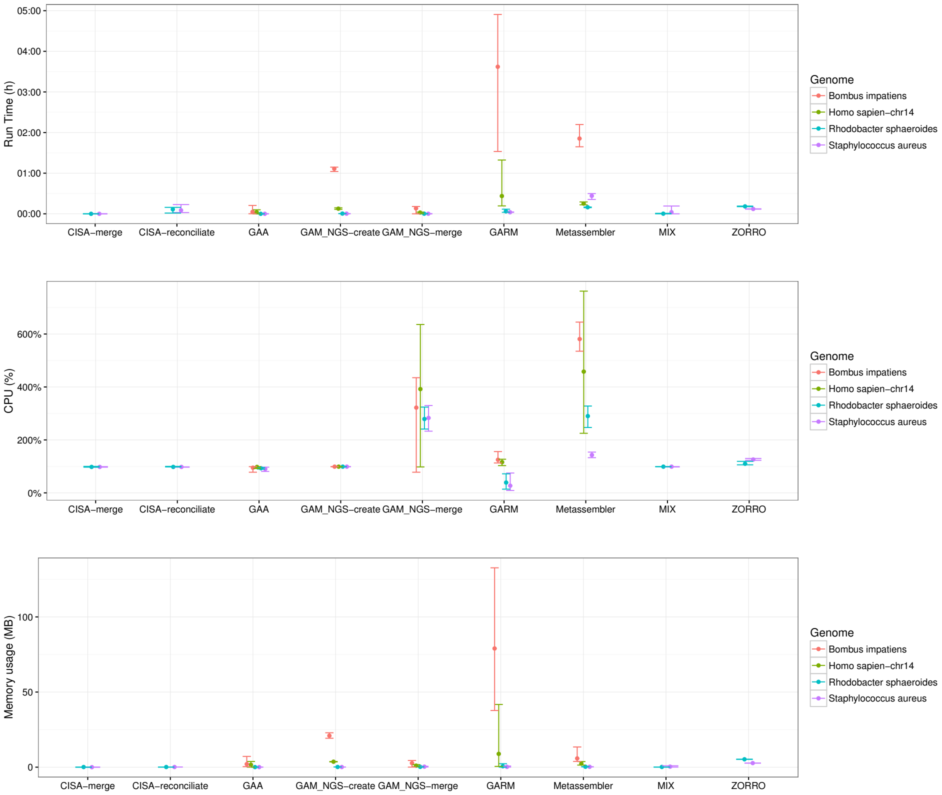

Supplemental Figure S9: Average (wall clock) run time, average percentage of processor utilization (where 100% indicates full utilization of one core), and average memory usage required by each tool to perform each experiments on the four genomes; averages are over all the tested pairs of GAGE assemblies for that genome; error bars indicate the minimum and maximum

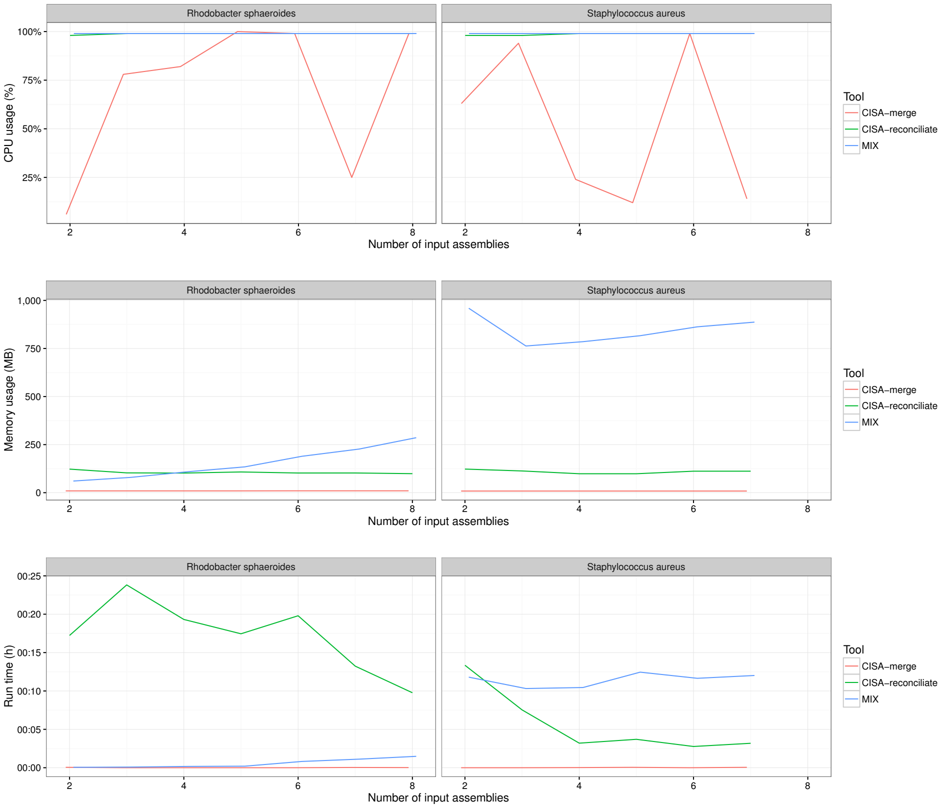

Supplemental Figure S10: Wall clock run time, processor utilization (where 100% indicates full utilization of one core), and memory usage as the number of input assemblies increases (for CISA and MIX)

as $\sum_c L_c^2/G$, where the sum is over all contigs c, G is the expected genome length and L_c is the length of contig c [3].

Results are reported in Supplemental Table S19, in which only contigs and scaffolds of 500 bp or longer were considered. Observe that GARM reduced the number of contigs, increased N50 and the e-size for all experiments. GAM_NGS did not work for one of the experiments. In the others, it decreased the number of contigs in all but one experiment. GAM_NGS always improved N50, and increased the e-size in all but one experiment. GAA did not work for two of the experiments. When it worked, it did not reduce the number of contigs, but it increased both N50 and the e-size. Lastly, Metassembler decreased N50 and the e-size in three out of four experiments. Metassembler reduced the number of contigs in half of the experiments.

Supplementary Note 6: Limitations

- MIX and CISA: we did not run these two tools on the *Hg_chr14* dataset because they were designed for bacteria-sized genome and they would not handle such a large input
- GARM: while GARM's manual claims that the tool can accept two contigs, two scaffolds, or contig/scaffold combination as an input, we were only successful to run the tool using one contig and one scaffold; in most cases, running with two contigs produced an empty FASTA file, while using two scaffolds produced FASTA files with all nucleotides set to N

Supplementary Note 7: Usage of reads

Some of the tools can take advantage of the raw reads, in addition to the input assemblies. For GAA, while the paper mentions using paired-end reads for error correction, there is no option to provide them. Therefore, we didn't use them for GAA. We used reads in these cases:

- GAM_NGS: we used paired-end reads with a 155-180 bp insert (Library 1 in GAGE)
- Metassembler: for bacterial genomes we used the available short-jump library (insert size of 3500 bp); for *Hg_chr14* we used the available long-jump library (insert size is approximately 35 kbp), and for *Bombus impatiens* we used the available short-jump library 2 (insert size is approximately 8 kbp)
- ZORRO: we used paired-end reads with a 155-180 bp insert (Library 1 in GAGE)

Author details

References

1. Narzisi, G., Mishra, B.: Comparing de novo genome assembly: The long and short of it. PLoS ONE **6**(4), 1–14 (2011)
2. Vicedomini, R., Vezzi, F., Scalabrin, S., Arvestad, L., Policriti, A.: GAM-NGS: genomic assemblies merger for next generation sequencing. BMC Bioinformatics **14**(Suppl 7), 6 (2013)
3. Salzberg, S.L., Phillippy, A.M., Zimin, A., Puiu, D., Magoc, T., Koren, S., Treangen, T.J., Schatz, M.C., Delcher, A.L., Roberts, M., Marais, G., Pop, M., Yorke, J.A.: GAGE: A critical evaluation of genome assemblies and assembly algorithms. Genome Research **22**(3), 557–567 (2012)

Supplemental Table S1: Experimental results on parameter tuning (*S. aureus*, genome size 2,872,915 bp). The table reports on quality of merged assembly compared to the two input assemblies. Notes: Reported statistics are for contigs; the number of mismatches/indels/Ns are per 100 Kbps

Reconciliation Tool	Contigs (#)	Largest (bp)	Size (bp)	N50 (bp)	Misassembly (#)	Misassembly Length (bp)	Mismatches (#)	Indels (#)	N's (#)	Genome covered (%)	Duplication ratio	NGA50 (bp)	Genes (%)
S. aureus (genome size 2,872,915 bp)													
ALLPATHS-LG	59	234,488	2,869,581	96,740	1	89,634	1.57	0.73	1.50	98.83	1.00	96,740	96
SGA	985	16,870	2,748,664	4178	1	2431	1.02	0.11	0.00	94.44	1.00	4005	82
CISA	41	234,488	1,868,640	86,588	2	170,232	2.30	0.96	1.34	64.53	1.00	46,021	63
CISA	41	234,488	1,868,640	86,588	2	170,232	2.30	0.96	1.34	64.53	1.00	46,021	63
CISA	66	127,888	2,199,244	62,398	1	80,598	1.45	0.68	0.77	75.91	1.00	42,963	74
CISA	4	461,617	1,286,214	286,534	0	0	0.98	8.46	7240.24	38.69	1.15	NA	35
CISA	10	924,846	1,860,009	614,593	1	924,846	3.93	2.75	520.70	63.91	1.01	190,401	64
GAM_NGS	59	234,488	2,869,581	96,740	1	89,634	1.57	0.73	1.50	98.83	1.00	96,740	96
GAM_NGS	59	234,488	2,869,725	96,740	1	89,634	1.57	0.73	1.50	98.83	1.00	96,740	96
GAM_NGS	59	234,488	2,869,696	96,740	1	89,634	1.57	0.73	1.50	98.83	1.00	96,740	96
GAM_NGS	59	234,488	2,869,725	96,740	1	89,634	1.57	0.73	1.50	98.83	1.00	96,740	96
GAM_NGS	59	234,488	2,869,581	96,740	1	89,634	1.57	0.73	1.50	98.83	1.00	96,740	96
GARM	54	234,577	3,716,040	101,091	1	89,633	1.70	1.49	0.13	97.25	1.32	137,450	64
GARM	54	234,577	3,716,505	101,091	1	89,633	1.88	1.45	0.13	97.25	1.32	137,450	64
GARM	54	234,581	3,715,089	101,091	1	89,633	1.95	1.35	0.13	97.21	1.32	137,450	64
GARM	54	234,581	3,715,092	101,091	1	89,633	1.84	1.35	0.13	97.21	1.32	137,450	64
GARM	54	234,581	3,715,110	101,091	1	89,633	2.27	1.35	0.13	97.21	1.32	137,450	64
MIX	53	234,488	2,739,016	97,697	1	89,634	1.46	0.69	1.50	94.34	1.00	96,740	92
MIX	56	234,488	2,760,919	97,697	1	89,634	1.45	0.69	1.49	95.09	1.00	96,740	92
MIX	58	234,488	2,821,277	97,697	1	89,634	1.60	0.74	1.52	97.16	1.00	96,740	94
MIX	59	234,488	2,869,581	96,740	1	89,634	1.57	0.73	1.50	98.83	1.00	96,740	96
MIX	59	234,488	2,869,581	96,740	1	89,634	1.57	0.73	1.50	98.83	1.00	96,740	96
Metassembler	59	234,488	2,869,581	96,740	1	89,634	1.57	0.73	1.50	98.83	1.00	96,740	96
Metassembler	59	234,488	2,869,581	96,740	1	89,634	1.57	0.73	1.50	98.83	1.00	96,740	96
Metassembler	59	234,488	2,869,581	96,740	1	89,634	1.57	0.73	1.50	98.83	1.00	96,740	96
Metassembler	59	234,488	2,869,581	96,740	1	89,634	1.57	0.73	1.50	98.83	1.00	96,740	96
Metassembler	59	234,488	2,869,581	96,740	1	89,634	1.57	0.73	1.50	98.83	1.00	96,740	96
ZORRO	66	234,548	2,873,037	96,674	0	0	1.88	0.66	0.80	98.91	1.00	96,674	96
ZORRO	66	234,548	2,873,101	96,674	0	0	1.85	0.66	0.77	98.91	1.00	96,674	96
ZORRO	67	234,548	2,875,958	96,674	0	0	1.81	0.66	0.80	98.91	1.00	96,674	96
ZORRO	69	234,548	2,873,546	96,657	0	0	1.99	0.59	0.90	98.90	1.00	96,657	97
ZORRO	69	234,548	2,877,597	96,674	0	0	1.92	0.63	1.22	98.91	1.00	96,674	96

Supplemental Table S2: Experimental results on parameter tuning (*R. sphaeroides*, genome size 4,603,060 bp). The table reports on quality of merged assembly compared to the two input assemblies. Notes: Statistics reported are for contigs; the number of mismatches/indels/Ns are per 100 Kbps;

Reconciliation Tool	Contigs (#)	Largest (bp)	Size (bp)	N50 (bp)	Misassembly (#)	Misassembly Length (bp)	Mismatches (#)	Indels (#)	N's (#)	Genome covered (%)	Duplication ratio	NGA50 (bp)	Genes (%)
ALLPATHS-LG	203	106,467	4,587,354	42,455	10	404,185	6.33	4.77	2.79	99.20	1.00	41,487	93
SGA	2173	29,520	4,188,432	2530	1	4048	5.70	2.47	0.00	90.70	1.00	2280	78
CISA	173	106,467	3,912,727	42,455	5	260,432	7.16	4.80	2.68	84.60	1.00	35,058	80
CISA	173	106,467	3,912,727	42,455	5	260,432	7.16	4.80	2.68	84.60	1.00	35,058	80
CISA	173	106,467	3,912,727	42,455	5	260,432	7.16	4.80	2.68	84.60	1.00	35,058	80
CISA	21	913,837	1,328,158	913,837	6	1,228,349	8.65	5.90	714.30	84.60	1.02	NA	28
CISA	21	913,837	1,328,158	913,837	6	1,228,349	8.65	5.90	714.30	84.60	1.02	NA	28
GAA	2348	106,467	8,730,704	10,451	11	408,233	6.74	4.79	1.47	99.32	1.91	41,487	25
GAA	2348	106,467	8,730,704	10,451	11	408,233	6.74	4.79	1.47	99.32	1.91	41,487	28
GAA	2348	106,467	8,730,704	10,451	11	408,233	6.74	4.79	1.47	99.32	1.91	41,487	29
GAA	2348	106,467	8,730,704	10,451	11	408,233	6.74	4.79	1.47	99.32	1.91	41,487	29
GAA	2348	106,467	8,730,704	10,451	11	408,233	6.74	4.79	1.47	99.32	1.91	41,487	29
GAM_NGS	201	106,467	4,588,158	42,455	10	408,636	6.61	4.77	2.79	99.22	1.00	41,487	93
GAM_NGS	201	106,467	4,588,158	42,455	10	408,636	6.61	4.77	2.79	99.22	1.00	41,487	93
GAM_NGS	202	106,467	4,588,119	42,455	10	404,185	6.61	4.77	2.79	99.22	1.00	41,487	93
GAM_NGS	202	106,467	4,588,119	42,455	10	404,185	6.61	4.77	2.79	99.22	1.00	41,487	93
GAM_NGS	203	106,467	4,711,689	42,802	11	515,103	6.61	4.77	2.74	99.22	1.03	42,802	91
GARM	197	97,474	5,615,046	47,696	3	80,924	9.52	6.42	0.00	86.23	1.42	53,525	0
GARM	199	97,480	5,518,631	47,676	3	80,924	9.55	6.40	0.00	86.26	1.39	52,061	52
GARM	201	97,474	5,616,723	47,696	3	80,924	9.47	6.40	0.00	86.26	1.42	53,525	52
GARM	201	97,476	5,616,676	47,696	3	80,924	9.55	6.30	0.00	86.26	1.42	53,525	52
GARM	205	97,456	5,749,214	47,696	4	142,754	8.36	6.17	0.00	86.28	1.45	59,372	50
MIX	194	106,467	4,351,909	42,455	7	353,063	5.28	4.62	2.73	94.14	1.00	40,555	88
MIX	196	106,467	4,423,168	42,455	7	353,063	5.20	4.59	2.74	95.69	1.00	41,487	90
MIX	197	106,467	4,466,781	42,455	7	353,063	5.33	4.63	2.71	96.64	1.00	41,487	91
MIX	199	106,467	4,481,045	42,455	7	353,063	5.13	4.68	2.70	96.95	1.00	41,487	91
MIX	201	106,467	4,535,084	42,444	7	353,063	5.25	4.72	2.67	98.12	1.00	41,487	92
Metassembler	200	106,467	4,587,010	42,455	10	408,636	6.61	4.77	2.79	99.22	1.00	41,487	93
Metassembler	200	106,467	4,587,010	42,455	10	408,636	6.61	4.77	2.79	99.22	1.00	41,487	93
Metassembler	200	106,467	4,587,010	42,455	10	408,636	6.61	4.77	2.79	99.22	1.00	41,487	93
Metassembler	200	106,467	4,587,010	42,455	10	408,636	6.61	4.77	2.79	99.22	1.00	41,487	93
Metassembler	200	106,467	4,587,010	42,455	10	408,636	6.61	4.77	2.79	99.22	1.00	41,487	93
ZORRO	222	106,470	4,609,096	41,918	9	388,589	7.09	4.81	1.41	99.28	1.01	41,235	93
ZORRO	224	105,319	4,616,066	42,444	8	296,208	7.16	4.84	1.54	99.28	1.01	41,487	92
ZORRO	231	105,269	4,594,505	37,312	8	215,802	6.98	4.70	1.41	99.30	1.00	37,195	93
ZORRO	231	105,269	4,595,263	37,312	8	215,799	6.94	4.70	1.46	99.30	1.00	37,195	93
ZORRO	231	105,315	4,596,344	37,312	8	215,902	6.96	4.68	1.46	99.29	1.01	37,195	93

Supplemental Table S3: Experimental results on parameter tuning (*Hg_chr14*, genome size 88,289,540 bp). The table reports on quality of merged assembly compared to the two input assemblies. Notes: Reported statistics are for contigs; the number of mismatches/indels/Ns are per 100 Kbps;

Reconciliation Tool	Contigs (#)	Largest (bp)	Size (bp)	N50 (bp)	Misassembly (#)	Misassembly Length (bp)	Mismatches (#)	Indels (#)	N's (#)	Genome covered (%)	Duplication ratio	NGA50 (bp)	Genes (%)
ALLPATHS-LG	4469	240,773	84,416,102	38,359	109	1,384,277	67.71	21.79	54.60	78.48	1.00	27,772	63
SGA	33,695	30,350	75,492,807	3317	107	249,973	87.51	12.57	0.00	69.89	1.01	1945	89
GAA	38,155	240,773	159,891,549	10,679	216	1,634,250	71.61	22.01	28.83	79.17	1.88	27,895	82
GAA	38,155	240,773	159,891,549	10,679	216	1,634,250	71.61	22.01	28.83	79.17	1.88	27,895	82
GAA	38,155	240,773	159,891,549	10,679	216	1,634,250	71.62	22.01	28.83	79.17	1.88	27,895	80
GAA	38,155	240,773	159,891,549	10,679	216	1,634,250	71.62	22.01	28.83	79.17	1.88	27,895	82
GAA	38,155	240,773	159,891,549	10,679	216	1,634,250	71.62	22.01	28.83	79.17	1.88	27,895	82
GAM_NGS	4228	240,773	84,488,919	39,589	111	1,579,215	68.19	21.80	54.50	78.55	1.00	28,518	63
GAM_NGS	4229	240,773	84,488,720	39,493	111	1,579,215	68.19	21.80	54.48	78.55	1.00	28,613	63
GAM_NGS	4258	240,773	84,476,409	39,118	109	1,545,656	68.09	21.78	54.51	78.55	1.00	28,211	63
GAM_NGS	4318	240,773	84,468,987	39,442	110	1,485,435	67.94	21.79	54.54	78.53	1.00	28,412	63
GAM_NGS	4345	240,773	84,463,742	38,828	110	1,470,117	67.97	21.79	54.56	78.53	1.00	28,129	63
GARM	4444	240,762	106,632,698	44,830	169	3,131,454	94.72	30.40	0.06	75.60	1.31	44,140	61
GARM	4445	240,762	108,298,281	45,234	232	5,969,742	95.37	30.30	0.06	75.67	1.33	45,010	61
GARM	4459	240,762	106,210,805	44,544	144	2,787,020	93.82	31.32	0.06	75.45	1.31	43,918	61
GARM	4495	240,767	106,334,207	44,486	154	2,774,708	93.24	31.74	0.06	75.48	1.31	43,839	61
GARM	4498	240,762	106,465,991	44,515	154	2,774,773	93.62	31.11	0.06	75.52	1.31	43,918	61
Metassembler	4359	240,773	84,259,313	38,473	109	1,384,277	67.54	21.81	54.50	78.36	1.00	27,772	63
Metassembler	4359	240,773	84,259,313	38,473	109	1,384,277	67.54	21.81	54.50	78.36	1.00	27,772	63
Metassembler	4359	240,773	84,259,313	38,473	109	1,384,277	67.54	21.81	54.50	78.36	1.00	27,772	63
Metassembler	4359	240,773	84,259,313	38,473	109	1,384,277	67.54	21.81	54.50	78.36	1.00	27,772	63
Metassembler	4359	240,773	84,259,313	38,473	109	1,384,277	67.54	21.81	54.50	78.36	1.00	27,772	63

Supplemental Table S4: Contiguity-correctness experimental results. Assembly reconciliation tools are given in input two assemblies to merge, in which the first has high contiguity, the second has high correctness. The table reports on quality of merged assembly compared to the two input assemblies. Notes: (c) indicates that the assembly is composed of contigs, (s) indicates that the assembly is composed of scaffolds; all reported statistics are for contigs; the number of mismatches/indels/Ns are per 100 Kbps; tools were ran using default parameters, unless otherwise noted

Reconciliation Tool or Input	Contigs (#)	Largest (bp)	Size (bp)	N50 (bp)	Misassembly (#)	Misassembly Length (bp)	Mismatches (#)	Indels (#)	N's (#)	Genome covered (%)	Duplication ratio	NGA50 (bp)	Genes (%)
S. aureus (genome size 2,903,081 bp)													
SOAPdenovo (c)	70	518,710	2,897,432	288,184	31	2,027,905	23.00	2.45	0.07	98.55	1.01	150,794	96.42
ABySS (c)	247	125,049	3,631,245	25,084	5	22,399	12.39	0.85	7.79	97.27	1.29	29,198	77.53
CISA	49	483,164	2,794,831	212,755	27	1,804,073	22.37	2.65	7.55	94.72	1.02	154,503	92.36
GAA	317	518,710	6,528,677	48,845	36	2,050,304	27.14	2.33	4.37	98.88	2.28	150,794	4.32
GAM_NGS	65	545,577	2,901,216	288,184	31	2,053,193	26.28	2.59	0.07	98.58	1.02	152,795	96.40
MIX	56	540,299	2,944,556	294,927	36	2,195,378	24.66	2.62	0.07	98.61	1.03	152,795	94.57
Metassembler	50	528,561	2,893,344	288,184	31	2,080,336	22.41	2.24	1.11	98.52	1.01	154,116	96.00
SOAPdenovo (s)	64	518,710	2,902,967	331,598	32	2,348,756	24.23	2.76	167.31	98.55	1.01	172,575	96.39
ABySS (s)	206	130,192	3,692,703	27,695	10	178,901	12.22	1.09	1520.97	97.54	1.30	31,703	77.56
CISA	51	526,000	3,030,347	331,595	37	2,502,090	26.03	2.68	489.71	97.78	1.07	172,574	90.87
GAM_NGS	56	526,202	2,904,792	335,060	32	2,358,582	25.86	2.76	174.16	98.61	1.01	181,779	96.47
GARM (ctg_scf)	41	1,055,685	7,562,761	335,611	105	7,125,530	28.46	2.34	18,510.37	72.15	3.61	217,484	5.07
GARM (scf_ctg)	5	423,440	1,485,622	410,622	14	1,478,652	27.49	2.34	12,403.76	38.32	1.33	NA	33.28
MIX	45	542,391	2,974,002	356,570	35	2,442,687	27.93	3.11	191.93	98.69	1.04	177,880	93.92
Metassembler	43	529,535	2,902,261	331,598	32	2,358,551	23.29	2.59	193.95	98.56	1.01	181,779	96.01
R. sphaeroides (genome size 4,603,060 bp)													
SOAPdenovo (c)	114	376,585	4,569,340	131,681	11	633,163	21.28	9.51	0.00	98.72	1.01	129,613	92.24
ABySS (c)	1509	54,734	4,830,769	5562	85	866,218	22.76	5.84	2.32	93.75	1.12	5303	76.12
CISA	135	157,113	2,635,836	60,566	23	429,493	45.18	11.06	0.57	55.40	1.03	19,060	49.52
GAA	1604	376,585	9,367,810	23,577	96	1,499,381	27.78	10.64	1.20	99.41	2.05	129,613	22.27
GAM_NGS	110	376,585	4,569,928	151,404	11	612,573	22.11	9.59	0.04	98.74	1.01	144,469	92.19
MIX	87	376,585	4,601,257	144,469	21	951,069	25.57	10.12	0.41	98.96	1.01	131,601	90.69
Metassembler	87	376,585	4,564,073	144,469	12	668,770	21.75	9.61	4.01	98.79	1.00	131,601	91.50
SOAPdenovo (s)	76	1,154,134	4,579,801	660,164	13	1,927,959	21.30	9.81	228.42	98.73	1.01	539,770	92.58
ABySS (s)	1352	87,855	4,968,921	8036	85	1,012,703	23.38	8.92	2302.47	94.21	1.14	7136	76.92
CISA	43	118,526	1,271,328	45,199	25	584,067	72.32	12.85	1964.87	23.67	1.17	NA	19.02
GAM_NGS	72	1,154,134	4,580,383	660,164	13	1,927,959	21.34	9.81	222.06	98.75	1.01	539,770	92.55
GARM (ctg_scf)	82	885,089	14,770,552	340,523	121	13,355,236	41.34	30.74	14,620.98	81.61	3.93	300,232	18.83
GARM (scf_ctg)	11	1,160,202	4,086,623	1,035,290	17	3,379,068	41.14	31.14	208.88	87.97	1.01	540,088	83.34
MIX	53	2,189,067	4,622,653	665,695	20	3,370,373	23.05	9.93	263.61	98.86	1.02	539,770	91.39
Metassembler	46	1,158,092	4,580,689	665,622	15	1,961,085	21.67	9.95	280.55	98.73	1.01	538,783	91.76
Homo sapiens, chromosome 14 (genome size 107,349,540 bp)													
SOAPdenovo (c)	15,028	147,494	90,398,734	16,179	6329	43,713,769	152.34	24.26	0.02	77.30	1.09	8155	64.03
ABySS (c)	32,050	30,053	67,074,140	3182	24	128,244	84.48	9.20	1.31	61.54	1.01	1319	83.85
GAA	Did not produce output files												
GAM_NGS	14,755	147,494	90,399,807	16,649	6281	44,141,293	152.57	24.43	0.09	77.36	1.09	8345	63.60
Metassembler	12,331	147,494	87,578,779	16,797	6128	43,411,953	148.18	23.98	0.02	76.38	1.07	8155	63.30
SOAPdenovo (s)	7264	1,849,511	100,880,746	381,286	8171	89,396,625	152.68	24.52	10,166.39	77.44	1.20	17,851	31.73
ABySS (s)	31,582	30,053	67,724,594	3355	37	237,738	84.67	9.37	859.44	61.61	1.02	1339	83.10
GAM_NGS	7166	1,849,511	100,347,539	368,318	8019	88,844,125	152.81	24.64	10,151.99	76.89	1.20	17,903	29.85
GARM (ctg_scf)	435	139,253	7,402,130	27,562	521	5,362,430	191.29	31.20	8979.09	5.40	1.25	NA	11.18
GARM (scf_ctg)	949	1,853,709	92,980,399	427,952	7958	89,041,639	159.13	25.85	10,863.86	72.25	1.18	17,783	22.95
Metassembler	5358	1,849,511	98,820,840	387,994	8116	89,280,078	152.75	24.55	10,227.11	76.98	1.18	17,851	28.80

Supplemental Table S5: Experimental results on merging assemblies produced by assemblers based on the de Bruijn graph compared to string graph. The table reports on quality of merged assembly compared to the two input assemblies. Notes: (c) indicates that the assembly is composed of contigs, (s) indicates that the assembly is composed of scaffolds; all reported statistics are for contigs; the number of mismatches/indels/Ns are per 100 Kbps; tools were ran using default parameters, unless otherwise noted

Reconciliation Tool or Input	Contigs (#)	Largest (bp)	Size (bp)	N50 (bp)	Misassembly (#)	Misassembly Length (bp)	Mismatches (#)	Indels (#)	N's (#)	Genome covered (%)	Duplication ratio	NGA50 (bp)	Genes (%)
S. aureus (genome size 2,903,081 bp)													
ALLPATHS-LG (c)	59	234,488	2,869,581	96,740	1	89,634	1.57	0.73	1.50	98.83	1.00	96,740	95
SGA (c)	985	16,870	2,748,664	4178	1	2431	1.02	0.11	0.00	94.44	1.00	4005	81
CISA	65	208,210	2,438,736	69,908	1	30,003	3.19	0.78	1.44	84.14	1.00	49,357	81
GAA	1037	234,488	5,599,935	16,131	2	92,065	1.63	0.73	0.77	99.11	1.95	96,740	26
GAM_NGS	59	234,488	2,869,581	96,740	1	89,634	1.57	0.73	1.50	98.83	1.00	96,740	95
MIX	59	234,488	2,869,581	96,740	1	89,634	1.57	0.73	1.50	98.83	1.00	96,740	95
Metassembler	59	234,488	2,869,780	96,740	1	89,634	1.57	0.73	1.50	98.83	1.00	96,740	95
ZORRO	69	234,548	2,873,546	96,657	0	0	1.99	0.59	0.90	98.90	1.00	96,657	96
ALLPATHS-LG (s)	11	1,435,559	2,879,481	1,091,731	0	0	3.97	2.51	345.31	98.86	1.00	1,082,616	96
SGA (s)	299	286,534	3,051,005	149,421	3	113,622	1.09	11.47	9852.72	94.87	1.11	134,849	82
CISA	4	461,617	1,286,214	286,534	0	0	0.98	8.46	7240.24	38.69	1.15	NA	35
GAM_NGS	11	1,435,559	2,879,481	1,091,731	0	0	3.97	2.51	345.31	98.86	1.00	1,082,616	96
GARM (ctg_scf)	19	657,195	4,255,326	246,153	13	2,640,794	3.58	1.52	12,687.18	97.25	1.51	231,221	64
GARM (scf_ctg)	3	1,435,569	2,707,066	1,435,569	0	0	3.60	2.85	366.04	92.92	1.00	1,082,874	91
MIX	11	1,435,559	2,879,481	1,091,731	0	0	3.97	2.51	345.31	98.86	1.00	1,082,616	96
Metassembler	11	1,435,619	2,879,843	1,092,093	0	0	3.59	1.92	503.22	98.71	1.00	1,080,404	96
ZORRO	759	48,229	3,116,392	10,351	0	0	1.06	0.70	8684.11	97.73	1.00	10,768	87
R. sphaeroides (genome size 4,603,060 bp)													
ALLPATHS-LG (c)	203	106,467	4,587,354	42,455	10	404,185	6.33	4.77	2.79	99.20	1.00	41,487	92
SGA (c)	2173	29,520	4,188,432	2530	1	4048	5.70	2.47	0.00	90.70	1.00	2280	77
CISA	233	106,467	4,239,822	31,549	10	373,275	6.61	4.79	2.81	91.67	1.00	27,262	85
GAA	2348	106,467	8,730,704	10,451	11	408,233	6.74	4.79	1.47	99.32	1.91	41,487	29
GAM_NGS	201	106,467	4,588,158	42,455	10	408,636	6.61	4.77	2.79	99.22	1.00	41,487	92
MIX	202	106,467	4,592,497	42,455	12	464,515	6.66	4.80	2.74	99.22	1.01	41,487	92
Metassembler	200	106,467	4,587,010	42,455	10	408,636	6.61	4.77	2.79	99.22	1.00	41,487	92
ZORRO	231	105,315	4,596,344	37,312	8	215,902	6.96	4.68	1.46	99.29	1.01	37,195	92
ALLPATHS-LG (s)	33	3,192,334	4,608,763	3,192,334	15	4,447,871	5.91	6.79	467.31	99.24	1.01	928,821	95
SGA (s)	1208	148,756	5,328,387	44,205	3	31,764	5.86	7.22	21,499.94	90.77	1.26	17,695	77
CISA	10	148,922	1,021,674	113,539	2	227,007	3.45	9.28	17,854.33	18.27	1.22	NA	17
GAM_NGS	31	3,192,334	4,609,567	3,192,334	15	4,452,322	6.19	6.78	467.22	99.26	1.01	928,821	95
GARM (ctg_scf)	65	238,957	6,690,621	134,426	71	5,275,835	9.44	7.40	16,050.80	86.27	1.69	73,449	51
GARM(scf_ctg)	4	3,192,749	4,371,411	3,192,749	12	4,371,411	8.48	8.87	479.98	94.29	1.01	929,045	91
MIX	32	3,192,334	4,783,485	3,192,334	16	4,496,001	6.30	6.81	1383.89	99.26	1.05	928,821	92
Metassembler	30	3,192,172	4,608,043	3,192,172	15	4,451,946	6.05	6.88	547.37	99.17	1.01	928,722	94
ZORRO	1970	105,319	5,706,683	3275	7	95,930	6.39	5.05	19,859.89	98.61	1.01	4944	82
Homo sapiens, chromosome 14 (genome size 107,349,540 bp)													
ALLPATHS-LG (c)	4469	240,773	84,416,102	38,359	109	1,384,277	67.71	21.79	54.60	78.48	1.00	27,772	62
SGA (c)	33,695	30,350	75,492,807	3317	107	249,973	87.51	12.57	0.00	69.89	1.01	1945	88
GAA	38,155	240,773	159,891,549	10,679	216	1,634,250	71.63	22.02	28.83	79.17	1.88	27,895	81
GAM_NGS	4283	240,773	84,466,433	39,449	110	1,663,046	68.05	21.79	54.51	78.53	1.00	28,458	62
Metassembler	4359	240,773	84,259,313	38,473	109	1,384,277	67.54	21.81	54.50	78.36	1.00	27,772	62
ALLPATHS-LG (s)	174	81,646,936	87,646,728	81,646,936	455	87,219,645	66.85	22.87	3734.63	78.51	1.04	397,351	14
SGA (s)	9586	551,622	88,557,645	82,616	170	8,811,457	87.77	18.34	14,499.38	70.47	1.16	35,317	34
GAM_NGS	174	81,646,936	87,646,728	81,646,936	455	87,219,645	66.85	22.87	3734.63	78.51	1.04	397,351	14
GARM (ctg_scf)	1186	671,738	123,685,540	148,610	1160	86,820,549	92.01	31.12	13,901.57	75.55	1.52	84,526	37
GARM (scf_ctg)	30	81,832,553	87,591,308	81,832,553	496	87,461,370	91.83	31.93	3459.35	78.17	1.04	359,840	NA
Metassembler	137	81,646,936	87,584,806	81,646,936	455	87,219,645	66.85	22.87	3737.20	78.50	1.04	397,351	14

Supplemental Table S6: Contiguity-correctness experimental results. Assembly reconciliation tools are given in input the same two assemblies in Supplemental Table S4, but the order is swapped. The table reports on quality of merged assembly compared to the two input assemblies. Notes: (c) indicates that the assembly is composed of contigs, (s) indicates that the assembly is composed of scaffolds; all reported statistics are for contigs; the number of mismatches/indels/Ns are per 100 Kbps; tools were ran using default parameters, unless otherwise noted

Reconciliation Tool or Input	Contigs (#)	Largest (bp)	Size (bp)	N50 (bp)	Misassembly (#)	Misassembly Length (bp)	Mismatches (#)	Indels (#)	N's (#)	Genome covered (%)	Duplication ratio	NGA50 (bp)	Genes (%)
S. aureus (genome size 2,903,081 bp)													
ABySS (c)	247	125,049	3,631,245	25,084	5	22,399	12.39	0.85	7.79	97.27	1.29	29,198	77
SOAPdenovo (c)	70	518,710	2,897,432	288,184	31	2,027,905	23.00	2.45	0.07	98.55	1.01	150,794	96
CISA	49	483,164	2,794,831	212,755	27	1,804,073	22.37	2.65	7.55	94.72	1.02	154,503	92
GAA	316	518,710	6,524,766	48,845	36	2,050,304	27.21	2.33	4.37	98.88	2.27	150,794	4
GAM_NGS	171	280,341	3,696,361	39,384	20	739,372	15.26	0.99	6.41	97.73	1.30	69,239	78
MIX	57	522,777	2,893,314	294,927	33	2,086,001	23.65	2.59	0.10	98.59	1.01	152,795	96
Metassembler	51	524,335	2,945,348	179,402	28	1,642,287	22.90	2.35	25.09	98.23	1.03	154,503	94
ABySS (s)	206	130,192	3,692,703	27,695	10	178,901	12.22	1.09	1520.97	97.54	1.30	31,703	77
SOAPdenovo (s)	64	518,710	2,902,967	331,598	32	2,348,756	24.23	2.76	167.31	98.55	1.01	172,575	96
CISA	51	526,000	3,030,347	331,595	37	2,502,090	26.03	2.68	489.71	97.78	1.07	172,574	90
GAM_NGS	90	474,493	3,626,574	130,192	28	1,841,289	21.65	2.14	1416.24	98.18	1.27	123,783	78
GARM (ctg_scf)	41	1,055,685	7,562,761	335,611	105	7,125,530	28.46	2.34	18,510.37	72.15	3.61	217,484	5
GARM (scf_ctg)	5	410,638	1,445,727	383,625	14	1,438,757	23.97	2.43	9991.24	38.32	1.30	NA	33
MIX	44	524,869	2,923,103	356,570	37	2,405,808	26.96	2.93	209.50	98.66	1.02	177,880	95
Metassembler	63	410,149	3,395,040	172,462	28	1,443,578	24.03	2.52	951.56	98.34	1.19	155,925	84
R. sphaeroides (genome size 4,603,060 bp)													
ABySS (c)	1509	54,734	4,830,769	5562	85	866,218	22.76	5.84	2.32	93.75	1.12	5303	76
SOAPdenovo (c)	114	376,585	4,569,340	131,681	11	633,163	21.28	9.51	0.00	98.72	1.01	129,613	92
CISA	135	157,113	2,635,836	60,566	23	429,493	45.18	11.06	0.57	55.40	1.03	19,060	49
GAA	1622	376,585	9,398,024	23,115	96	1,499,381	27.78	10.64	1.19	99.41	2.05	129,613	22
GAM_NGS	704	158,412	4,953,882	22,244	86	991,638	28.82	10.10	2.28	96.57	1.11	22,244	80
MIX	87	382,294	4,606,966	144,469	26	1,500,909	26.77	10.04	0.41	98.86	1.01	129,613	90
Metassembler	196	190,287	4,841,862	65,553	77	2,242,683	28.67	12.22	42.63	98.27	1.07	58,769	88
ABySS (s)	1352	87,855	4,968,921	8036	85	1,012,703	23.38	8.92	2302.47	94.21	1.14	7136	76
SOAPdenovo (s)	76	1,154,134	4,579,801	660,164	13	1,927,959	21.30	9.81	228.42	98.73	1.01	539,770	92
CISA	43	118,526	1,271,328	45,199	25	584,067	72.32	12.85	1964.87	23.67	1.17	NA	19
GAM_NGS	1328	87,855	4,982,719	8240	85	1,022,301	22.80	9.49	2326.56	94.48	1.14	7266	76
GARM (scf_ctg)	82	885,089	14,770,552	340,523	121	13,355,236	41.34	30.74	14,620.98	81.61	3.93	300,232	18
GARM (ctg_scf)	11	1,160,145	4,086,020	1,035,309	17	3,378,435	38.95	31.10	207.46	87.95	1.01	540,075	83
MIX	53	2,194,776	4,628,362	665,695	22	4,041,777	22.83	10.06	263.29	98.86	1.02	539,765	91
Metassembler	183	206,656	5,025,412	84,139	86	2,832,228	25.66	15.28	1664.02	98.22	1.11	82,101	86
Homo sapiens, chromosome 14 (genome size 107,349,540 bp)													
ABySS (c)	32,050	30,053	67,074,140	3182	24	128,244	84.48	9.20	1.31	61.54	1.01	1319	83
SOAPdenovo (c)	15,028	147,494	90,398,734	16,179	6329	43,713,769	152.34	24.26	0.02	77.30	1.09	8155	64
GAA	41,007	147,494	150,670,123	7865	6329	43,803,415	155.06	24.61	0.60	76.10	1.84	9733	79
GAM_NGS	17,579	146,388	76,617,304	11,218	2853	24,315,403	110.27	16.87	1.01	68.83	1.04	4216	67
Metassembler	31,909	30,053	66,662,156	3168	22	124,338	84.37	9.20	1.28	61.52	1.01	1291	77
ABySS (s)	31,582	30,053	67,724,594	3355	37	237,738	84.67	9.37	859.44	61.61	1.02	1339	83
SOAPdenovo (s)	7264	1,849,511	100,880,746	381,286	8171	89,396,625	152.68	24.52	10,166.39	77.44	1.20	17,851	31
GAM_NGS	24,913	183,079	76,225,840	5205	1425	11,646,550	102.35	13.54	4323.03	65.94	1.06	2278	76
GARM (ctg_scf)	435	139,253	7,402,130	27,562	521	5,362,430	191.29	31.20	8979.09	5.40	1.25	NA	11
Metassembler	31,467	30,053	67,494,412	3359	36	232,366	84.49	9.36	659.65	61.59	1.02	1334	76

Supplemental Table S7: Experimental results on merging high-quality assemblies. The table reports on quality of merged assembly compared to the two input assemblies. Notes: (c) indicates that the assembly is composed of contigs, (s) indicates that the assembly is composed of scaffolds; all reported statistics are for contigs; the number of mismatches/indels/Ns are per 100 Kbps; tools were ran using default parameters, unless otherwise noted

Reconciliation Tool or Input	Contigs (#)	Largest (bp)	Size (bp)	N50 (bp)	Misassembly (#)	Misassembly Length (bp)	Mismatches (#)	Indels (#)	N's (#)	Genome covered (%)	Duplication ratio	NGA50 (bp)	Genes (%)
S. aureus (genome size 2,903,081 bp)													
ALLPATHS-LG (c)	59	234,488	2,869,581	96,740	1	89,634	1.57	0.73	1.50	98.83	1.00	96,740	95.72
MSR-CA (c)	89	139,438	2,860,132	59,152	20	641,173	20.67	1.82	0.00	98.17	1.00	55,068	95.16
CISA	31	480,850	2,901,690	107,125	7	887,865	9.45	1.09	1.17	97.71	1.02	107,125	93.10
GAA	147	234,488	5,728,434	69,048	20	729,528	11.26	1.66	0.75	99.41	1.99	109,325	3.62
GAM_NGS	45	474,996	2,871,133	161,072	0	0	2.65	0.94	1.43	98.91	1.00	161,072	95.91
MIX	37	593,709	3,110,176	200,247	22	1,910,115	8.27	1.67	1.22	99.08	1.08	101,091	59.86
Metassembler	29	481,089	2,853,430	200,247	2	34,427	3.05	0.91	4.66	98.32	1.00	200,247	95.38
ZORRO	72	201,529	2,885,456	70,361	4	121,296	5.63	1.39	0.42	99.13	1.00	68,006	96.19
ALLPATHS-LG (s)	11	1,435,559	2,879,481	1,091,731	0	0	3.97	2.51	345.31	98.86	1.00	1,082,616	96.59
MSR-CA (s)	13	2,411,914	2,871,405	2,411,914	49	2,806,230	20.90	3.75	360.56	98.23	1.01	220,020	96.65
CISA	10	2,411,914	2,865,036	2,411,914	47	2,857,360	20.87	3.71	308.93	98.39	1.00	220,020	96.82
GAM_NGS	11	1,435,559	2,879,481	1,091,731	0	0	3.97	2.51	345.31	98.86	1.00	1,082,616	96.59
GARM (ctg_scf)	4	2,612,640	3,154,342	2,612,640	44	3,154,342	13.57	2.78	168.66	96.49	1.13	255,111	91.99
GARM (scf_ctg)	3	1,531,987	2,931,536	1,531,987	12	2,931,536	14.95	4.15	118.47	90.58	1.12	356,937	89.19
MIX	10	3,844,359	5,680,169	3,844,359	54	5,596,862	15.08	4.57	336.91	99.39	1.97	1,077,568	88.08
Metassembler	7	1,435,836	2,860,386	1,435,836	2	1,435,836	3.40	1.61	395.02	98.15	1.00	1,082,089	96.02
ZORRO	124	178,381	2,943,696	57,402	6	48,675	3.83	1.78	478.82	98.95	1.02	60,015	94.26
R. sphaeroides (genome size 4,603,060 bp)													
ALLPATHS-LG (c)	203	106,467	4,587,354	42,455	10	404,185	6.33	4.77	2.79	99.20	1.00	41,487	92.55
SOAPdenovo (c)	114	376,585	4,569,340	131,681	11	633,163	21.28	9.51	0.00	98.72	1.01	129,613	92.24
CISA	56	423,736	4,826,390	131,681	13	824,841	15.78	8.86	0.79	97.86	1.07	131,601	84.89
GAA	316	376,585	9,152,896	67,208	21	1,037,348	14.43	7.03	1.40	99.53	2.00	129,613	6.01
GAM_NGS	147	259,855	4,589,116	65,618	14	515,689	12.13	5.73	2.68	99.26	1.00	60,502	92.61
MIX	79	400,670	4,962,938	180,222	29	2,386,110	24.31	9.21	0.60	98.86	1.09	131,601	85.54
Metassembler	65	446,066	4,588,876	187,667	17	1,358,099	11.75	6.78	47.24	99.32	1.00	187,667	93.03
ZORRO	291	93,996	4,603,056	27,399	12	183,417	11.31	4.63	1.02	99.09	1.01	27,233	91.83
ALLPATHS-LG (s)	33	3,192,334	4,608,763	3,192,334	15	4,447,871	5.91	6.79	467.31	99.24	1.01	928,821	95.02
SOAPdenovo (s)	76	1,154,134	4,579,801	660,164	13	1,927,959	21.30	9.81	228.42	98.73	1.01	539,770	92.58
CISA	32	1,331,947	1,824,146	1,331,947	12	1,660,662	23.74	6.44	238.36	39.44	1.01	NA	37.97
GAM_NGS	29	3,192,334	4,609,305	3,192,334	15	4,449,040	5.91	6.83	467.14	99.26	1.01	928,821	95.13
GARM (ctg_scf)	11	1,164,408	4,508,978	1,115,528	23	4,344,826	21.86	11.59	196.65	89.25	1.10	248,291	82.83
GARM (scf_ctg)	4	4,425,980	6,151,505	4,425,980	34	6,151,505	12.66	8.34	2128.16	94.53	1.41	358,682	91.36
MIX	28	3,192,334	4,720,129	3,192,334	14	4,550,518	6.08	6.87	456.15	99.27	1.03	928,821	95.01
Metassembler	30	3,191,637	4,606,469	3,191,637	16	4,446,954	6.48	6.83	421.10	99.29	1.01	929,418	95.04
ZORRO	272	117,853	4,626,121	32,447	10	134,705	9.08	5.86	434.79	99.05	1.01	32,447	92.30
Homo sapiens, chromosome 14 (genome size 107,349,540 bp)													
ALLPATHS-LG (c)	4469	240,773	84,416,102	38,359	109	1,384,277	67.71	21.79	54.60	78.48	1.00	27,772	62.80
CABOG (c)	3233	296,904	86,189,919	46,699	108	3,694,326	101.52	23.29	0.00	79.94	1.00	35,539	59.37
GAA	7687	296,904	170,593,729	42,872	217	5,078,603	94.38	23.82	27.02	80.36	1.98	62,647	70.82
GAM_NGS	2916	483,603	84,926,958	65,003	107	2,715,562	70.71	22.69	52.70	78.99	1.00	45,596	55.98
Metassembler	4362	240,773	84,241,180	38,473	109	1,432,983	67.61	21.80	54.53	78.37	1.00	27,830	62.73
ALLPATHS-LG (s)	174	81,646,936	87,646,728	81,646,936	455	87,219,645	66.85	22.87	3734.63	78.51	1.04	397,351	14.00
CABOG (s)	474	2,260,562	86,481,568	401,279	269	43,205,905	101.10	24.68	267.20	80.01	1.01	215,699	29.56
GAM_NGS	171	81,646,936	87,651,696	81,646,936	455	87,219,645	66.89	22.88	3734.42	78.51	1.04	397,351	21.43
GARM (ctg_scf)	297	2,558,470	96,444,763	501,662	544	77,192,101	91.38	24.79	3207.84	76.15	1.18	195,601	27.05
GARM (scf_ctg)	23	200,905,823	207,730,622	200,905,823	106	6,711,411	164.08	37.12	55,599.20	78.67	1.09	74,709	18.48
Metassembler	117	81,646,936	87,530,459	81,646,936	454	87,213,926	66.80	22.86	3731.03	78.46	1.04	397,351	14.37

Supplemental Table S8: Experimental results on merging highly fragmented assemblies. The table reports on quality of merged assembly compared to the two input assemblies. Notes: (c) indicates that the assembly is composed of contigs, (s) indicates that the assembly is composed of scaffolds; all reported statistics are for contigs; the number of mismatches/indels/Ns are per 100 Kbps; tools were ran using default parameters, unless otherwise noted

Reconciliation Tool or Input	Contigs (#)	Largest (bp)	Size (bp)	N50 (bp)	Misassembly (#)	Misassembly Length (bp)	Mismatches (#)	Indels (#)	N's (#)	Genome covered (%)	Duplication ratio	NGA50 (bp)	Genes (%)
S. aureus (genome size 2,903,081 bp)													
ABySS (c)	247	125,049	3,631,245	25,084	5	22,399	12.39	0.85	7.79	97.27	1.29	29,198	77.53
SGA (c)	985	16,870	2,748,664	4178	1	2431	1.02	0.11	0.00	94.44	1.00	4005	81.58
CISA	183	125,049	2,775,035	25,585	6	44,257	14.86	1.23	4.58	95.07	1.00	23,610	92.52
GAA	1225	125,049	6,361,589	10,544	6	24,830	11.82	0.77	4.45	97.93	2.24	29,198	27.27
GAM_NGS	223	125,383	3,634,133	27,931	5	22,399	13.19	0.92	7.79	97.38	1.28	31,393	77.45
MIX	176	125,049	2,922,239	27,931	14	217,753	12.57	1.60	9.68	97.03	1.04	27,666	92.69
Metassembler	203	125,049	3,480,529	28,148	4	19,126	11.55	0.82	11.58	97.19	1.23	31,180	77.72
ABySS (s)	206	130,192	3,692,703	27,695	10	178,901	12.22	1.09	1520.97	97.54	1.30	31,703	77.56
SGA (s)	299	286,534	3,051,005	149,421	3	113,622	1.09	11.47	9852.72	94.87	1.11	134,849	82.67
CISA	31	130,192	1,937,031	81,084	4	192,275	6.30	7.68	6307.75	54.72	1.22	42,752	45.91
GAM_NGS	203	130,192	3,667,521	27,917	10	178,901	12.22	1.09	1518.22	97.56	1.29	31,703	77.53
GARM (ctg_scf)	24	348,780	3,286,103	230,715	26	2,287,325	10.48	2.52	5161.68	93.00	1.15	124,186	86.50
GARM (scf_ctg)	53	248,986	1,603,258	46,033	25	736,257	32.17	6.48	4270.49	30.86	1.78	7132	19.50
MIX	278	316,106	3,167,023	149,421	10	801,229	3.55	11.31	9401.92	95.06	1.15	134,849	80.70
Metassembler	116	233,465	3,613,534	63,433	10	195,410	11.69	1.91	1587.67	97.28	1.28	87,846	76.71
R. sphaeroides (genome size 4,603,060 bp)													
ABySS (c)	1509	54,734	4,830,769	5562	85	866,218	22.76	5.84	2.32	93.75	1.12	5303	76.12
SGA (c)	2173	29,520	4,188,432	2530	1	4048	5.70	2.47	0.00	90.70	1.00	2280	77.99
CISA	1343	54,734	4,586,141	5631	62	655,971	23.40	5.79	1.98	94.51	1.05	5082	80.07
GAA	3652	54,734	8,975,227	3515	86	870,266	22.41	5.41	1.25	95.96	2.03	6712	33.05
GAM_NGS	1394	54,734	4,843,138	5823	82	870,277	22.86	5.98	2.31	94.08	1.12	5626	77.03
MIX	1442	54,734	4,670,901	5638	111	964,749	19.87	7.28	2.21	93.72	1.08	4845	77.37
Metassembler	1466	54,734	4,792,900	5705	81	876,669	22.88	5.84	2.34	93.70	1.11	5435	75.43
ABySS (s)	1352	87,855	4,968,921	8036	85	1,012,703	23.38	8.92	2302.47	94.21	1.14	7136	76.92
SGA (s)	1208	148,756	5,328,387	44,205	3	31,764	5.86	7.22	21,499.94	90.77	1.26	17,695	77.90
CISA	154	96,941	3,878,079	28,615	28	535,930	17.65	11.64	19,413.30	59.62	1.41	15,332	49.80
GAM_NGS	1227	87,855	5,088,762	9076	85	1,029,168	22.28	9.29	4248.87	94.46	1.16	8029	77.09
GARM (ctg_scf)	106	166,599	4,632,346	68,824	186	4,110,632	20.05	12.48	18,414.95	73.91	1.36	17,133	65.89
GARM (scf_ctg)	178	87,870	1,912,210	15,350	52	634,591	30.78	17.47	5630.66	34.12	1.21	NA	29.65
MIX	1136	148,756	5,568,950	50,549	29	801,324	6.98	8.57	20,654.38	91.09	1.31	21,096	74.61
Metassembler	945	87,855	5,247,377	13,289	80	1,160,977	22.92	10.08	8078.86	94.37	1.20	11,657	77.55
Homo sapiens, chromosome 14 (genome size 107,349,540 bp)													
ABySS (c)	32,050	30,053	67,074,140	3182	24	128,244	84.48	9.20	1.31	61.54	1.01	1319	83.85
SGA (c)	33,695	30,350	75,492,807	3317	107	249,973	87.51	12.57	0.00	69.89	1.01	1945	88.96
GAA	65,737	30,350	142,552,348	3255	131	378,217	90.36	13.71	0.62	70.52	1.88	4336	83.60
GAM_NGS	28,291	52,608	69,320,650	3757	29	161,851	85.25	9.96	1.18	63.74	1.01	1753	82.70
Metassembler	31,853	30,053	66,646,160	3174	22	124,338	84.13	9.18	1.28	61.48	1.01	1293	76.87
ABySS (s)	31,582	30,053	67,724,594	3355	37	237,738	84.67	9.37	859.44	61.61	1.02	1339	83.10
SGA (s)	9586	551,622	88,557,645	82,616	170	8,811,457	87.77	18.34	14,499.38	70.47	1.16	35,317	34.97
GAM_NGS	16,084	168,748	80,212,474	15,869	115	1,945,058	86.43	14.31	10,009.71	66.58	1.11	4213	57.79
GARM (ctg_scf)	1751	555,426	82,819,623	91,759	277	18,242,778	88.50	17.64	15,041.66	64.98	1.17	34,725	26.63
GARM (scf_ctg)	627	30,127	4,114,216	8195	53	471,071	126.00	21.41	12,030.12	3.09	1.18	NA	NA
Metassembler	31,401	30,053	67,410,722	3359	34	209,172	84.30	9.34	649.81	61.55	1.02	1331	76.12

Supplemental Table S9: Experimental results on merging more than two assemblies ordered by the FRCurve score (*S. aureus*, genome size: 2,903,081 bp). The table reports on quality of merged assembly compared to the two input assemblies. Notes: all reported statistics are for contigs; the number of mismatches/indels/Ns are per 100 Kbps; tools were ran using default parameters, unless otherwise noted; (1+2)+3 means that assembly 1 and 2 were merged first, the result of which was then merged to assembly 3

Reconciliation Tool or Input	Contigs (#)	Largest (bp)	Size (bp)	N50 (bp)	Misassembly (#)	Misassembly Length (bp)	Mismatches (#)	Indels (#)	N's (#)	Genome covered (%)	Duplication ratio	NGA50 (bp)	Genes (%)
Input 1 (MSR-CA)	89	139,438	2,860,132	59,152	20	641,173	20.67	1.82	0.00	98.17	1.00	55,068	95
Input 2 (ALLPATHS-LG)	59	234,488	2,869,581	96,740	1	89,634	1.57	0.73	1.50	98.83	1.00	96,740	96
Input 3 (BAMBUS2)	106	158,330	2,832,623	50,192	1	9411	1.41	6.95	0.35	97.66	1.00	50,192	95
Input 4 (SGA)	985	16,870	2,748,664	4178	1	2431	1.02	0.11	0.00	94.44	1.00	4005	82
Input 5 (Velvet)	128	169,214	2,837,036	52,792	9	284,379	13.51	1.69	0.00	97.73	1.00	48,149	97
Input 6 (SOAPdenovo)	70	518,710	2,897,432	288,184	31	2,027,905	23.00	2.45	0.07	98.55	1.01	150,794	96
Input 7 (ABySS)	247	125,049	3,631,245	25,084	5	22,399	12.39	0.85	7.79	97.27	1.29	29,198	78
CISA (1+2)	31	480,850	2,901,690	107,125	7	887,865	9.45	1.09	1.17	97.71	1.02	107,125	93
CISA (1+2+3)	26	384,401	2,888,221	139,438	9	1,106,426	6.22	2.12	1.45	92.43	1.08	139,438	84
CISA (1+2+3+4)	25	384,489	2,898,535	139,438	10	1,200,735	6.30	2.16	1.45	92.43	1.08	139,438	83
CISA (1+2+...+5)	24	390,086	2,869,762	171,871	8	976,622	5.26	1.87	1.43	90.39	1.09	149,829	81
CISA (1+2+...+6)	17	521,399	3,094,516	288,204	25	2,175,309	17.21	2.68	0.65	92.46	1.15	179,344	77
CISA (1+2+...+7)	17	521,399	3,094,993	288,204	27	2,286,203	17.25	2.72	0.87	92.48	1.15	179,344	77
GAA (1+2)						Did not produce an assembly file							
GAM_NGS (1+2)	57	254,779	2,862,666	83,138	19	816,715	20.33	1.82	0.21	98.27	1.00	77,039	95
GAM_NGS ((1+2)+3)	52	360,903	2,861,820	90,479	19	1,177,440	20.20	1.93	0.21	98.24	1.00	82,101	96
GAM_NGS (((1+2)+3)+4)	52	360,903	2,861,820	90,479	19	1,177,440	20.20	1.93	0.21	98.24	1.00	82,101	96
GAM_NGS (((1+2)+...)+5)	49	360,903	2,861,801	139,061	17	1,163,920	20.20	1.93	0.21	98.24	1.00	100,381	96
GAM_NGS (((1+2)+...)+6)	45	360,903	2,861,888	139,061	18	1,284,451	20.55	1.93	0.21	98.25	1.00	110,995	96
GAM_NGS (((1+2)+...)+7)	44	360,903	2,861,951	168,739	19	1,538,807	20.41	1.93	0.21	98.25	1.00	110,995	96
GARM (1+2)	16	833,732	3,469,519	625,562	7	2,559,729	14.53	3.69	0.09	93.38	1.28	325,955	65
GARM ((1+2)+3)	16	833,738	3,554,698	625,563	8	2,524,523	11.80	3.92	0.23	93.11	1.30	330,720	64
GARM (((1+2)+3)+4)	1	634,995	634,995	634,995	4	634,995	14.10	4.59	1.42	21.74	1.01	NA	23
GARM (((1+2)+...)+5)	2	625,576	634,987	625,576	3	634,987	17.90	5.07	0.00	21.74	1.01	NA	22
GARM (((1+2)+...)+6)	11	316,522	1,117,006	152,795	10	794,721	29.53	2.44	0.00	38.15	1.01	NA	39
GARM (((1+2)+...)+7)	79	316,549	2,768,200	239,944	24	1,756,442	31.13	1.72	4.84	43.93	2.17	152,817	16
Metassembler (1+2)	33	315,232	2,855,905	172,800	12	1,096,894	20.30	1.47	0.28	98.25	1.00	139,438	95
Metassembler ((1+2)+3)	29	356,601	2,856,200	186,644	13	1,207,911	19.99	1.54	0.28	98.24	1.00	149,559	95
Metassembler (((1+2)+3)+4)	29	356,601	2,856,200	186,644	13	1,207,911	19.99	1.54	0.28	98.24	1.00	149,559	95
Metassembler (((1+2)+...)+5)	29	356,601	2,856,454	186,644	13	1,208,165	19.99	1.54	0.28	98.24	1.00	149,559	95
Metassembler (((1+2)+...)+6)	19	356,601	2,862,874	263,604	17	1,846,745	22.70	1.86	0.35	98.33	1.00	203,830	95
Metassembler (((1+2)+...)+7)	19	356,601	2,868,370	263,604	17	1,846,745	22.70	1.86	0.35	98.33	1.01	203,830	95
MIX (1+2)	23	200,247	766,204	66,230	1	89,634	3.52	1.17	1.70	26.39	1.00	NA	26
MIX (1+2+3)	16	200,247	545,364	80,813	0	0	3.85	0.37	2.02	18.78	1.00	NA	20
MIX (1+2+3+4)	18	200,247	560,310	80,813	0	0	3.75	0.89	1.96	19.29	1.00	NA	20
MIX (1+2+...+5)	12	200,247	412,282	69,391	0	0	1.94	1.21	2.67	14.20	1.00	NA	15
MIX (1+2+...+6)	25	518,710	1,292,513	316,522	17	1,019,107	26.74	3.11	0.00	44.31	1.01	NA	44
MIX (1+2)+...+7)	22	518,710	1,280,637	316,522	16	1,014,066	21.77	2.66	0.00	43.98	1.00	NA	44
ZORRO (1+2)	71	201,636	2,885,411	70,361	4	121,394	11.92	2.12	0.49	99.14	1.00	68,006	96
ZORRO ((1+2)+3)	114	96,442	2,881,965	43,462	4	37,282	10.47	2.19	0.35	99.01	1.00	43,461	96
ZORRO (((1+2)+3)+4)	109	136,629	2,887,028	46,362	4	37,271	10.85	1.81	0.59	99.03	1.00	46,362	96
ZORRO (((1+2)+...)+5)						Produced an empty assembly file							

Supplemental Table S10: Experimental results on merging more than two assemblies (contigs) ordered by the FRCurve score (*R. sphaeroides*, genome size 4,603,060 bp). The table reports on quality of merged assembly compared to the two input assemblies. Notes: all reported statistics are for contigs; the number of mismatches/indels/Ns are per 100 Kbps; tools were ran using default parameters, unless otherwise noted; (1+2)+3 means that assembly 1 and 2 were merged first, the result of which was then merged to assembly 3

Reconciliation Tool or Input	Contigs (#)	Largest (bp)	Size (bp)	N50 (bp)	Misassembly (#)	Misassembly Length (bp)	Mismatches (#)	Indels (#)	N's (#)	Genome covered (%)	Duplication ratio	NGA50 (bp)	Genes (%)
Input 1 (MSR-CA)	377	83,726	4,458,952	23,575	18	151,790	23.43	4.90	0.00	96.14	1.01	21,236	89
Input 2 (ALLPATHS-LG)	203	106,467	4,587,354	42,455	10	404,185	6.33	4.77	2.79	99.20	1.00	41,487	93
Input 3 (CABOG)	318	88,519	4,236,663	22,044	11	276,929	29.07	5.48	0.00	91.91	1.00	19,076	87
Input 4 (BAMBUS2)	170	279,125	4,369,357	97,331	4	123,417	5.82	5.84	0.00	94.89	1.00	93,198	90
Input 5 (SOAPdenovo)	114	376,585	4,569,340	131,681	11	633,163	21.28	9.51	0.00	98.72	1.01	129,613	92
Input 6 (Velvet)	482	60,714	4,470,215	16,033	6	127,247	8.58	4.06	0.00	96.94	1.00	15,439	92
Input 7 (SGA)	2173	29,520	4,188,432	2530	1	4048	5.70	2.47	0.00	90.70	1.00	2280	78
Input 8 (ABySS)	1509	54,734	4,830,769	5562	85	866,218	22.76	5.84	2.32	93.75	1.12	5303	76
CISA (1+2)	178	119,461	4,613,681	48,392	10	299,921	12.50	5.52	2.08	97.69	1.03	47,689	90
CISA ((1+2)+3)	147	119,461	4,677,881	50,889	16	408,918	22.74	5.45	1.73	97.33	1.05	48,389	89
CISA (((1+2)+3)+4)	68	279,125	4,997,864	97,454	16	588,100	29.68	7.58	0.56	92.31	1.18	104,509	73
CISA (((1+2)+...)+5)	39	377,514	5,072,379	154,477	18	1,224,227	32.58	10.63	0.30	89.95	1.23	154,477	67
CISA (((1+2)+...)+6)	38	377,514	5,008,389	154,477	18	1,224,227	32.48	10.72	0.30	89.95	1.21	154,477	68
CISA (((1+2)+...)+7)	38	377,567	5,008,474	154,509	18	1,224,227	32.48	10.72	0.30	89.95	1.21	154,509	68
CISA (((1+2)+...)+8)	38	377,567	5,009,192	154,509	18	1,224,227	32.48	10.72	0.30	89.96	1.21	154,509	68
GAA (1+2)						Did not produce an assembly file							
GAM_NGS (1+2)	187	201,120	4,466,857	49,612	20	266,620	23.33	6.54	0.18	96.27	1.01	44,822	91
GAM_NGS ((1+2)+3)	182	201,120	4,466,712	49,612	20	266,620	23.27	6.54	0.18	96.27	1.01	44,822	90
GAM_NGS (((1+2)+3)+4)	177	201,120	4,470,093	49,612	21	315,092	23.31	6.52	0.18	96.36	1.01	47,869	91
GAM_NGS (((1+2)+...)+5)	135	201,120	4,470,366	74,545	21	318,137	23.47	7.06	0.18	96.36	1.01	73,441	91
GAM_NGS (((1+2)+...)+6)	132	201,120	4,471,653	81,570	21	362,068	24.07	7.14	0.18	96.39	1.01	77,229	90
GAM_NGS (((1+2)+...)+7)	132	201,120	4,471,957	81,570	21	362,068	24.07	7.12	0.18	96.40	1.01	77,229	90
GAM_NGS (((1+2)+...)+8)	131	201,120	4,471,960	81,570	21	390,907	24.16	7.12	0.18	96.40	1.01	75,898	90
GARM (1+2)	63	287,164	4,330,697	110,067	12	754,372	10.33	7.41	1.18	90.85	1.02	104,539	86
GARM ((1+2)+3)	6	222,843	682,218	191,001	2	191,001	9.26	6.83	3.52	14.31	1.04	NA	14
Metassembler (1+2)	158	201,129	4,456,882	50,989	14	251,647	24.31	5.60	0.56	96.59	1.00	48,911	91
Metassembler ((1+2)+3)	148	201,129	4,296,041	50,989	10	196,260	23.00	5.65	0.33	93.12	1.00	45,415	88
Metassembler (((1+2)+3)+4)	139	201,129	4,292,624	53,825	10	196,260	22.69	5.70	0.33	93.05	1.00	47,869	88
Metassembler (((1+2)+...)+5)	94	213,406	4,318,507	74,545	11	265,268	22.26	6.29	0.32	93.58	1.00	67,149	89
Metassembler (((1+2)+...)+6)	94	213,406	4,318,507	74,545	11	265,268	22.26	6.29	0.32	93.58	1.00	67,149	89
Metassembler (((1+2)+...)+7)	94	213,406	4,318,507	74,545	11	265,268	22.26	6.29	0.32	93.58	1.00	67,149	89
Metassembler (((1+2)+...)+8)	94	213,307	4,317,739	74,545	11	265,268	22.27	6.29	0.32	93.56	1.00	67,149	89
MIX (1+2)	150	106,467	3,059,487	41,925	7	286,990	7.10	5.04	2.55	66.42	1.00	20,550	64
MIX ((1+2)+3)	133	106,467	2,823,064	41,925	7	286,990	7.37	5.25	2.27	61.29	1.00	18,408	59
MIX (((1+2)+3)+4)	92	167,490	2,083,309	93,198	2	31,469	5.14	5.19	0.00	45.24	1.00	NA	43
MIX (((1+2)+...)+5)	38	376,585	1,727,015	162,015	4	176,145	18.10	7.98	0.00	37.57	1.00	NA	36
MIX (((1+2)+...)+6)	43	376,585	1,612,424	131,681	4	176,145	24.32	8.19	0.00	35.01	1.00	NA	34
MIX (((1+2)+...)+7)	44	376,585	1,813,898	162,015	4	176,145	21.89	7.94	0.00	39.40	1.00	NA	38
MIX (((1+2)+...)+8)	40	376,585	1,870,882	162,015	3	154,488	21.65	8.18	0.00	40.64	1.00	NA	39
ZORRO (1+2)	202	201,129	4,647,602	47,869	15	277,329	21.61	5.81	1.12	99.52	1.01	43,630	93
ZORRO ((1+2)+3)	197	164,959	4,647,457	44,633	15	277,032	22.25	5.87	1.20	99.50	1.01	42,476	93
ZORRO (((1+2)+3)+4)	401	105,289	4,575,721	18,953	13	221,587	20.84	5.38	0.46	97.77	1.02	18,719	90
ZORRO (((1+2)+...)+5)	390	88,474	4,716,386	21,369	15	258,783	22.95	5.77	0.81	99.11	1.03	21,369	89
ZORRO (((1+2)+...)+6)						Produced an empty assembly file							

Supplemental Table S11: Experimental results on merging more than two assemblies (contigs) ordered by the FRCurve score (*Hg_chr14*, genome size 107,349,540 bp). The table reports on quality of merged assembly compared to the two input assemblies. Notes: all reported statistics are for contigs; the number of mismatches/indels/Ns are per 100 Kbps; tools were ran using default parameters, unless otherwise noted; (1+2)+3 means that assembly 1 and 2 were merged first, the result of which was then merged to assembly 3

Reconciliation Tool or Input	Contigs (#)	Largest (bp)	Size (bp)	N50 (bp)	Misassembly (#)	Misassembly Length (bp)	Mismatches (#)	Indels (#)	N's (#)	Genome covered (%)	Duplication ratio	NGA50 (bp)	Genes (%)
Input 1 (CABOG)	3233	296,904	86,189,919	46,699	108	3,694,326	101.52	23.29	0.00	79.94	1.00	35,539	59
Input 2 (ALLPATHS-LG)	4469	240,773	84,416,102	38,359	109	1,384,277	67.71	21.79	54.60	78.48	1.00	27,772	63
Input 3 (ABySS)	32,050	30,053	67,074,140	3182	24	128,244	84.48	9.20	1.31	61.54	1.01	1319	84
Input 4 (SOAPdenovo)	15,028	147,494	90,398,734	16,179	6329	43,713,769	152.34	24.26	0.02	77.30	1.09	8155	64
Input 5 (MSR-CA)	25,022	53,925	81,485,144	5470	2038	6,589,712	220.87	24.66	0.00	74.21	1.02	3427	83
Input 6 (BAMBUS)	12,396	736,657	67,814,016	8500	2973	12,211,265	104.23	22.18	0.01	62.52	1.01	3218	62
Input 7 (SGA)	33,695	30,350	75,492,807	3317	107	249,973	87.51	12.57	0.00	69.89	1.01	1945	89
Input 8 (Velvet)	32,842	27,872	70,575,215	3087	232	602,910	104.51	21.41	0.00	65.33	1.00	1580	82
GAA (1+2)	Did not produce an assembly file												
GAM_NGS (1+2)	1828	483,622	86,048,162	85,886	114	5,037,243	97.81	24.07	7.99	80.04	1.00	64,276	50
GAM_NGS ((1+2)+3)	1807	483,622	86,137,425	86,940	113	5,369,578	98.02	24.10	8.00	80.04	1.00	66,262	50
GAM_NGS (((1+2)+3)+4)	1762	484,018	87,263,515	89,705	651	20,748,279	100.92	24.45	7.46	79.62	1.02	60,249	47
GAM_NGS (((1+2)+...)+5)	1755	484,018	87,236,255	90,198	620	20,456,345	101.32	24.51	7.46	79.62	1.02	60,573	47
GAM_NGS (((1+2)+...)+6)	1732	484,018	87,152,297	91,223	591	20,589,809	101.31	24.63	7.42	79.57	1.02	61,643	47
GAM_NGS (((1+2)+...)+7)	1731	484,018	87,147,035	91,259	589	20,770,121	101.80	24.68	7.40	79.58	1.02	61,184	47
GAM_NGS (((1+2)+...)+8)	1742	484,018	87,276,638	91,162	577	20,890,649	101.86	25.18	7.41	79.56	1.02	60,968	47
GARM (1+2)	1528	675,898	91,934,931	104,633	267	15,806,190	97.48	27.97	1.37	78.98	1.08	83,375	46
GARM ((1+2)+3)	11	264,708	1,042,179	144,055	2	209,647	78.68	22.18	3.74	0.93	1.05	NA	2
GARM (((1+2)+3)+4)	6	264,725	587,151	145,239	1	72,910	120.13	29.78	5.28	0.55	1.00	NA	1
GARM (((1+2)+...)+5)	5	265,528	506,174	265,528	2	338,439	132.87	30.41	3.56	0.47	1.00	NA	1
GARM (((1+2)+...)+6)	4	61,336	95,535	61,336	1	11,608	145.65	44.01	0.00	0.09	1.00	NA	1
GARM (((1+2)+...)+7)	4	61,340	95,539	61,340	1	11,608	146.70	44.01	0.00	0.09	1.00	NA	1
GARM (((1+2)+...)+8)	10	61,349	358,528	61,349	2	23,218	175.32	59.48	0.00	0.09	3.75	NA	1
Metassembler (1+2)	3148	296,904	86,092,149	46,780	107	3,693,384	101.12	23.24	0.00	79.87	1.00	35,539	59
Metassembler ((1+2)+3)	3107	296,904	86,035,088	46,780	105	3,687,158	101.04	23.22	0.00	79.82	1.00	35,539	59
Metassembler(((1+2)+...)+4)	3107	296,904	86,035,088	46,780	105	3,687,158	101.04	23.22	0.00	79.82	1.00	35,539	59
Metassembler(((1+2)+...)+5)	3106	296,904	86,032,998	46,780	106	3,726,354	101.04	23.22	0.00	79.82	1.00	35,506	59
Metassembler (((1+2)+...)+6)	2849	296,904	83,919,773	48,129	86	3,655,931	101.27	22.81	0.00	77.89	1.00	35,299	57
Metassembler(((1+2)+...)+7)	2849	296,904	83,919,773	48,129	86	3,655,931	101.27	22.81	0.00	77.89	1.00	35,299	57
Metassembler(((1+2)+...)+8)	2833	296,904	83,887,139	48,129	86	3,655,931	101.24	22.81	0.00	77.86	1.00	35,299	57

Supplemental Table S12: Experimental results on merging more than two assemblies (scaffolds) ordered by the FRCurve score (*S. aureus*, genome size 2,903,081 bp). The table reports on quality of merged assembly compared to the two input assemblies. Notes: all reported statistics are for scaffolds; the number of mismatches/indels/Ns are per 100 Kbps; tools were ran using default parameters, unless otherwise noted; (1+2)+3 means that assembly 1 and 2 were merged first, the result of which was then merged to assembly 3

Reconciliation Tool or Input	Contigs (#)	Largest (bp)	Size (bp)	N50 (bp)	Misassembly (#)	Misassembly Length (bp)	Mismatches (#)	Indels (#)	N's (#)	Genome covered (%)	Duplication ratio	NGA50 (bp)	Genes (%)
Input 1 (MSR-CA)	13	2,411,914	2,871,405	2,411,914	49	2,806,230	20.90	3.75	360.56	98.23	1.01	220,020	96
Input 2 (ALLPATHS-LG)	11	1,435,559	2,879,481	1,091,731	0	0	3.97	2.51	345.31	98.86	1.00	1,082,616	96
Input 3 (BAMBUS2)	16	1,426,293	2,862,465	1,083,792	5	2,682,283	1.48	7.86	1020.27	97.72	1.01	675,931	96
Input 4 (SGA)	299	286,534	3,051,005	149,421	3	113,622	1.09	11.47	9852.72	94.87	1.11	134,849	82
Input 5 (Velvet)	26	989,718	2,860,883	762,333	38	2,518,079	14.78	2.92	618.27	97.90	1.01	142,399	96
Input 6 (SOAPdenovo)	64	518,710	2,902,967	331,598	32	2,348,756	24.23	2.76	167.31	98.55	1.01	172,575	96
Input 7 (ABySS)	206	130,192	3,692,703	27,695	10	178,901	12.22	1.09	1520.97	97.54	1.30	31,703	77
CISA (1+2)	10	2,411,914	2,865,036	2,411,914	47	2,857,360	20.87	3.71	308.93	98.39	1.00	220,020	96
CISA (1+2+3)	14	1,796,233	2,884,041	1,796,233	47	2,856,673	20.72	3.79	283.08	99.07	1.00	220,020	96
CISA (1+2+3+4)	5	2,411,914	3,005,759	2,411,914	33	2,411,914	22.26	3.16	2186.34	82.94	1.25	220,020	64
CISA (1+2+...+5)	5	2,411,914	3,005,759	2,411,914	33	2,411,914	22.26	3.16	2186.34	82.94	1.25	220,020	64
CISA (1+2+...+6)	5	2,411,914	3,005,759	2,411,914	33	2,411,914	22.26	3.16	2186.34	82.94	1.25	220,020	64
CISA (1+2+...+7)	5	2,411,914	3,005,759	2,411,914	33	2,411,914	22.26	3.16	2186.34	82.94	1.25	220,020	64
GAA (1+2)						Did not produce an assembly file							
GAM_NGS(1+2)	13	2,411,914	2,871,405	2,411,914	49	2,806,230	20.90	3.75	360.56	98.23	1.01	220,020	96
GAM_NGS((1+2)+3)	13	2,411,914	2,871,405	2,411,914	49	2,806,230	20.90	3.75	360.56	98.23	1.01	220,020	96
GAM_NGS(((1+2)+3)+4)	13	2,411,914	2,871,405	2,411,914	49	2,806,230	20.90	3.75	360.56	98.23	1.01	220,020	96
GAM_NGS(((1+2)+...)+5)	13	2,411,914	2,871,405	2,411,914	49	2,806,230	20.90	3.75	360.56	98.23	1.01	220,020	96
GAM_NGS(((1+2)+...)+6)	13	2,411,914	2,871,405	2,411,914	49	2,806,230	20.90	3.75	360.56	98.23	1.01	220,020	96
GAM_NGS(((1+2)+...)+7)	13	2,411,914	2,871,405	2,411,914	49	2,806,230	20.90	3.75	360.56	98.23	1.01	220,020	96
GARM (1+2)	16	833,732	3,469,519	625,562	7	2,559,729	14.53	3.69	0.09	93.38	1.28	325,955	65
GARM ((1+2)+3)	16	833,738	3,554,698	625,563	8	2,524,523	11.80	3.92	0.23	93.11	1.30	330,720	64
GARM (((1+2)+3)+4)	1	634,995	634,995	634,995	4	634,995	14.10	4.59	1.42	21.74	1.01	NA	23
GARM (((1+2)+...)+5)	2	625,576	634,987	625,576	3	634,987	17.90	5.07	0.00	21.74	1.01	NA	22
GARM (((1+2)+...)+6)	11	316,522	1,117,006	152,795	10	794,721	29.53	2.44	0.00	38.15	1.01	NA	39
GARM (((1+2)+...)+7)	79	316,549	2,768,200	239,944	24	1,756,442	31.13	1.72	4.84	43.93	2.17	152,817	16
Metassembler(1+2)	6	2,411,900	2,864,554	2,411,900	41	2,747,562	21.29	4.03	326.75	98.19	1.01	254,304	96
Metassembler((1+2)+3)	5	2,411,900	2,863,848	2,411,900	41	2,747,562	21.12	4.04	326.83	98.17	1.01	254,304	96
Metassembler(((1+2)+3)+4)	5	2,412,269	2,864,113	2,412,269	38	2,747,776	20.93	4.04	428.40	98.08	1.01	254,235	96
Metassembler(((1+2)+...)+5)	5	2,412,191	2,864,011	2,412,191	38	2,747,674	21.07	4.07	413.83	98.10	1.01	254,235	96
Metassembler(((1+2)+...)+6)	5	2,412,371	2,864,191	2,412,371	38	2,747,854	21.34	4.11	385.34	98.13	1.01	254,869	96
Metassembler(((1+2)+...)+7)	5	2,412,557	2,869,873	2,412,557	37	2,748,040	21.34	4.11	383.64	98.14	1.01	257,812	96
MIX (1+2)	8	3,844,359	5,658,879	3,844,359	54	5,596,862	14.81	3.91	338.18	98.60	1.98	1,077,439	59
MIX (1+2+3)	8	3,844,359	6,742,126	3,844,359	55	6,680,654	14.70	4.61	498.33	98.66	2.36	1,077,439	59
MIX (1+2+3+4)	8	3,844,359	6,798,280	3,844,359	56	6,795,960	14.77	4.61	597.20	98.66	2.38	1,077,439	57
MIX (1+2+...+5)	8	3,844,359	6,856,232	3,844,359	57	6,853,912	14.59	4.68	592.54	98.66	2.40	1,077,568	57
MIX (1+2+...+6)	8	3,846,299	6,858,172	3,846,299	59	6,855,852	14.77	4.61	592.34	98.67	2.40	1,077,568	57
MIX (1+2+...+7)	10	2,740,531	4,120,225	2,740,531	50	4,060,521	19.68	5.51	713.87	96.97	1.47	393,556	52
ZORRO (1+2)	124	178,394	2,999,234	57,412	4	35,916	8.77	3.66	490.53	98.93	1.04	60,028	92
ZORRO ((1+2)+3)	180	140,437	2,958,837	37,759	7	69,094	6.15	2.73	1241.13	98.56	1.02	37,759	93
ZORRO (((1+2)+3)+4)	799	48,229	3,196,025	10,028	4	11,211	4.59	1.94	9394.80	97.53	1.02	11,005	86
ZORRO (((1+2)+...)+5)	427	142,720	3,249,732	36,869	6	7464	13.11	2.24	9421.15	98.28	1.03	41,143	93
ZORRO (((1+2)+...)+6)	375	212,768	3,428,465	54,406	21	540,955	26.36	3.76	8971.77	99.14	1.08	62,734	90

Supplemental Table S13: Experimental results on merging more than two assemblies (scaffolds) ordered by the FRCurve score (*R. sphaeroides*, genome size 4,603,060 bp). The table reports on quality of merged assembly compared to the two input assemblies. Notes: all reported statistics are for scaffolds; the number of mismatches/indels/Ns are per 100 Kbps; tools were ran using default parameters, unless otherwise noted; (1+2)+3 means that assembly 1 and 2 were merged first, the result of which was then merged to assembly 3

Reconciliation Tool or Input	Contigs (#)	Largest (bp)	Size (bp)	N50 (bp)	Misass (#)	Misassembly Length (bp)	Mismatches (#)	Indels (#)	N's (#)	Genome covered (%)	Duplic ratio	NGA50 (bp)	Genes (%)
Input 1 (MSR-CA)	36	2,975,504	4,495,726	2,975,504	29	3,765,235	20.10	11.36	725.76	96.22	1.01	535,459	92
Input 2 (ALLPATHS-LG)	33	3,192,334	4,608,763	3,192,334	15	4,447,871	5.91	6.79	467.31	99.24	1.01	928,821	95
Input 3 (CABOG)	130	1,352,519	4,259,679	245,073	20	2,427,350	28.19	7.91	505.84	91.95	1.01	55,312	88
Input 4 (BAMBUS2)	92	2,438,508	4,428,612	2,438,508	11	2,971,543	5.95	6.20	1288.01	94.94	1.01	343,187	90
Input 5 (SOAPdenovo)	76	1,154,134	4,579,801	660,164	13	1,927,959	21.30	9.81	228.42	98.73	1.01	539,770	92
Input 6 (Velvet)	115	770,958	4,572,546	353,027	41	1,840,832	8.64	10.36	1898.40	97.43	1.01	223,489	93
Input 7 (SGA)	1208	148,756	5,328,387	44,205	3	31,764	5.86	7.22	21,499.94	90.77	1.26	17,695	77
Input 8 (ABySS)	1352	87,855	4,968,921	8036	85	1,012,703	23.38	8.92	2302.47	94.21	1.14	7136	76
CISA (1+2)	8	3,192,334	4,962,823	3,192,334	20	4,818,520	10.09	7.26	559.52	95.79	1.13	928,821	82
CISA (1+2+3)	5	2,952,884	5,053,724	2,952,884	12	3,946,023	8.23	7.40	397.67	88.68	1.24	865,102	65
CISA (1+2+3+4)	5	2,952,884	5,053,724	2,952,884	12	3,946,023	8.23	7.40	397.67	88.68	1.24	865,102	65
CISA (1+2+...+5)	5	2,952,884	5,053,724	2,952,884	12	3,946,023	8.23	7.40	397.67	88.68	1.24	865,102	65
CISA (1+2+...+6)	5	2,952,991	5,054,054	2,952,991	13	4,184,063	8.23	7.37	397.64	88.68	1.24	865,102	65
CISA (1+2+...+7)	5	2,952,991	5,054,054	2,952,991	13	4,184,063	8.23	7.37	397.64	88.68	1.24	865,102	65
CISA (1+2+...+8)	5	2,953,019	5,054,305	2,953,019	13	4,184,091	8.23	7.40	397.62	88.68	1.24	865,325	65
GAA (1+2)						Did not produce an assembly file							
GAM_NGS(1+2)	36	2,975,504	4,495,726	2,975,504	29	3,765,235	20.10	11.36	725.76	96.22	1.01	535,459	92
GAM_NGS(1+2+3)	36	2,975,504	4,495,726	2,975,504	29	3,765,235	20.10	11.36	725.76	96.22	1.01	535,459	92
GAM_NGS(((1+2)+3)+4)	36	2,975,504	4,495,885	2,975,504	29	3,765,235	19.46	11.24	725.73	96.22	1.01	535,459	92
GAM_NGS(((1+2)+...)+5)	35	2,975,504	4,495,773	2,975,504	29	3,765,235	19.53	11.24	725.75	96.22	1.01	535,459	92
GAM_NGS(((1+2)+...)+6)	35	2,975,504	4,495,773	2,975,504	29	3,765,235	19.53	11.24	725.75	96.22	1.01	535,459	92
GAM_NGS(((1+2)+...)+7)	35	2,975,504	4,495,773	2,975,504	29	3,765,235	19.53	11.24	725.75	96.22	1.01	535,459	92
GAM_NGS(((1+2)+...)+8)	35	2,975,504	4,495,773	2,975,504	29	3,765,235	19.53	11.24	725.75	96.22	1.01	535,459	92
GARM (1+2)	63	287,164	4,330,697	110,067	12	754,372	10.33	7.41	1.18	90.85	1.02	104,539	86
GARM ((1+2)+3)	6	222,843	682,218	191,001	2	191,001	9.26	6.83	3.52	14.31	1.04	NA	14
Metassembler(1+2)	18	2,975,287	4,485,906	2,975,287	23	3,767,604	19.45	10.94	623.84	96.53	1.01	542,318	92
Metassembler(1+2+3)	12	2,975,287	4,330,089	2,975,287	18	3,610,551	18.22	11.16	598.37	93.23	1.01	542,318	89
Metassembler(((1+2)+3)+4)	11	2,975,287	4,329,474	2,975,287	18	3,610,551	18.22	11.16	598.46	93.22	1.01	542,318	89
Metassembler(((1+2)+...)+5)	11	2,974,171	4,345,683	2,974,171	19	3,627,111	17.67	11.17	589.73	93.56	1.01	542,141	90
Metassembler(((1+2)+...)+6)	11	2,974,143	4,345,655	2,974,143	19	3,627,083	17.67	11.10	588.10	93.56	1.01	542,141	90
Metassembler(((1+2)+...)+7)	11	2,974,043	4,345,553	2,974,043	19	3,627,064	18.32	11.57	849.21	93.32	1.01	541,086	90
Metassembler(((1+2)+...)+8)	11	2,973,698	4,344,414	2,973,698	17	3,626,594	18.28	11.55	848.51	93.29	1.01	541,086	90
MIX (1+2)	17	10,738,254	11,354,250	10,738,254	46	11,324,751	11.33	5.84	550.11	96.65	2.55	1,443,594	91
MIX (1+2+3)	13	10,738,254	11,886,482	10,738,254	47	11,832,525	19.84	6.14	568.05	96.92	2.67	1,443,594	78
MIX (1+2+3+4)	16	10,738,254	12,576,249	10,738,254	55	12,334,005	20.32	6.35	566.99	97.50	2.80	1,443,594	72
MIX (1+2+...+5)	20	6,575,227	9,499,456	6,575,227	46	9,190,236	19.93	6.41	621.73	97.21	2.12	1,208,391	70
MIX (1+2+...+6)	24	6,575,227	10,129,718	6,575,227	56	9,100,171	19.91	6.62	825.45	97.42	2.26	1,208,391	59
MIX (1+2+...+7)	27	6,575,227	10,240,091	6,575,227	60	9,181,273	20.12	6.68	1035.00	97.60	2.28	1,208,391	58
MIX (1+2+...+8)	31	6,575,227	10,345,176	6,575,227	76	9,214,371	20.75	6.81	1891.69	98.84	2.28	1,208,391	56
ZORRO (1+2)	213	223,172	4,709,234	60,544	12	281,886	19.37	11.67	868.36	99.27	1.02	60,489	92
ZORRO (1+2+3)	209	159,412	4,760,417	62,222	15	425,102	22.94	11.10	1204.83	99.26	1.03	60,687	92
ZORRO (((1+2)+3)+4)	543	90,880	4,735,152	17,051	15	256,065	18.12	7.99	2213.93	97.38	1.04	17,579	88
ZORRO (((1+2)+...)+5)	529	88,610	4,844,535	18,041	21	252,097	25.08	8.57	2262.12	98.90	1.04	18,736	88
ZORRO (((1+2)+...)+6)	493	158,620	4,904,571	26,231	19	237,325	21.13	10.03	3836.77	98.80	1.04	26,811	90
ZORRO (((1+2)+...)+7)	2207	87,934	5,979,889	3125	14	141,079	20.06	6.03	21,777.66	97.91	1.04	4501	80

Supplemental Table S14: Experimental results on merging more than two assemblies (scaffolds) ordered by the FRCurve score (*Hg_chr14*, genome size 107,349,540 bp). The table reports on quality of merged assembly compared to the two input assemblies. Notes: all reported statistics are for contigs; the number of mismatches/indels/Ns are per 100 Kbps; tools were ran using default parameters, unless otherwise noted; (1+2)+3 means that assembly 1 and 2 were merged first, the result of which was then merged to assembly 3

Reconciliation Tool or Input	Contigs (#)	Largest (bp)	Size (bp)	N50 (bp)	Misassembly (#)	Misassembly Length (bp)	Mismatches (#)	Indels (#)	N's (#)	Genome covered (%)	Duplicat ratio	NGA50 (bp)	Genes (%)
Input 1 (CABOG)	474	2,260,562	86,481,568	401,279	269	43,205,905	101.10	24.68	267.20	80.01	1.01	215,699	29
Input 2 (ALLPATHS-LG)	174	81,646,936	87,646,728	81,646,936	455	87,219,645	66.85	22.87	3734.63	78.51	1.04	397,351	14
Input 3 (ABySS)	31,582	30,053	67,724,594	3355	37	237,738	84.67	9.37	859.44	61.61	1.02	1339	83
Input 4 (SOAPdenovo)	7264	1,849,511	100,880,746	381,286	8171	89,396,625	152.68	24.52	10,166.39	77.44	1.20	17,851	31
Input 5 (MSR-CA)	1056	4,208,965	89,531,681	893,428	6938	86,537,566	226.67	41.77	6810.92	75.68	1.10	31,378	23
Input 6 (BAMBUS)	847	2,671,981	78,283,056	372,757	5010	74,197,764	102.69	21.95	13,247.26	62.59	1.16	13,131	19
Input 7 (SGA)	9586	551,622	88,557,645	82,616	170	8,811,457	87.77	18.34	14,499.38	70.47	1.16	35,317	34
Input 8 (Velvet)	1463	4,628,722	138,771,192	854,836	12,836	99,579,307	107.32	28.47	45,797.46	68.55	1.57	3334	28
GAA (1+2)						Did not produce an assembly file							
GAM_NGS (1+2)	473	2,260,562	86,479,699	401,279	269	43,205,905	101.10	24.68	267.22	80.01	1.01	215,699	29
GAM_NGS ((1+2)+3)	466	2,260,562	87,363,451	409,989	271	43,745,348	101.36	24.71	272.57	80.01	1.02	226,183	NA
GAM_NGS (((1+2)+3)+4)	462	2,260,562	87,370,724	409,989	276	43,994,460	101.39	24.67	284.02	80.00	1.02	226,183	29
GAM_NGS (((1+2)+. . .)+5)	473	2,260,562	86,479,699	401,279	269	43,205,905	101.10	24.68	267.22	80.01	1.01	215,699	29
GAM_NGS (((1+2)+. . .)+6)	455	2,260,562	87,576,196	409,989	331	47,081,846	101.52	24.68	734.37	79.81	1.02	223,685	28
GAM_NGS (((1+2)+. . .)+7)	455	2,260,562	87,577,295	409,989	331	47,081,846	101.51	24.68	737.15	79.81	1.02	223,685	28
GARM (1+2)	1528	675,898	91,934,931	104,633	267	15,806,190	97.48	27.97	1.37	78.98	1.08	83,375	0
GARM ((1+2)+3)	11	264,708	1,042,179	144,055	2	209,647	78.68	22.18	3.74	0.93	1.05	NA	0
GARM (((1+2)+3)+4)	6	264,725	587,151	145,239	1	72,910	120.13	29.78	5.28	0.55	1.00	NA	0
GARM (((1+2)+. . .)+5)	5	265,528	506,174	265,528	2	338,439	132.87	30.41	3.56	0.47	1.00	NA	0
GARM (((1+2)+. . .)+6)	4	61,336	95,535	61,336	1	11,608	145.65	44.01	0.00	0.09	1.00	NA	0
GARM (((1+2)+. . .)+7)	4	61,340	95,539	61,340	1	11,608	146.70	44.01	0.00	0.09	1.00	NA	0
GARM (((1+2)+. . .)+8)	10	61,349	358,528	61,349	2	23,218	175.32	59.48	0.00	0.09	3.75	NA	0
Metassembler (1+2)	465	2,260,562	86,454,843	401,279	269	43,205,905	100.93	24.64	267.28	79.99	1.01	215,699	29
Metassembler ((1+2)+3)	461	2,260,562	86,446,782	401,279	269	43,205,905	100.92	24.64	267.31	79.99	1.01	215,699	29
Metassembler(((1+2)+. . .)+4)	461	2,260,562	86,446,782	401,279	269	43,205,905	100.92	24.64	267.31	79.99	1.01	215,699	29
Metassembler(((1+2)+. . .)+5)	461	2,260,562	86,446,782	401,279	269	43,205,905	100.92	24.64	267.31	79.99	1.01	215,699	29
Metassembler (((1+2)+. . .)+6)	457	2,260,562	86,427,089	401,279	269	43,205,905	100.93	24.64	266.36	79.97	1.01	215,699	29
Metassembler(((1+2)+. . .)+7)	457	2,260,562	86,427,089	401,279	269	43,205,905	100.93	24.64	266.36	79.97	1.01	215,699	29
Metassembler(((1+2)+. . .)+8)	456	2,260,562	86,419,639	401,279	269	43,205,905	100.93	24.64	266.38	79.97	1.01	215,699	29

Supplemental Table S15: Experimental results on merging more than two assemblies (as contigs) with an alternative ordering (*S. aureus*, genome size 2,903,081 bp). The table reports on quality of merged assembly compared to the two input assemblies. Notes: Statistics reported are for contigs; the number of mismatches/indels/Ns are per 100 Kbps; tools were ran using default parameters, unless otherwise noted

Reconciliation Tool	Contigs (#)	Largest (bp)	Size (bp)	N50 (bp)	Misassembly (#)	Misassembly Length (bp)	Mismatches (#)	Indels (#)	N's (#)	Genome covered (%)	Duplication ratio	NGA50 (bp)	Genes (%)
Input 1 (SGA)	985	16,870	2,748,664	4178	1	2431	1.02	0.11	0.00	94.44	1.00	4005	82
Input 2 (ABySS)	247	125,049	3,631,245	25,084	5	22,399	12.39	0.85	7.79	97.27	1.29	29,198	78
Input 3 (ALLPATHS-LG)	59	234,488	2,869,581	96,740	1	89,634	1.57	0.73	1.50	98.83	1.00	96,740	96
Input 4 (MSR-CA)	89	139,438	2,860,132	59,152	20	641,173	20.67	1.82	0.00	98.17	1.00	55,068	95
Input 5 (Velvet)	128	169,214	2,837,036	52,792	9	284,379	13.51	1.69	0.00	97.73	1.00	48,149	97
Input 6 (SOAPdenovo)	70	518,710	2,897,432	288,184	31	2,027,905	23.00	2.45	0.07	98.55	1.01	150,794	96
Input 7 (BAMBUS2)	106	158,330	2,832,623	50,192	1	9411	1.41	6.95	0.35	97.66	1.00	50,192	95
CISA (1+2)	183	125,049	2,775,035	25,585	6	44,257	14.86	1.23	4.58	95.07	1.00	23,610	93
CISA (1+2+3)	52	245,875	2,877,782	131,781	2	251,080	5.15	1.08	4.66	98.97	1.00	131,781	96
CISA (1+2+3+4)	27	481,008	2,867,994	114,938	10	1,204,080	8.73	1.09	1.26	95.04	1.04	114,873	90
CISA (1+2+...+5)	22	390,086	2,730,406	201,618	8	1,101,155	6.87	1.02	1.28	90.78	1.04	114,873	86
CISA (1+2+...+6)	18	521,399	3,033,607	288,204	28	2,329,572	16.70	2.18	0.86	94.85	1.10	179,344	84
CISA (1+2+...+7)	18	521,399	3,033,607	288,204	28	2,329,572	16.70	2.18	0.86	94.85	1.10	179,344	84
GAA (1+2)	1232	125,049	6,379,909	10,535	6	24,830	11.04	0.70	4.44	97.93	2.24	29,198	27
GAA ((1+2)+3)	1290	234,488	9,246,594	24,526	7	114,464	5.06	0.87	3.53	99.30	3.21	107,125	27
GAA (((1+2)+3)+4)	1378	234,488	12,105,447	32,180	26	754,358	11.24	1.66	2.69	99.63	4.19	123,414	27
GAA ((((1+2)+3)+4)+5)	1502	234,488	14,931,998	35,275	35	1,038,737	14.23	2.63	2.18	99.73	5.16	132,320	27
GAA ((((1+2)+...)+5)+6)	1570	518,710	17,823,454	46,565	66	3,066,642	18.36	2.59	1.84	99.80	6.16	200,247	27
GAA ((((1+2)+...)+6)+7)	1665	518,710	20,622,604	48,304	67	3,076,053	18.02	2.80	1.64	99.80	7.12	200,247	27
GAM_NGS (1+2)	545	77,430	2,784,898	10,328	1	2431	2.19	0.36	0.36	95.78	1.00	9371	89
GAM_NGS ((1+2)+3)	193	234,571	2,835,523	69,769	1	2431	2.51	0.60	1.23	97.61	1.00	68,648	92
GAM_NGS (((1+2)+3)+4)	120	386,769	2,846,597	97,655	2	386,651	4.43	0.60	1.23	98.02	1.00	97,655	93
GAM_NGS ((((1+2)+3)+4)+5)	89	386,769	2,852,151	98,193	3	484,844	6.14	0.70	1.23	98.22	1.00	97,655	94
GAM_NGS ((((1+2)+...)+5)+6)	65	475,528	2,857,733	172,603	3	576,152	6.97	0.74	1.22	98.37	1.00	149,870	94
GAM_NGS ((((1+2)+...)+6)+7)	50	493,282	2,858,546	216,471	3	576,152	7.04	0.88	1.29	98.41	1.00	172,596	94
GARM (1+2)	96	125,477	1,486,962	28,882	6	32,684	24.19	4.73	8.14	30.61	1.67	3115	20
GARM ((1+2)+3)	26	751,197	2,697,219	234,533	0	0	8.08	3.30	0.15	92.91	1.00	200,248	91
GARM (((1+2)+3)+4)	27	833,735	3,132,585	430,275	14	1,960,878	19.30	5.00	0.00	98.54	1.10	331,026	88
GARM ((((1+2)+3)+4)+5)	28	885,261	5,439,174	480,862	21	4,289,841	19.85	5.19	0.00	98.03	1.91	833,528	19
GARM ((((1+2)+...)+5)+6)	12	885,261	2,983,960	885,261	18	2,810,529	24.05	3.26	0.00	56.00	1.84	833,529	22
GARM ((((1+2)+...)+6)+7)	53	885,273	3,346,358	158,330	14	1,715,106	14.38	5.51	0.09	95.59	1.21	195,100	75
Metassembler (1+2)	401	77,430	2,825,605	19,008	3	11,572	5.42	0.54	1.91	96.54	1.01	18,208	91
Metassembler ((1+2)+3)	139	172,102	2,855,739	48,865	3	36,237	4.19	0.92	2.77	97.86	1.01	48,862	94
Metassembler (((1+2)+3)+4)	115	218,147	2,847,058	56,518	4	61,375	7.20	1.20	2.70	97.58	1.01	53,149	94
Metassembler ((((1+2)+3)+4)+5)	114	218,147	2,847,492	56,518	5	167,844	8.12	1.24	2.70	97.59	1.01	53,149	94
Metassembler ((((1+2)+...)+5)+6)	102	222,905	2,857,903	64,023	12	641,767	13.13	1.44	2.76	97.86	1.01	60,945	94
Metassembler ((((1+2)+...)+6)+7)	101	222,905	2,857,921	64,023	12	641,767	13.13	1.48	2.76	97.86	1.01	60,945	94
MIX (1+2)	156	125,049	2,400,202	27,695	3	12,322	15.91	1.11	6.83	80.34	1.03	23,082	79
MIX (1+2+3)	44	234,488	1,946,016	97,697	1	89,634	2.00	0.82	1.90	67.03	1.00	48,304	64
MIX (1+2+3+4)	19	200,247	658,945	69,391	0	0	3.34	0.91	1.97	22.69	1.00	NA	23
MIX (1+2+...+5)	18	200,247	685,973	66,230	0	0	3.06	0.73	1.75	23.62	1.00	NA	24
MIX (1+2+...+6)	22	518,710	1,614,188	331,598	24	1,345,664	25.45	2.92	0.00	55.35	1.01	46,390	55
MIX (1+2+...+7)	22	518,710	1,283,250	316,522	16	1,014,066	26.86	3.13	0.00	43.99	1.01	NA	44
ZORRO (1+2)	Produced an empty assembly file												

Supplemental Table S16: Experimental results on merging more than two assemblies (as contigs) with an alternative ordering (*R. sphaeroides*, genome size 4,603,060 bp). The table reports on quality of merged assembly compared to the two input assemblies. Notes: Statistics reported are for contigs; the number of mismatches/indels/Ns are per 100 Kbps; tools were ran using default parameters, unless otherwise noted; (1+2)+3 means that assembly 1 and 2 were merged first, the result of which was then merged to assembly 3

Reconciliation Tool	Contigs (#)	Largest (bp)	Size (bp)	N50 (bp)	Misassembly (#)	Misassembly Length (bp)	Mismatches (#)	Indels (#)	N's (#)	Genome covered (%)	Duplication ratio	NGA50 (bp)	Genes (%)
Input 1 (SGA)	2173	29,520	4,188,432	2530	1	4048	5.70	2.47	0.00	90.70	1.00	2280	78
Input 2 (CABOG)	318	88,519	4,236,663	22,044	11	276,929	29.07	5.48	0.00	91.91	1.00	19,076	87
Input 3 (Velvet)	482	60,714	4,470,215	16,033	6	127,247	8.58	4.06	0.00	96.94	1.00	15,439	92
Input 4 (MSR-CA)	377	83,726	4,458,952	23,575	18	151,790	23.43	4.90	0.00	96.14	1.01	21,236	89
Input 5 (ALLPATHS-LG)	203	106,467	4,587,354	42,455	10	404,185	6.33	4.77	2.79	99.20	1.00	41,487	93
Input 6 (ABySS)	1509	54,734	4,830,769	5562	85	866,218	22.76	5.84	2.32	93.75	1.12	5303	76
Input 7 (BAMBUS2)	170	279,125	4,369,357	97,331	4	123,417	5.82	5.84	0.00	94.89	1.00	93,198	90
Input 8 (SOAPdenovo)	114	376,585	4,569,340	131,681	11	633,163	21.28	9.51	0.00	98.72	1.01	129,613	92
CISA (1+2)	361	88,509	4,227,470	20,303	11	286,366	29.00	5.27	0.00	91.84	1.00	17,590	86
CISA (1+2+3)	351	88,516	4,550,294	22,683	12	311,963	29.42	5.28	0.00	98.28	1.01	21,435	92
CISA (1+2+3+4)	207	88,519	4,632,650	32,548	13	277,288	35.31	6.57	0.00	97.21	1.04	31,768	88
CISA (1+2+...+5)	145	119,461	4,716,607	50,937	20	563,586	23.50	5.56	1.78	98.08	1.05	47,859	89
CISA (1+2+...+6)	120	119,461	4,623,276	51,710	24	714,575	23.88	6.39	1.93	95.61	1.05	50,889	87
CISA (1+2+...+7)	66	279,125	4,983,880	101,270	20	780,427	29.25	8.45	0.72	92.03	1.18	105,293	73
CISA (1+2+...+8)	38	377,567	5,009,192	154,509	18	1,224,227	32.48	10.72	0.30	89.96	1.21	154,509	68
GAA (1+2)	2489	88,519	8,422,953	6871	12	280,977	28.51	5.16	0.00	98.44	1.86	19,278	33
GAA ((1+2)+3)	2971	88,519	12,893,168	11,295	18	408,224	29.41	5.26	0.00	98.69	2.84	26,927	29
GAA (((1+2)+3)+4)	3348	88,519	17,352,120	13,897	36	560,014	35.68	5.66	0.00	99.48	3.79	36,899	30
GAA ((((1+2)+3)+4)+5)	3542	106,467	21,929,269	18,074	46	964,199	23.32	5.54	0.58	99.69	4.78	58,687	30
GAA ((((1+2)+...)+5)+6)	5032	106,467	26,727,756	14,534	131	1,830,417	24.71	6.14	0.90	99.79	5.82	58,687	33
GAA ((((1+2)+...)+6)+7)	5126	279,125	30,814,257	18,186	134	1,948,517	24.73	6.31	0.78	99.79	6.71	97,360	34
GAA (((((1+2)+...)+6)+7)+8)	5239	376,585	35,379,799	22,511	145	2,581,680	27.66	7.92	0.68	99.84	7.70	162,986	34
GAM_NGS (1+2)	772	88,314	4,386,840	15,268	8	177,696	32.62	5.36	0.00	95.30	1.00	13,419	85
GAM_NGS ((1+2)+3)	538	88,314	4,425,712	20,210	8	177,803	32.65	5.81	0.00	96.16	1.00	18,619	86
GAM_NGS (((1+2)+3)+4)	412	103,668	4,468,894	26,294	8	152,011	35.55	6.22	0.00	97.04	1.00	24,420	86
GAM_NGS ((((1+2)+3)+4)+5)	314	129,231	4,496,017	39,565	12	311,939	36.81	6.57	0.24	97.56	1.00	35,767	87
GAM_NGS ((((1+2)+...)+5)+6)	304	136,773	4,498,185	40,397	12	410,768	37.48	6.57	0.24	97.60	1.00	37,591	87
GAM_NGS ((((1+2)+...)+6)+7)	281	136,773	4,502,388	40,839	12	410,811	37.54	6.65	0.24	97.69	1.00	40,397	87
GAM_NGS ((((1+2)+...)+7)+8)	220	227,331	4,517,030	51,976	13	493,518	42.32	7.40	0.24	98.00	1.00	47,344	87
GARM (1+2)	226	88,521	2,972,274	22,754	7	218,125	39.83	7.72	0.13	64.47	1.00	11,605	62
GARM ((1+2)+3)	317	88,521	4,439,643	23,404	15	449,942	29.70	6.36	0.59	88.15	1.10	21,768	78
GARM (((1+2)+3)+4)	196	102,179	4,703,725	41,550	21	468,001	35.75	7.08	0.40	93.95	1.09	40,660	84
GARM ((((1+2)+3)+4)+5)						Did not produce assembly files							

Supplemental Table S17: Experimental results on merging more than two assemblies (as contigs) with an alternative ordering (*R. sphaeroides*, genome size 4,603,060 bp). The table reports on quality of merged assembly compared to the two input assemblies. Notes: Statistics reported are for contigs; the number of mismatches/indels/Ns are per 100 Kbps; tools were ran using default parameters, unless otherwise noted; (1+2)+3 means that assembly 1 and 2 were merged first, the result of which was then merged to assembly 3

Reconciliation Tool	Contigs (#)	Largest (bp)	Size (bp)	N50 (bp)	Misassembly (#)	Misassembly Length (bp)	Mismatches (#)	Indels (#)	N's (#)	Genome covered (%)	Duplication ratio	NGA50 (bp)	Genes (%)
Input 1 (SGA)	2173	29,520	4,188,432	2530	1	4048	5.70	2.47	0.00	90.70	1.00	2280	78
Input 2 (CABOG)	318	88,519	4,236,663	22,044	11	276,929	29.07	5.48	0.00	91.91	1.00	19,076	87
Input 3 (Velvet)	482	60,714	4,470,215	16,033	6	127,247	8.58	4.06	0.00	96.94	1.00	15,439	92
Input 4 (MSR-CA)	377	83,726	4,458,952	23,575	18	151,790	23.43	4.90	0.00	96.14	1.01	21,236	89
Input 5 (ALLPATHS-LG)	203	106,467	4,587,354	42,455	10	404,185	6.33	4.77	2.79	99.20	1.00	41,487	93
Input 6 (ABySS)	1509	54,734	4,830,769	5562	85	866,218	22.76	5.84	2.32	93.75	1.12	5303	76
Input 7 (BAMBUS2)	170	279,125	4,369,357	97,331	4	123,417	5.82	5.84	0.00	94.89	1.00	93,198	90
Input 8 (SOAPdenovo)	114	376,585	4,569,340	131,681	11	633,163	21.28	9.51	0.00	98.72	1.01	129,613	92
Metassembler (1+2)	654	85,765	4,104,684	14,248	3	98,343	17.15	4.51	0.00	89.07	1.00	13,072	84
Metassembler ((1+2)+3)	611	85,765	4,110,564	15,622	2	46,073	17.10	4.72	0.00	89.21	1.00	13,960	84
Metassembler (((1+2)+3)+4)	447	101,356	4,127,410	20,614	3	69,841	21.91	5.65	0.00	89.62	1.00	18,750	85
Metassembler ((((1+2)+3)+4)+5)	375	125,375	4,161,595	25,381	3	69,841	22.74	6.30	0.17	90.36	1.00	22,037	86
Metassembler ((((1+2)+...)+5)+6)	373	125,375	4,170,384	25,536	3	69,841	22.70	6.32	0.17	90.44	1.00	22,046	86
Metassembler ((((1+2)+...)+6)+7)	244	125,375	4,218,214	37,701	5	102,128	22.87	7.50	0.17	91.50	1.00	33,004	86
Metassembler ((((1+2)+...)+7)+8)	375	125,375	4,161,595	25,381	3	69,841	22.74	6.30	0.17	90.36	1.00	22,037	86
MIX (1+2)	311	88,519	4,130,025	21,782	9	220,445	12.80	4.68	0.00	89.61	1.00	18,331	85
MIX (1+2+3)	299	88,519	3,942,979	22,024	9	183,272	30.24	5.60	0.00	85.71	1.00	17,868	81
MIX (1+2+3+4)	190	88,519	2,425,032	22,024	7	126,788	16.66	4.41	0.00	52.67	1.00	3780	52
MIX (1+2+...+5)	146	106,467	3,028,711	40,555	4	235,868	5.28	4.79	2.05	65.78	1.00	20,380	63
MIX (1+2+...+6)	142	106,467	2,873,498	38,162	4	235,868	5.08	4.32	1.95	62.41	1.00	18,081	60
MIX (1+2+...+7)	78	279,125	2,668,747	107,963	2	31,469	6.74	7.01	0.00	57.98	1.00	28,260	56
MIX (1+2+...+8)	42	376,585	1,905,119	162,015	4	176,145	20.32	7.88	0.00	41.38	1.00	NA	40
ZORRO (1+2)	419	70,517	4,534,309	19,161	10	199,853	37.47	5.67	0.04	98.39	1.00	18,028	92
ZORRO ((1+2)+3)	Produced an empty assembly file												

Supplemental Table S18: Experimental results on merging more than two assemblies (as contigs) with an alternative ordering (*Hg_chr14*, genome size 107,349,540 bp). The table reports on quality of merged assembly compared to the two input assemblies. Notes: Reported statistics are for contigs; the number of mismatches/indels/Ns are per 100 Kbps; tools were ran using default parameters, unless otherwise noted; (1+2)+3 means that assembly 1 and 2 were merged first, the result of which was then merged to assembly 3

Reconciliation Tool	Contigs (#)	Largest (bp)	Size (bp)	N50 (bp)	Misassembly (#)	Misassembly Length (bp)	Mismatches (#)	Indels (#)	N's (#)	Genome covered (%)	Duplication ratio	NGA50 (bp)	Genes (%)
Input 1 (ABySS)	32,050	30,053	67,074,140	3182	24	128,244	84.48	9.20	1.31	61.54	1.01	1319	84
Input 2 (SGA)	33,695	30,350	75,492,807	3317	107	249,973	87.51	12.57	0.00	69.89	1.01	1945	89
Input 3 (ALLPATHS-LG)	4469	240,773	84,416,102	38,359	109	1,384,277	67.71	21.79	54.60	78.48	1.00	27,772	63
Input 4 (CABOG)	3233	296,904	86,189,919	46,699	108	3,694,326	101.52	23.29	0.00	79.94	1.00	35,539	59
Input 5 (MSR-CA)	25,022	53,925	81,485,144	5470	2038	6,589,712	220.87	24.66	0.00	74.21	1.02	3427	83
Input 6 (Velvet)	32,842	27,872	70,575,215	3087	232	602,910	104.51	21.41	0.00	65.33	1.00	1580	82
Input 7 (SOAPdenovo)	15,028	147,494	90,398,734	16,179	6329	43,713,769	152.34	24.26	0.02	77.30	1.09	8155	64
Input 8 (BAMBUS)	12,396	736,657	67,814,016	8500	2973	12,211,265	104.23	22.18	0.01	62.52	1.01	3218	62
GAA (1+2)	65,737	30,350	142,552,348	3255	131	378,217	90.38	13.70	0.62	70.52	1.88	4336	84
GAA ((1+2)+3)	70,204	240,773	226,966,664	6119	240	1,762,494	72.47	22.05	20.70	79.33	2.67	27,960	79
GAA (((1+2)+3)+4)	73,416	296,904	313,139,760	12,780	348	5,456,915	95.13	23.86	15.00	80.66	3.62	62,647	78
GAA ((((1+2)+3)+4)+5)	98,421	296,904	394,599,185	9054	2383	12,043,368	99.30	24.45	11.90	80.80	4.55	62,647	81
GAA ((((1+2)+. . .)+5)+6)	131,262	296,904	465,173,620	6993	2615	12,646,278	99.66	24.47	10.10	80.87	5.35	62,647	81
GAA ((((1+2)+. . .)+6)+7)	140,219	296,904	548,769,603	8483	8920	56,321,449	106.13	25.07	8.56	80.98	6.31	63,107	83
GAA ((((1+2)+. . .)+7)+8)	152,492	736,657	616,390,960	8492	11,893	68,532,714	105.99	25.07	7.63	81.00	7.08	63,107	84
GAM_NGS (1+2)	28,297	52,608	69,334,740	3755	30	163,762	85.20	9.95	1.18	63.74	1.01	1755	83
GAM_NGS ((1+2)+3)	9736	215,503	79,581,806	34,673	45	744,030	85.23	18.38	10.52	73.52	1.01	15,730	60
GAM_NGS (((1+2)+3)+4)	4481	397,751	83,219,962	69,951	74	3,263,338	92.18	21.68	9.94	77.00	1.01	46,651	52
GAM_NGS ((((1+2)+3)+4)+5)	4192	397,751	83,368,507	70,604	82	3,549,998	94.03	22.02	9.90	77.17	1.01	46,964	51
GAM_NGS ((((1+2)+. . .)+5)+6)	4138	397,751	83,403,680	70,931	86	3,889,938	94.63	22.48	9.86	77.19	1.01	47,047	51
GAM_NGS ((((1+2)+. . .)+6)+7)	3697	397,751	84,176,751	75,581	866	19,610,702	99.41	23.13	9.31	77.42	1.01	43,297	47
GAM_NGS ((((1+2)+. . .)+7)+8)	3554	397,751	84,227,364	77,356	807	19,477,192	99.48	23.26	9.26	77.45	1.01	44,168	47
GARM (1+2)	20,866	55,072	70,026,320	5197	81	340,195	88.71	13.87	0.19	64.59	1.00	2420	77
GARM ((1+2)+3)	3352	430,290	84,628,192	51,097	258	6,861,404	90.56	34.38	0.62	78.45	1.00	35,794	57
GARM (((1+2)+3)+4)	1626	621,599	98,329,510	109,228	261	16,196,216	100.24	25.81	0.04	75.80	1.20	93,129	46
GARM ((((1+2)+3)+4)+5)	3519	700,715	152,827,973	125,791	799	38,358,283	143.98	29.84	0.02	77.02	1.84	145,176	46
GARM ((((1+2)+. . .)+5)+6)	5091	814,188	404,136,067	169,384	1250	108,723,014	143.35	33.86	0.00	74.66	5.04	314,774	43
GARM ((((1+2)+. . .)+6)+7)	1	950	950	950	0	0	105.26	0.00	0.00	0.00	1.00	NA	0
GARM ((((1+2)+. . .)+7)+8)						Did not produce assembly files							
Metassembler (1+2)	31,853	30,053	66,646,160	3174	22	124,338	84.11	9.18	1.28	61.48	1.01	1293	77
Metassembler ((1+2)+3)	31,474	30,053	66,230,547	3193	20	117,582	83.51	9.13	1.26	61.18	1.01	1280	76
Metassembler (((1+2)+3)+4)	31,392	30,053	66,140,593	3198	20	117,582	83.46	9.14	1.16	61.10	1.01	1276	76
Metassembler ((((1+2)+3)+4)+5)	31,317	30,053	66,080,716	3202	20	117,582	83.42	9.13	1.16	61.05	1.01	1275	76
Metassembler ((((1+2)+. . .)+5)+6)	31,189	30,053	65,936,787	3206	20	117,582	83.15	9.08	1.16	60.94	1.01	1271	75
Metassembler ((((1+2)+. . .)+6)+7)	31,187	30,053	65,935,548	3208	20	117,582	83.14	9.08	1.16	60.94	1.01	1271	75
Metassembler ((((1+2)+. . .)+7)+8)	24,682	30,053	56,850,690	3565	19	112,449	84.52	9.19	1.26	52.57	1.01	777	64

Supplemental Table S19: Experiments on the *Bombus impatiens* assemblies. Notes: all reported statistics are for contigs; tools were ran using default parameters, unless otherwise noted; the E-Size is defined as the expected scaffold size of any arbitrary location in the reference genome.

Reconciliation Tool or Input	Contigs #	N50 (bp)	E-Size (bp)
Input 1 (CABOG)	18,918	23,515	34,227.94
Input 2 (MSR-CA)	18,501	32,431	46,890.24
GAA	Did not produce an assembly file		
GAM_NGS	10,129	52,123	77,240.76
GARM (ctg_scf)	10,572	70,577	98,189.44
Metassembler	17,694	25,317	36,774.11
Input 1 (ABySS)	35,112	14,383	20,904.98
Input 2 (SOAPdenovo)	11,556	57,117	78,228.65
GAA	46,668	63,941	99,133.63
GAM_NGS	Did not produce an assembly file		
GARM (ctg_scf)	9477	64,172	86,881.27
Metassembler	34,149	13,842	20,386.78
Input 1 (SOAPdenovo)	11,556	57,117	78,228.65
Input 2 (ABySS)	35,112	14,383	20,904.98
GAA	46,660	63,941	99,133.42
GAM_NGS	10,971	63,152	87,930
GARM (ctg_scf)	8042	101,115	133,528.41
Metassembler	9349	57,238	78,395.6
Input 1 (MSR-CA)	18,501	32,431	46,890.24
Input 2 (SOAPdenovo)	11,556	57,117	78,228.65
GAA	Did not produce an assembly file		
GAM_NGS	12,559	59,549	89,960.46
GARM (ctg_scf)	5984	117,986	148,549.55
Metassembler	16,234	35,077	50,156.59

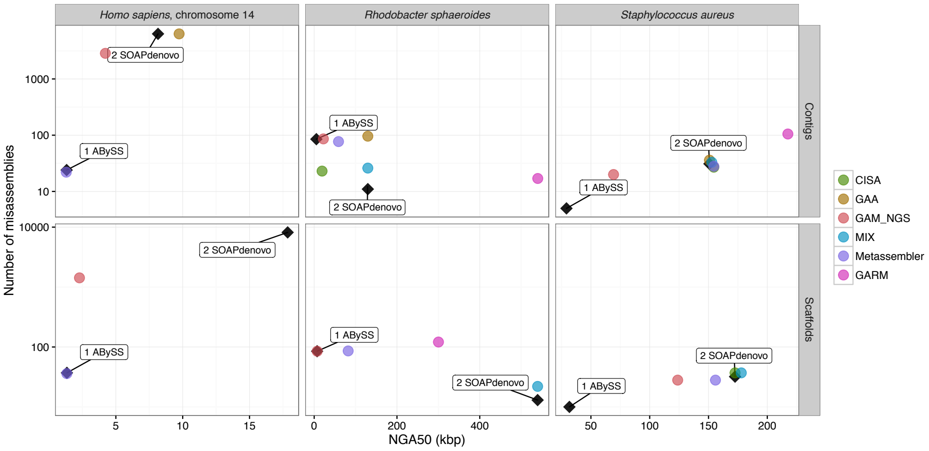

Supplemental Figure S11: Contiguity-correctness experimental results when inputs are contigs (top row) or scaffolds (bottom row); compared to Figure 2 (main text), the order of the inputs was swapped.

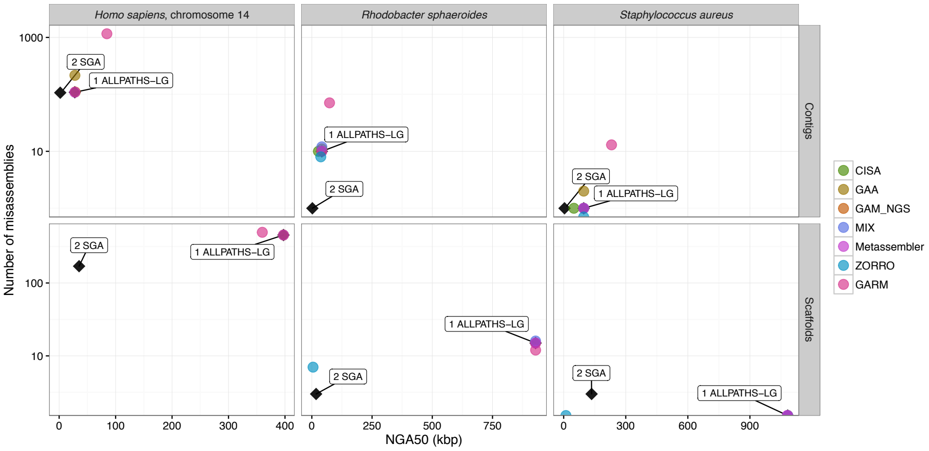

Supplemental Figure S12: Experimental results on merging assemblies produced by assemblers based on the de Bruijn graph compared to string graph (top row for input contigs, bottom row for input scaffolds); tools were ran using default parameters

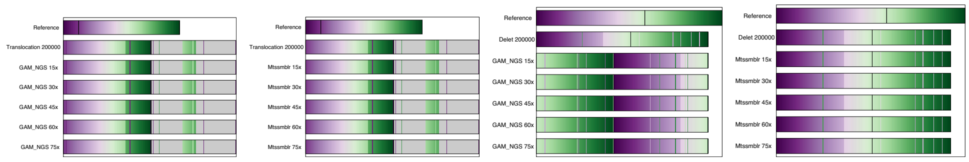

(a) GAM_NGS (b) Metassembler (c) GAM_NGS (d) Metassembler

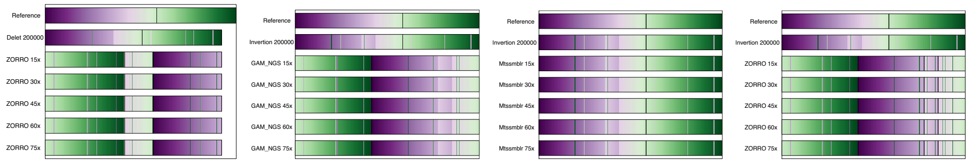

(e) ZORRO (f) GAM_NGS (g) Metassembler (h) ZORRO

Supplemental Figure S13: Assembly reconciliation results for difference choices of read coverage; (a,b) are translocations; (c,d,e) are deletions; (f,g) are inversion
